# A Systematic Review of the Biological Effects of Cordycepin

**DOI:** 10.3390/molecules26195886

**Published:** 2021-09-28

**Authors:** Masar Radhi, Sadaf Ashraf, Steven Lawrence, Asta Arendt Tranholm, Peter Arthur David Wellham, Abdul Hafeez, Ammar Sabah Khamis, Robert Thomas, Daniel McWilliams, Cornelia Huiberdina de Moor

**Affiliations:** 1 Pain Centre Versus Arthritis, University of Nottingham, Nottingham NG7 2RD, UK; masar.radhi1@nottingham.ac.uk (M.R.); asta.tranholm@nottingham.ac.uk (A.A.T.); dan.mcwilliams@nottingham.ac.uk (D.M.); 2 School of Pharmacy, University of Nottingham, Nottingham NG7 2RD, UK; steven.lawrence1@nottingham.ac.uk (S.L.); peter.wellham@nottingham.ac.uk (P.A.D.W.); abdul.hafeez@nottingham.ac.uk (A.H.); ammar.khamis@nottingham.ac.uk (A.S.K.); 3 Aberdeen Centre for Arthritis and Musculoskeletal Health, Institute of Medical Sciences, Aberdeen AB25 2ZD, UK; sadaf.ashraf@abdn.ac.uk; 4 The Primrose Oncology Unit, Bedford Hospital NHS Trust, Bedford MK42 9DJ, UK; rt@cancernet.co.uk; 5 Department of Oncology, Addenbrooke’s Cambridge University Hospitals NHS Trust, Cambridge CB2 0QQ, UK; 6 NIHR Nottingham Biomedical Research Centre (BRC), Nottingham NG5 1PB, UK

**Keywords:** cordycepin, natural product, signal transduction, AKT, mTOR, AMPK, ERK, inflammation, cell viability, review

## Abstract

We conducted a systematic review of the literature on the effects of cordycepin on cell survival and proliferation, inflammation, signal transduction and animal models. A total of 1204 publications on cordycepin were found by the cut-off date of 1 February 2021. After application of the exclusion criteria, 791 papers remained. These were read and data on the chosen subjects were extracted. We found 192 papers on the effects of cordycepin on cell survival and proliferation and calculated a median inhibitory concentration (IC_50_) of 135 µM. Cordycepin consistently repressed cell migration (26 papers) and cellular inflammation (53 papers). Evaluation of 76 papers on signal transduction indicated consistently reduced PI3K/mTOR/AKT and ERK signalling and activation of AMPK. In contrast, the effects of cordycepin on the p38 and Jun kinases were variable, as were the effects on cell cycle arrest (53 papers), suggesting these are cell-specific responses. The examination of 150 animal studies indicated that purified cordycepin has many potential therapeutic effects, including the reduction of tumour growth (37 papers), repression of pain and inflammation (9 papers), protecting brain function (11 papers), improvement of respiratory and cardiac conditions (8 and 19 papers) and amelioration of metabolic disorders (8 papers). Nearly all these data are consistent with cordycepin mediating its therapeutic effects through activating AMPK, inhibiting PI3K/mTOR/AKT and repressing the inflammatory response. We conclude that cordycepin has excellent potential as a lead for drug development, especially for age-related diseases. In addition, we discuss the remaining issues around the mechanism of action, toxicity and biodistribution of cordycepin.

## 1. Introduction

Cordycepin is the adenosine analogue 3’-deoxyadenosine, which is isolated from the caterpillar fungus *Cordyceps militaris* [1]. The caterpillar fungi are a popular health food and traditional medicine in China [2]. This interest has led to a large increase in the numbers of publications on cordycepin as a bioactive substance and potential medicine, as we illustrate in this study. The literature is fast growing, but some papers are lacking in rigour and many papers contain similar data gathered in different systems that are less than convincing on their own. In most cases, only a small selection of the available similar data are cited, hiding the repetitive nature of much of the published work. To review all the available evidence on the biological activities of cordycepin, we, therefore, chose an approach akin to the systematic reviews developed for social and clinical research, examining all relevant papers and attempting to pool data where possible. We focussed on five topics for which a lot of evidence was available: cell survival and proliferation, cell migration, cellular inflammation, signal transduction and animal models of disease. In the discussion, we indicate remaining issues that must be addressed in order for cordycepin to progress towards a medical application.

## 2. Results

### 2.1. History of the Field

**Figure 1:**
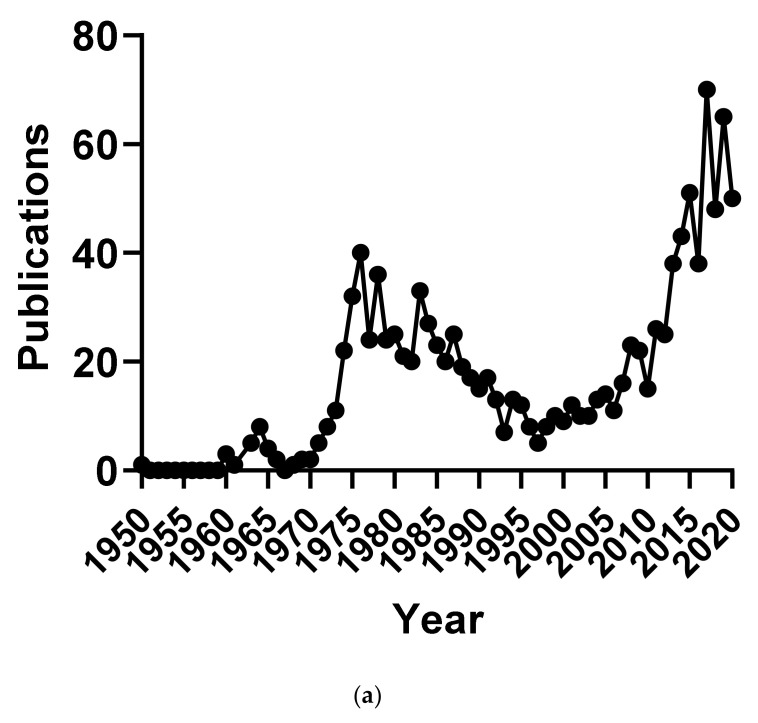

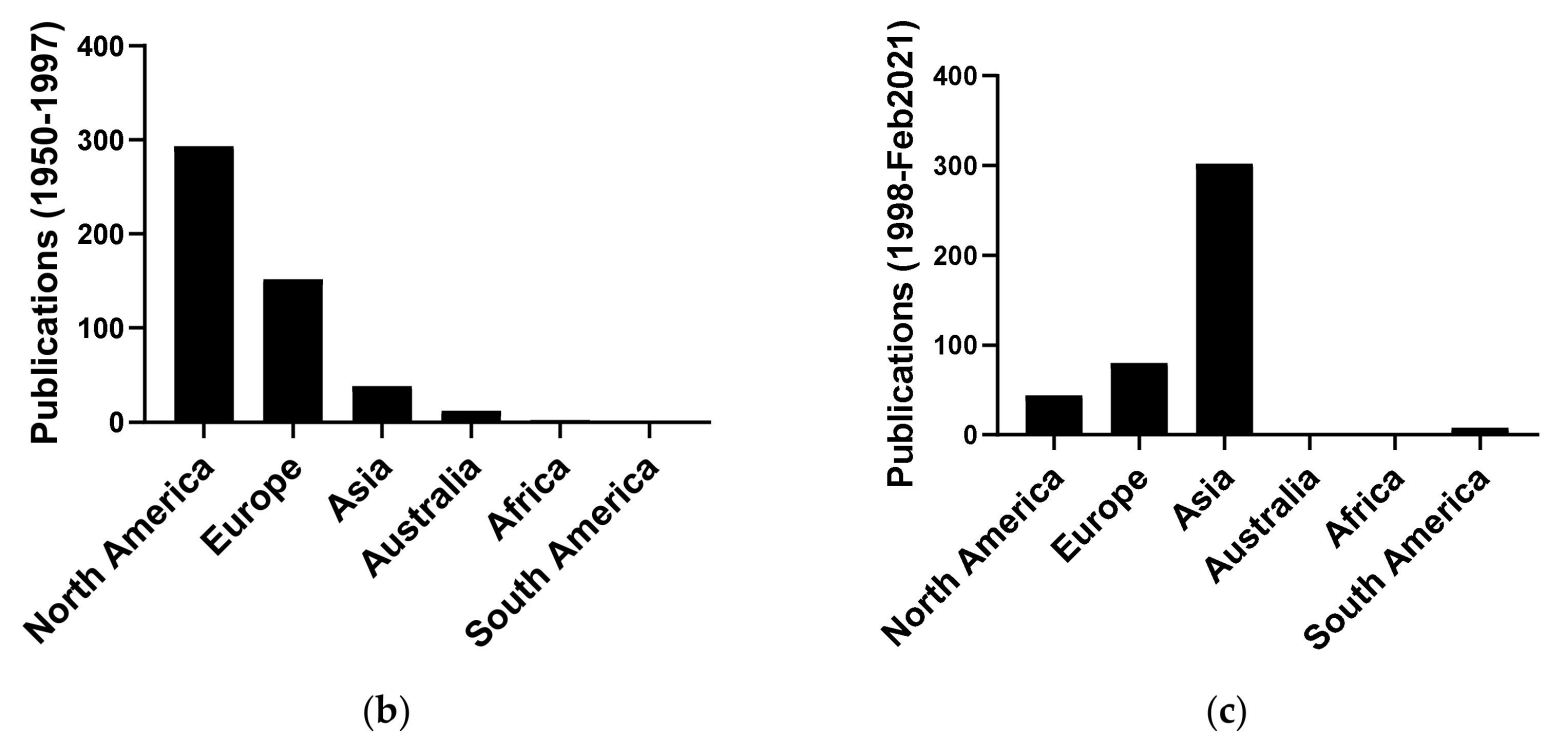
Number of publications on cordycepin by year and geographical origin. (**a**) Number of publications on cordycepin per year of publication 1950–2018; (**b,c**) Geographical distribution of the affiliation of the corresponding authors of publications on cordycepin in two periods: (**b**) 1950–1997 and (**c**) 1998–February 2021.

Cordycepin was first isolated from *Cordyceps militaris* and characterised in 1950 [1]. It was widely used as a tool to investigate RNA processing in the early days of molecular biology research, especially after it became commercially available in the early 1970s [3,4,5,6,7]. This use of the compound waned in the 1980s, when molecular biology started focussing on the study of individual genes, which had often just been cloned and sequenced. Small molecule inhibitors were becoming less popular and regarded as inadequately specific for most purposes. This led to a marked reduction in publications on cordycepin, with only five publications in our database for 1997 (Figure 1a). Since then, publications on cordycepin are on the rise again, with an especially steep increase in the last decade. It is clear this is driven by the interest in cordycepin as the active component of a traditional medicine, with the majority of publications coming from Asia, especially China (Figure 1b).

### 2.2. Cell Death, Survival and Division

Early in the study of cordycepin, it was observed that high doses can kill cells in culture and affect cell proliferation [8,9]. The idea that cordycepin may be a potential cancer treatment has led to many studies examining the doses at which cordycepin reduces cell numbers in culture. The vast majority of the data for this category is in vertebrate cells, and we concentrated exclusively on these papers for this section.

A total of 103 papers contained data on effect of cordycepin on cell numbers in vertebrate tissue culture, 65 of which attributed this cytotoxicity at least in part to the induction of apoptosis (see the Methods section). In eight cases, autophagy was reported in addition to apoptosis [10,11,12,13,14,15,16,17]. MCF-7 breast cancer cells were found to die by autophagy in one study and by apoptosis in another [18,19].

**Figure 2:**
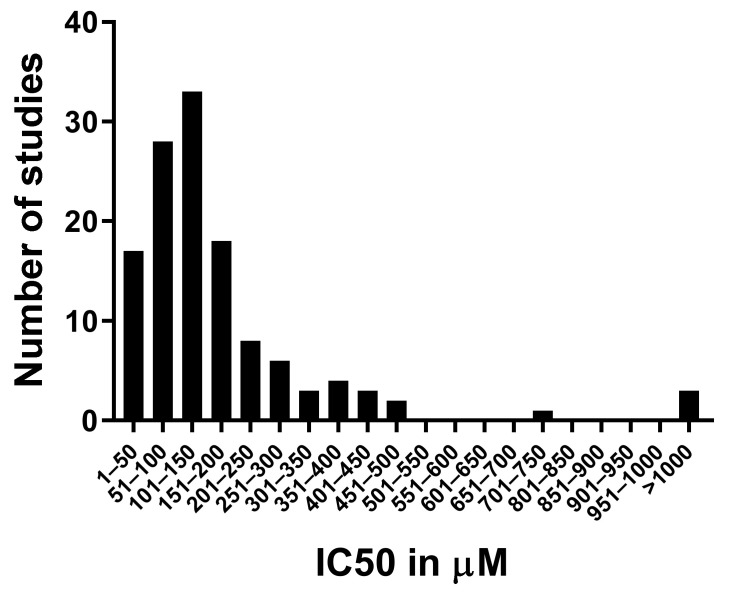
Distribution of the IC_50_ data for cordycepin from the literature. The 50% inhibitory concentration in µM was retrieved from 128 datasets described in 57 papers (listed in the Methods section). The number of datasets with an IC_50_ in each concentration bracket indicated on the Y axis was counted and graphed.

The 50% inhibitory concentration (IC_50_) is commonly used to compare the effects of cytotoxic drugs on cell lines. This number incorporates both effects on cell death and on proliferation during the incubation time. The IC_50_ concentrations for cordycepin treatment of various cell lines ranged from 15 µM to 2 mM, with the incubation time usually being 24 or 48 h. By collecting IC_50_ data for 126 experiments from 74 papers (see the Methods section), we found that the average IC_50_ is 194 µM, with a standard deviation of 250 µM, reflecting the very large variability. However, as can be seen in Figure 2, most of the IC_50_ data cluster around the median at 135 µM, with a small number of almost resistant cell lines skewing the data. A caveat of this survey is that if a cell type does not reach the IC_50_ within the dose range tried, it will not appear in our analysis, so these data are likely to have the most validity for cell types and under conditions that do exhibit cordycepin sensitivity.

Many papers report that different cell types indeed have distinct sensitivities to cordycepin [20,21,22,23,24]. In addition, we found that sometimes the same cell lines had widely different IC_50_ values in different studies; some of these may be due to differences in the method for determining viability [25,26], while others are less easy to explain [11,27]. One potential cause for the diversity is that differences in the serum used in culture medium could differentially affect the stability of cordycepin in these experiments (see the Discussion section on the pharmacokinetics of cordycepin).

In contrast, we found 45 studies reporting no or very low cytotoxic effects of cordycepin on cells in culture at concentrations at which a desirable bioactivity was evident (cited in the Methods section). The doses ranged from 40 nM to 500 µM and had a median of 50 µM. Moreover, 19 of these studies indicated that cordycepin can actively promote cell survival or prevent senescence under conditions such as ER stress, oxidative stress, radiation, etoposide exposure and other disease-related cellular stresses [28,29,30,31,32,33,34,35,36,37,38,39,40,41,42,43]. In four cases, the survival was linked to the induction of autophagy [36,37,38,42,44]. These data suggest that cordycepin is not exclusively a cytotoxic compound.

Cordycepin is known to affect the cell cycle in oocytes and early embryos, presumably through its well-characterised effects on the cytoplasmic polyadenylation of mRNAs encoding cell cycle regulators [45]. Indeed, we found 15 papers confirming these effects on vertebrate oocyte maturation and embryonic cell division [46,47,48,49,50,51,52,53,54,55,56,57,58,59,60,61].

**Figure 3:**
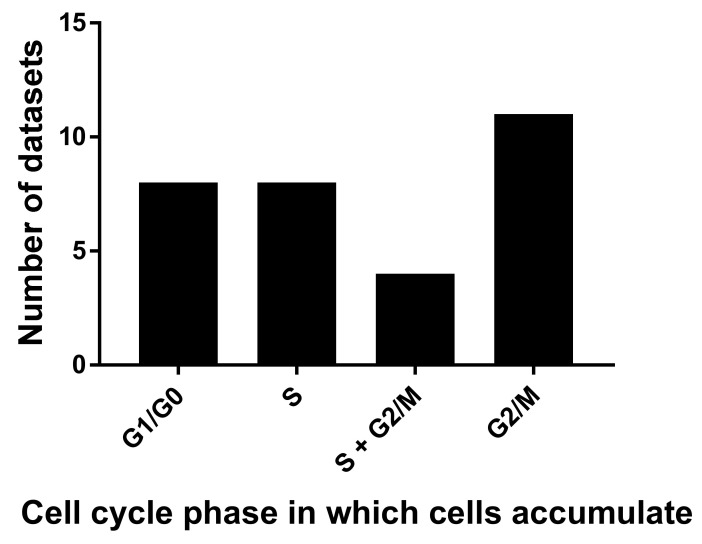
Cordycepin arrests cells in different cell cycle stages. 31 flow cytometry datasets from 18 papers were examined for the cell cycle stage in which cell numbers are significantly increased after cordycepin treatment.

The role of cytoplasmic polyadenylation in normal mitotic cell division is, however, less clear [60,62]. This is an important question, as microtubule disrupting agents, which are common in nature, affect cell cycle progression and are potent cancer drugs [63]. To determine if cordycepin has distinct effects on the mitotic cell cycle, we selected papers with flow cytometry data obtained with a fluorescent DNA stain. With this method, the phase of the cell cycle that is halted by treatment can be determined by counting cells that have unreplicated DNA (G1 or G0 phase) have undergone replication but not cell division (G2 or M phase, double the amount of DNA) or are undergoing DNA replication (S phase, intermediate amount of DNA). Classical cell cycle inhibitors cause accumulation of cells in one of these cell cycle stages. We examined 31 sets of FACS data, which matched our criteria from 20 papers for the effects of cordycepin on cell cycle progression (for details, see the Methods section). Cordycepin doses that affected the cell cycle ranged from 5 µM to 1.6 mM, with a median of 80 µM and an average of 136 µM. It is interesting that these doses are lower but similar to those observed for the IC_50_ (median 135 µM). However, as dose-response data were not widely available, it is not possible to be sufficiently quantitative for a firm conclusion. As can be seen in Figure 3, although more datasets showed arrest in G2/M, similar numbers of studies found arrest in other single stages (G0/G1: 8, S: 8, G2/M: 11) and four experiments showed accumulation in both S and G2/M phase. Indeed, in several cases, different cell cycle effects were described in a single paper and these differences were associated with cell type, dose or timing of cordycepin treatment [27,64,65,66]. It has also been reported that the effects of cordycepin are dependent on the cell cycle phase of the cell at the time of treatment [67]. We can conclude that cordycepin commonly affects cell division, but does not have the distinct stage-specific effects associated with microtubule disruptors or other cell cycle inhibitors.

### 2.3. Cell Migration

Cell migration plays a large role in normal tissue development and the function of the immune system, but it is also associated with the metastasis of cancer cells and the progression of inflammatory diseases. We surveyed the papers with data on cordycepin in vertebrates for effects of cordycepin for data on cell migration and found 27 such papers; 26 of these papers reported a repression of cell migration in a variety of cell types including cancer derived cell lines, macrophages, smooth muscle cells and endothelial cells at doses between 0.4 and 400 µM and with a median of 100 µM (see the Methods section). Only one paper reported no effect of cordycepin on cell migration in myeloid leukemic cells, but the relevant data were not included in this paper [8]. In eight cases, the reduction of migration was associated with a cordycepin-mediated repression of metalloproteinases, enzymes which can degrade extracellular matrix to allow cells migrate through tissues and can activate the TGFβ family of cytokines [68,69,70,71,72,73,74,75,76]. Just one paper reported a cordycepin-mediated increase in metalloproteinases [77]. Other frequently noted changes in connection with cell migration were a reduction in the active form of Focal Adhesion Kinase (FAK, PTK2), a key player in metastatic cancer [74,78,79,80,81], and upregulation of the epithelial marker E-cadherin, suggesting a reversal of the epithelial-mesenchymal transition [66,73,77]. Induction of NFĸB-mediated transcription by inflammatory signalling is associated with the induction of cell migration and is discussed in the section on inflammation. The combined data clearly demonstrate that cordycepin is an inhibitor of cell migration.

### 2.4. Effects on the Inflammatory Response

At the cellular level, the inflammatory response is a well-characterised set of gene expression programmes that is activated by signals indicating tissue damage or infection [82]. The induced inflammatory genes include cytokines (e.g., TNFα, IL1β and TGFβ), prostaglandin synthases (e.g., COX-2 and PTGES), nitric oxide synthase (iNOS) and genes involved in cell migration and tissue remodelling, such as the cell adhesion molecule VCAM-1, and metalloproteinases, such as MMP9 (see also above). Many cells are capable of an inflammatory response, but cells such as macrophages and microglia are most sensitive. These specialised cells respond to a larger number of stimuli and amplify the response in tissues. In a healthy situation, the inflammatory response resolves following removal of the stimulus and tissue repair. In contrast, chronic inflammation is involved in many disease processes, including cancer metastasis, the induction of chronic pain, fibrosis and neurodegeneration [83,84,85,86]. In addition, the appearance of chronic low-grade inflammation is a recognised feature of the aging process and age-related diseases [87]. Therefore, we decided to evaluate the evidence for an effect of cordycepin on the inflammatory response.

We found 38 papers which described effects of cordycepin on inflammatory gene expression (see the Methods section). Of these papers, 36 reported a reduction of inflammatory products by cordycepin. A single paper reported inhibition of iNOS but induction of TNFα [88] and only one paper reported induction of multiple inflammatory genes by cordycepin [89]. The overwhelming evidence, therefore, indicates that cordycepin has anti-inflammatory effects in tissue culture in many cell types.

TGFβ is a cytokine family which is normally involved in stem cell maintenance, wound healing and resolution of the inflammatory response [90]. Chronic expression of these peptides is associated with cancer and pathogenic tissue remodelling, for instance in osteoarthritis, chronic kidney disease, heart failure and idiopathic pulmonary fibrosis [83,91,92,93]. We found five papers that indicated that cordycepin reduces responses to TGFβ in cell culture [36,78,94,95,96] and none that reported no effect or repression. In combination with the repressive effect on the TGFβ activating metalloproteases discussed above, these data suggest that cordycepin can inhibit TGFβ activity by both reducing activation and blocking the cellular response.

NFĸB is a transcription factor with key roles in activating genes during inflammation and wound healing [82]. Activation of inflammatory signalling cascades leads to translocation of NFĸB from the cytoplasm to the nucleus and binding to DNA. We found 11 papers reporting a cordycepin-mediated reduction in the nuclear levels of NFĸB [28,43,97,98,99,100,101,102,103,104,105]. In contrast, two papers reported no changes in the nuclear localisation of NFĸB [72,106]. The total protein or mRNA levels of NFĸB subunits were found to be reduced in four studies [107,108,109,110]. DNA binding or chromatin association of NFĸB in nuclear extracts was reported to be reduced in one paper [70], but unchanged in three papers, despite clear repressed inflammatory gene expression [72,97,111]. Four papers found a reduction in the phosphorylation of NFĸB subunits [105,109,112,113]. Surprisingly, one paper reported that NFĸB binding was required for the activation of a cordycepin-induced gene, indicating that for some genes, its activity is retained [114]. The data suggest that effects of cordycepin on NFĸB-mediated transcription occur at multiple levels and are somewhat variable, probably depending on which combination of gene, cell type and stimulus is studied.

In addition, when we were examining animal studies (further discussed below) we found 28 papers that indicated that inflammatory processes were inhibited by cordycepin in a variety of animal models of disease [28,37,94,97,110,115,116,117,118,119,120,121,122,123,124,125,126,127,128,129,130,131,132,133,134,135]. Collectively, the literature indicates that cordycepin has robust anti-inflammatory activity in both cells and animals.

### 2.5. Effects on Signal Transduction Pathways

**Figure 4:**
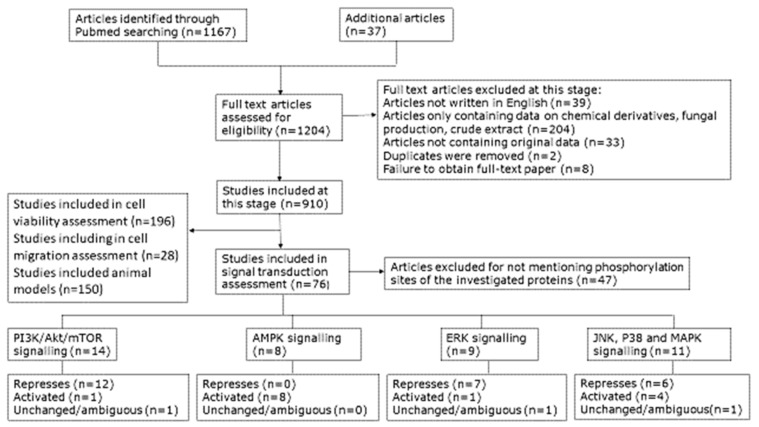
Diagram presenting the selection process for inclusion of publications in this review; n = number of articles.

**Figure 5:**
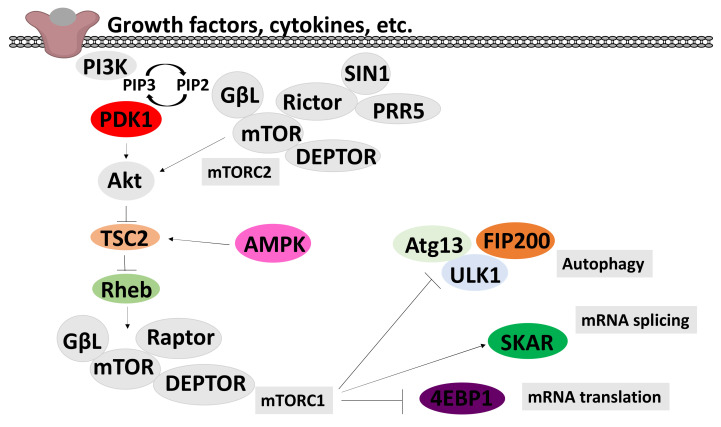
A schematic model of the PI3K/Akt/mTOR signalling pathways. This model integrates PI3K/mTOR/Akt and AMPK signal transduction pathways. Arrows indicate activation and T ends indicate inhibition. Triggered PI3K activates Akt/mTOR cascade, through activation of PDK1 and phosphorylation of AKT in the activation T-loop. mTOR complex 2 (mTORC2), is activated by an as-yet unknown pathway and contributes to the activation of AKT by phosphorylation in the C-terminal. AKT inhibits TSC2, which leads to activation of the small GTPase Rheb and activation of the mTOR complex 2 (mTORC2). The pathway is negatively regulated by AMPK, through activation of TSC2. The PI3K/Akt/mTOR signalling pathway increases protein synthesis through the phosphorylation of the cap-dependent translation inhibitor protein 4EBP1 and inhibits autophagy, leading to the promotion of growth and the anabolic state.

**Figure 6:**
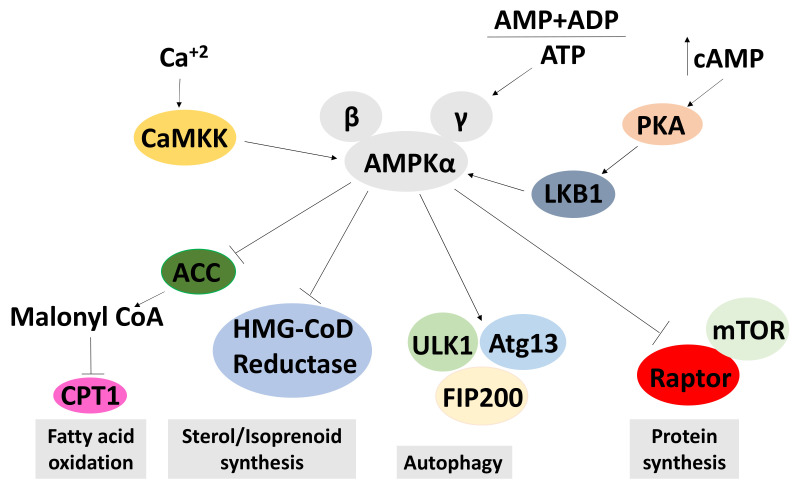
A schematic model of the AMPK signalling pathways. This model shows the activation of AMPK in response to low adenosine triphosphate (ATP) levels, and an increased adenosine diphosphate (ADP) and adenosine monophosphate (AMP). As a result, it activates pathways that produce ATP, thus increasing ATP levels. Conversely, pathways that deplete ATP are repressed by AMPK. AMPK is activated by an increased AMP + ADP to ATP ratio and phosphorylation by CAMKK or LKB1. Activated AMPK inhibits acetyl-CoA carboxylase (ACC), HMG-CoD reductase and mTORC1, leading to an increase of fatty acid oxidation and a reduction in sterol and protein synthesis. Active AMPK inhibits autophagy. An arrow indicates an upregulation of the process and a T end represents a downregulation of the process.

**Figure 7:**
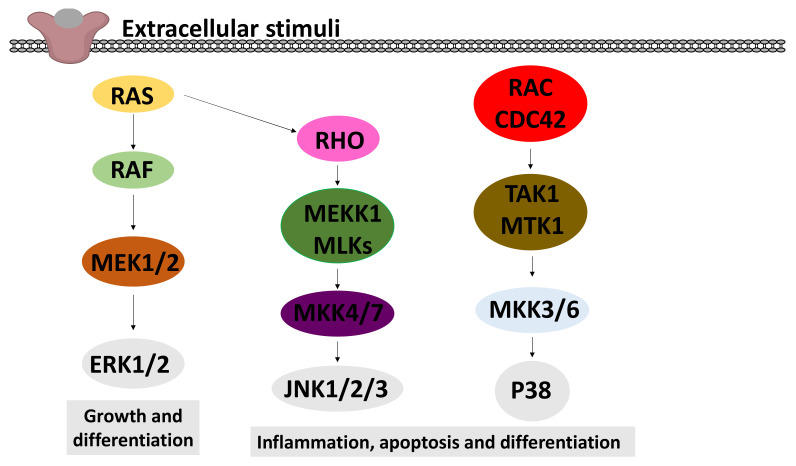
A schematic model of the MAPK signalling pathways. This model shows the three major pathways of MAPK: extracellular signal-regulated kinase (ERK) 1 and 2, c-Jun N-terminal kinases (JNK) 1–3 and p38 MAPK. In all these pathways, the activation of a small GTPase (RAS, RHO or RAC/CDC42) leads to the activation of a MAP kinase kinase kinase (MAPKKK: RAF, MEKK1/MLK, TAK/MTK1), which activates a MAP kinase kinase (MAPKK) by phosphorylation. These MAPKKs finally phosphorylate and activate the MAPKs (ERK1/2, JNK1/2/3 and p38).

A key goal of this systematic review was to obtain a clearer picture of the effect of cordycepin on signal transduction. We collected the papers studying the effects of cordycepin on signal transduction. After screening of the content, 79 papers were retrieved for detailed assessment of the effect of purified cordycepin on specific signal transduction pathways in tissue culture (Figure 4). Our assembled data showed that particular pathways were better investigated than others. The PI3K/Akt, mTOR, AMPK and MAPK signalling cascades were well represented and are depicted in Figure 5, Figure 6 and Figure 7, respectively. Disappointingly, 50 articles had to be excluded as the precise phosphorylation sites of the proteins were not indicated, neither in the description of the antibody, nor in the text of the paper, leading to ambiguity on which site was being studied. In the remaining papers, PI3K/Akt/mTOR signalling was the most commonly studied pathway (14 articles, 50%) followed by MAPK (13 articles, 43.33%) and finally AMPK (9 articles, 30%). A diverse set of cell culture models was used, the most common being various cancer cell lines, immune cells and neuronal cells. For a detailed flow chart of the study selection process including reasons for exclusion, see Figure 4.

### 2.6. Effects on the PI3K/mTOR/Akt Pathway

The phosphatidylinositol 3-kinase (PI3K) and its downstream effectors mammalian target of rapamycin (mTOR) and protein kinase B (AKT) pathways are closely intertwined pathways that regulate biological processes such as metabolic balance, growth, differentiation, cell migration and angiogenesis. They play a key role in a variety of human diseases, such as cancer, type 2 diabetes mellitus and neurodegenerative diseases, and have been implicated in aging[136,137,138,139,140]. Figure 5 shows a diagram giving the key features of this pathway.

**Figure 8:**
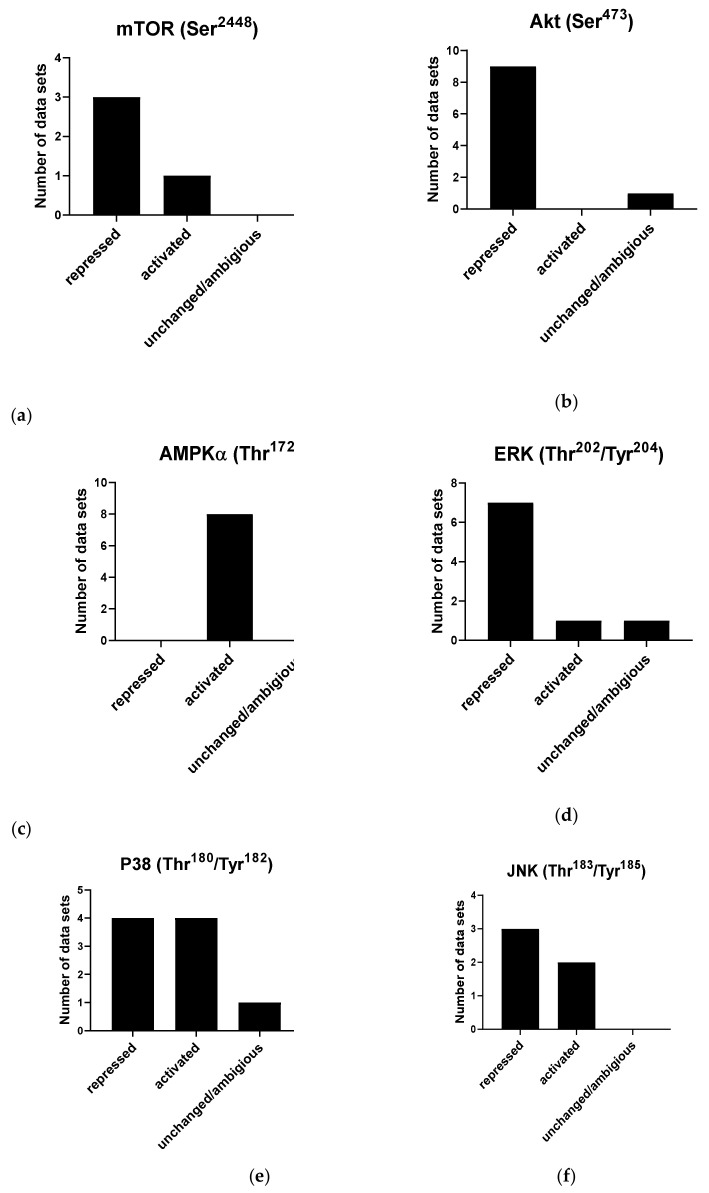
Effects of cordycepin on widely studied signal transduction pathways. Papers were selected as described in the Materials and Methods section and classified according to the effect observed on the indicated phosphorylation sites. (**a**) Effects of cordycepin on the Ser^2448^ phosphorylation site on mTOR; (**b**) Effects of cordycepin on the Ser^4738^ phosphorylation site on AKT; (**c**) Effects of cordycepin on the Thr^172^ phosphorylation site on AMPKα; (**d**) Effects of cordycepin on the Thr^202^/Tyr^204^ phosphorylation sites on ERK; (**e**) Effects of cordycepin on the Thr^180^/Tyr^182^ phosphorylation site on the p38 kinase; (**f**) Effects of cordycepin on the Thr^183^/Tyr^185^ phosphorylation site on the Jun kinase. The papers from which these data were extracted can be found in Table 1 in the Methods.

The effect of cordycepin on mTOR activity is commonly assessed by its effect on the phosphorylation of S6 kinase (S6K), which is a major mTOR downstream substrate. This phosphorylation results in an increase in S6K activity. Conversely, it has been shown that S6 kinase phosphorylates mTOR at Ser^2448^ [141], which leads to inactivation of mTOR, indicating a negative feedback loop [12,66,142,143,144,145,146]. Three of the reviewed studies found a repressive effect of cordycepin on mTOR phosphorylation at site Ser^2448^ [11,44,142]. In contrast, one paper suggested that cordycepin increases phosphorylation of mTOR at Ser^2448^ [38] (Figure 8a).

AKT phosphorylation is the most widely studied modification in cordycepin-treated cells. AKT is a family of serine/threonine protein kinases that plays a key role in cellular proliferation, apoptosis and migration. AKT kinases consist of three conserved domains: an N-terminal PH domain, a central kinase CAT domain and a C-terminal extension (EXT) containing a regulatory hydrophobic motif (HM). AKT kinases are phosphorylated by their activating kinases at a threonine residue in the activation T loop (T308 in AKT1, T309 in AKT2 and T305 in AKT3) and a Serine residue in the C-terminal HM (S473 in AKT1, S474 in AKT2 and 41 S472 in AKT3) [147,148]. AKT1 is phosphorylated at Thr^308^ by PDK1 during growth factor stimulation. For maximal activation, phosphorylation at Ser^473^ by mTORC-2 further increases its activity[147,148]. In reviewing the impact of cordycepin on the phosphorylated level of Akt, the Ser^473^ site was extensively studied (11 papers). Most studies (nine articles) demonstrated an inhibitory effect of cordycepin on Akt phosphorylation at Ser^473^ [11,101,103,142,144,149,150,151,152,153]. Only one study found no effect [145]. These data are summarised in Figure 8b. Furthermore, only two studies investigated the effect of cordycepin on Akt phosphorylation at site Thr^308^; one concluded that cordycepin has no effect on Akt activation at this site [145] and the other showed a repressive effect [154]. In addition, total Akt has been reported to be reduced [152,155]. Overall, the surveyed literature indicates that cordycepin represses Akt phosphorylation by mTOR at Ser^473^.

#### 2.6.1. Effects on AMPK Signalling

5’-adenosine monophosphate (AMP)-activated protein kinase (AMPK) is a highly conserved serine/threonine kinase. The AMPK kinase is a heterotrimeric protein complex containing a catalytic alpha subunit, an AMP-binding gamma subunit, and a scaffolding beta subunit. It is considered to be the master regulator of cellular energy homeostasis and is activated by upstream kinases, such as LKB1, CaMKKβ and Tak1 [156,157]. AMPK is allosterically regulated by the binding of AMP, or less commonly ADP, to its gamma subunit. This binding blocks the access of phosphatases and, thus, enhances the phosphorylation of Thr^172^ residue [158]. In the last decade, it has emerged that AMPK plays a central role in regulating a variety of metabolic and physiological processes. It is repressed in diverse medical conditions, such as overnutrition, inflammatory diseases, diabetes and cancer. Activation of AMPK also is the mechanism of action for the type II diabetes drug metformin. Therefore, activating AMPK could be a potential therapeutic target in treating these diseases [159,160,161].

The best-studied mechanism of AMPK activation is phosphorylation of the alpha subunit at Thr^172^, as depicted in Figure 6. AMPK activity is stimulated more than 100-fold by phosphorylation of Thr^172^ [162]. It has also been shown that phosphorylation at Ser^108^ of the beta subunit plays a key role in cell cycle regulation and promotion of pro-survival pathways in response to energy stress [163]. Although the Ser^108^ residue is auto-phosphorylated after prior phosphorylation at Thr^172^ residue, this residue is also the phosphorylation target of kinases other than AMPK such as ULK1 [163]. The literature search identified that a total of nine papers showed that cordycepin increases AMPK phosphorylation at Thr^172^ and Ser^108^, indicating activation in all cases [113,142,145,146,152,164,165,166,167]. Strikingly, we did not find a single article that suggested a repression or no effect on AMPK (Figure 8c). The literature unequivocally indicates that cordycepin treatment activates AMPK.

#### 2.6.2. Effects on MAPK Signalling

Various extracellular stimuli activate the mitogen-activated protein kinase (MAPK) cascade pathways [168]. Three distinct MAPKs have been widely investigated: p38 MAPK, c-Jun N-terminal kinases (JNK) and extracellular signal-regulated kinases (ERK), as depicted in Figure 7.

P38 proteins are a main subgroup of mitogen-activated protein kinases (MAPKs) that have been implicated in diverse biological processes, such as proliferation, differentiation, apoptosis and migration. Recent studies demonstrated that dysregulation of p38 has a key function in a wide variety of pathological conditions, such as solid tumours, arthritis and inflammation of the liver, kidney, brain and lung [169,170,171,172]. The activation of p38 is carried out by dual phosphorylation of their Thr–Gly–Tyr motif by MKK3 and MKK6 [173]. Nine studies investigating the effect of cordycepin on P38 met the eligibility criteria. Four studies showed a repressive effect [101,103,112,174], whereas four papers described that cordycepin activates P38 by increasing the phosphorylation at Thr^180^/Tyr^182^ [11,175,176,177]. A single study reported that cordycepin has no effect on P38 activation [178] (Figure 8d). Therefore, no clear trend for the effects of cordycepin treatment on p38 signalling could be discerned.

The c-Jun N-terminal kinases (JNKs) belong to the superfamily of the mitogen-activated protein kinase (MAPK) family involved in regulating eukaryotic cell reaction to a diverse range of cellular stress insults. They also coordinate essential physiological processes, including neuronal plasticity, immunological reactions, and embryonic development through their influence on gene expression, cytoskeletal protein dynamics, and cellular senescence [179,180]. Based on the search strategy employed, five articles investigating the effect of cordycepin on JNK met the inclusion criteria. Three of the studies showed a repressive effect of cordycepin on the phosphorylation of JNK at Thr^183^/Tyr^185^ [35,103,177], whilst the remaining two studies showed an activating effect on the same phosphorylation site [11,176] (Figure 8e). The effects of cordycepin treatment on JNK signalling are, therefore, ambiguous.

The extracellular signal-regulated kinases (ERK1 and ERK2) are evolutionarily conserved, highly regulated serine-threonine kinases that control cellular processes such as proliferation and differentiation. ERK plays a key role in development, and its upregulation is associated with the development and progression of diseases such as cancer. The ERK cascade is activated by signals such as growth factors, cytokines, viruses and G-protein-coupled receptor ligands [181]. The literature search yielded 38 publications related to effects of cordycepin on ERK signalling. Of these, only nine papers were included in the final analysis, as the majority did not indicate which phosphorylation sites were investigated. ERK1 and ERK2 are activated by dual phosphorylation by the upstream kinases MEK1/2 at a conserved threonine-glutamate-tyrosine (TEY) motif (Thr^202^ and Tyr^204^ in human ERK1, Thr^185^ and Tyr^187^ in human ERK2) [182]. Seven of the nine selected papers indicated an inhibitory effect of cordycepin on the phosphorylation of ERK at Thr^202^/Tyr^204^ [55,103,143,151,174,177,178]. A single article reported an activating effect of cordycepin on ERK [11], and another article reported no change [176] (Figure 8f). Overall, the literature suggests that cordycepin treatment usually inhibits the ERK pathway.

#### 2.6.3. Signal Transduction Effects in Animal Models

In reviewing the effect of cordycepin on signalling cascades in animal models (detailed below), eight articles were identified with data on signal transduction. Five of these met the eligibility criteria (see the Methods section). The outcomes were activation of AMPK indicated by increasing phosphorylation at Thr^172^ (three articles) and also inhibiting mTOR indicated by reduced the phosphorylation at Ser^2448^ (two articles), confirming the effects observed in tissue culture [35,142,164,183,184]. For details see Table 2.

### 2.7. Cordycepin Activity in Animal Models

**Figure 9:**
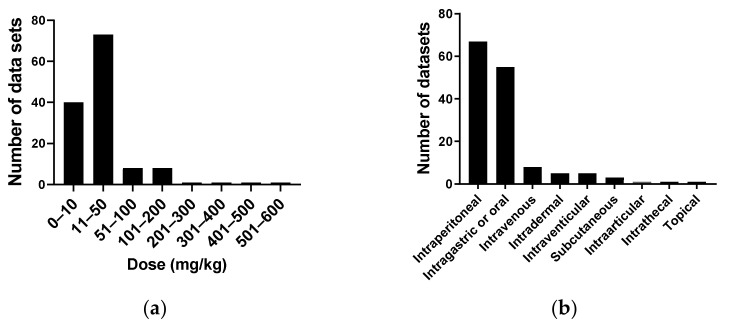
Dose of cordycepin administered to animal models in mg/kg. (**a**) A total of 133 studies were classified according to the range of cordycepin dose administered to the animal model; (**b**) The route of administration of cordycepin in 146 studies.In this systematic review, 167 articles studying the effects of purified cordycepin in a variety of animal models were found (see Table 3). We noted the details of the experimental set-up (species, model of human disease, dose in mg/kg basis and route of administration). The disease models were classified and counted (Figure 10). The majority of studies were of animal models of cancer, closely followed by cardiovascular diseases, infections and central nervous system disorders.

Figure 9a reports the doses of purified cordycepin used in 131 animal studies in mg/kg. As can be seen, most studies use doses at 50 mg/kg or less, with 39 studies using 10 mg/kg or less. A few studies use very high doses. The routes of cordycepin administration to animal models were also evaluated. Figure 9b shows that 66 studies use intraperitoneal (IP) administration, followed by oral or intragastric administration (54), with only five studies using the intravenous route.

One of the first animal studies on the effects of cordycepin examined the effect of cordycepin on the growth of tumour cells in mouse ascites [185], but the follow-up of these studies took was long. However, we identified a total of 32 studies examining the effect of cordycepin on cancer animal models. Different cancers studied included those of the immune system, gastrointestinal tract, liver, breast and lung [11,18,23,24,64,66,74,79,80,107,108,134,142,143,186,187,188,189,190,191,192,193,194,195,196,197,198,199,200,201,202,203]. In most cases, cordycepin suppressed tumour growth, but it did not cause complete regression.

Concerning the cardiovascular system, 15 articles were found for a variety of animal models. Cordycepin was reported to have beneficial effects for cardiac hypertrophy [184], ischemia [122,183,204,205,206], dyslipidaemia [37,164,207,208] and other heart disease models [76,120,209,210,211].

Fourteen articles reported that cordycepin alleviates inflammation and pain in different animal models. Furthermore, cordycepin is described to have anti-inflammatory (reduced synovial inflammation) and analgesic effects in osteoarthritic animal models and in other conditions associated with pain and inflammation. Different mechanisms were proposed to potentially explain the effect of cordycepin on pain behaviour, such as inhibition of inflammatory signalling as well as direct effects on the primary afferent nociceptors [42,97,110,121,131,133,212,213,214,215,216,217,218,219].

Several studies strongly suggest that cordycepin can be used for treatment of trypanosomiasis, but that it needs to be stabilised to do so. Treatment with cordycepin, when combined with an inhibitor of adenosine deaminase, can prolong the survival of *T. evansi*-infected animal models and confer antiparasitic activity. In addition, the in vivo and in vitro activity of synthetic cordycepin derivatives has been studied in order to explain the structure–activity relationship [130,220,221,222,223,224,225,226,227,228,229,230,231].

We found 10 papers indicating that cordycepin can have protective effects on the brain in various models of brain dysfunction. For instance, it reduces the effects of chronic unpredictable mild stress in animal models and improves depression-like behaviour [119,232]. Some studies have also shown that cordycepin ameliorates learning and memory deficits in ischemic animal models [233,234,235,236] and sleep disturbances [237]. In addition, a substantial neuroprotective effect of cordycepin was noted in Machado-Joseph disease and Parkinson disease models [28,38,116].

Effects of cordycepin on animal models of respiratory disease were reported in eight papers. Multiple studies have shown an anti-asthmatic effect, which appears to be mediated by the inhibition of inflammation [123,124,128]. Other studies showed a protective effect of cordycepin in animal models of lung injury of inflammatory origin [117,118,129] and lung fibrosis [94].

The effect of cordycepin was also studied in animal models of metabolic diseases, including diabetes and obesity. Cordycepin has been shown to reduce body weight and change fat metabolism in animal models of obesity [166,238,239,240]. Studies in models of type II diabetes found that cordycepin treatment reduced plasma glucose level, hyperphagia and polydipsia. They also showed that hepatic glycogen content was dramatically increased, and oral glucose tolerance was enhanced after cordycepin treatment. Importantly, protective effects of cordycepin against diabetes-related kidney and spleen injury have also been reported. This effect is thought to be mediated through inhibiting cellular apoptosis and fibrosis, and inducing autophagy in diabetic nephropathy [241,242,243]. Thus, cordycepin appears to have beneficial effects on metabolic disorders and their related complications.

Eight studies indicated the effects of cordycepin on reproductive disorders [244,245,246,247,248,249,250,251]. These studies investigated the effect of cordycepin on testicular function, sexual behaviour and sperm production in rodent animal models.

## 3. Discussion

Our systematic review of the literature shows that cordycepin has significant effects in many animal models of disease and is an anti-inflammatory compound in both tissue culture experiments and animal models. The repression of inflammation is likely to be one of the key events in the therapeutic effects of cordycepin. While the mechanism by which cordycepin inhibits inflammation is not fully elucidated, it appears likely that the effects on signal transduction at least contribute. mTOR/AKT and AMPK are known to be involved in the regulation of inflammation and link metabolic changes to the inflammatory response in macrophages [252,253,254,255]. In addition, many of the disease models described attempt to mimic age-related conditions, such as arthritis, type II diabetes, heart disease and neurological damage, which are indeed linked to chronic inflammation [87]. Strikingly, other mTOR inhibitors (e.g., rapamycin/sirolimus) have been reported to increase lifespan (and therefore age) in low doses. Similarly, other AMPK activators (e.g., metformin) are well documented to improve metabolic health in aging individuals [140]. It is, therefore, not as unlikely as it first might appear that cordycepin has beneficial effects in so many apparently distinct, but age-related, conditions.

The cordycepin literature is overall of modest quality, as can be observed from common study flaws, such as the lack of information on which phosphorylation sites were examined, a frequent lack of primer sequence data and the few cases of image duplication (see the Methods section). This is likely due to the combination of meagre funding but high public interest for research into natural products, leading to large numbers of relatively low-budget studies and publications which are sometimes insufficiently critically reviewed. Nevertheless, this systematic review shows that the volume of studies indicating promising biological effects is now so large that the number of replicates is making up for any noise in the data. Our meta-analysis clearly shows that cordycepin has anti-proliferative and anti-inflammatory effects and that it activates AMPK, represses phosphorylation of AKT by mTOR and often reduces phosphorylation of ERK by MEK. While it can be argued that many of the animal models that have been used do not accurately replicate human disease, there can be no doubt that cordycepin has clear beneficial effects in many animals with a variety of disease-related symptoms.

A weakness of all systematic reviews is that conclusions can only be drawn on subjects that are widely researched and the choice of these subjects is dependent on the interests of the research community. It is, therefore, possible that the key biological effects or highest therapeutic potential are not summarised in this review. Nevertheless, the findings discussed above strongly indicate that cordycepin is an excellent lead compound for drug discovery, especially in cancer and age-related diseases. Indeed, we found 18 publications describing drug development projects tackling cordycepin modification and formulation [42,120,198,218,228,256,257,258,259,260,261,262,263,264,265,266,267].

A major outstanding issue that is hampering the development of cordycepin as a lead compound is the lack of a clearly identified cordycepin-binding target molecule and a mechanism of action that connects this binding with the therapeutic effects. Proposed binding targets include poly (A) polymerases, adenosine receptors, CDK2, PARP1, AKT, AMPK, FGFR2 and RuvB-like ATPase 2 (RUVBL2) [31,32,35,40,65,66,106,145,155,167,201,210,239,268,269,270,271,272,273,274,275,276,277,278]. A recent careful evaluation of AMPK as a cordycepin target concluded that although it is bound and activated by cordycepin monophosphate, AMPK activation is not responsible for the effects of cordycepin on cell survival [166]. However, the other proposed targets still remain to be fully characterised. It is noteworthy that we found four publications indicating that cordycepin has a repressive effect on an unidentified phosphorylation site of PI3K, suggesting the effects of cordycepin on mTOR may be caused by changes in upstream signalling events [26,239,279,280]. As cordycepin is a product of evolution, it is also possible that it has multiple targets that synergise to cause the biological effects [281]. A full discussion of the outstanding issues on the target identification of cordycepin is outside the scope of this review, but the large variety of systems and the similarities of the response discussed here indicate that the main target(s) of cordycepin cannot be very cell- or tissue-specific.

Several studies showed that cordycepin is efficiently converted to cordycepin triphosphate and trapped in cells, leading to accumulation. The inhibition of import and phosphorylation of cordycepin has been shown to reduce its effects, suggesting that intracellular and phosphorylated cordycepin is indeed at least one of the active metabolites of cordycepin [35,95,106,144,152,273,274,282,283,284,285]. As indicated in our results, cordycepin has shown biological activity in animal models when administrated intraperitoneally, intravenously or orally. In blood or tissue culture media, cordycepin is rapidly deaminated by adenosine deaminase, forming 3’ deoxyinosine [165,228,286,287,288,289]. After oral administration, even sensitive assays cannot detect any cordycepin in the circulation, causing some doubts as to the active metabolite of cordycepin. However, we recently showed that 3’ deoxyinosine can be converted into cordycepin triphosphate in at least some cell types [286]. This raises the possibility that cordycepin specifically targets particular tissues in the whole organism, not because of a tissue-specific molecular target, but because of tissue-specific conversion of 3’ deoxyinosine to cordycepin triphosphate. In addition, by circulating as 3’ deoxyinosine, cordycepin may be avoiding toxic effects, which can be caused by the accumulation of adenosine-like compounds [290]. It is, therefore, very important for further drug development that biodistribution studies of cordycepin, 3’ deoxyinosine and cordycepin triphosphate are performed to resolve this issue.

Another complicating factor in cordycepin research is the lack of commercially available highly purified and/or synthetic preparations. The most widely used preparation from Sigma (now Merck) is isolated from *Cordyceps militaris* and only 98% pure. As cordycepin is generally used in micromolar quantities, it is possible that there are contaminants active in the nanomolar range that contribute to the biological effects of cordycepin. The fact that similar effects have been observed over time and with different suppliers suggests that, if there are important bioactive contaminants, these should be very consistently present. Thankfully, in a few cases, purer and synthetic cordycepin preparations have been shown to have similar effects to those observed with the standard preparations [70,239,291]. It would help the field significantly if purer cordycepin preparations became commercially available as analytical standards and for the comparison of activities.

The toxicity of cordycepin in animals has been reported to be low in the absence of adenosine deaminase inhibitors [193,207,231,292], but we did not find any publications with dose escalation studies of cordycepin to several fold the therapeutic dose for intravenous or oral administration. For intravenous doses, this is understandable, as they are limited by the solubility of cordycepin in simple formulations [257]. Three studies with cordycepin administered intraperitoneally have yielded somewhat conflicting results, with one study claiming no adverse effects at 900 mg/kg and another reporting 50% lethality at 400 mg/kg and significant deaths after 3 days of 150 mg/kg daily [185,293]. A third study indicated that three of seven animals suffered weight loss, convulsions and death after a 3.6 g/kg intraperitoneal dose, while the other four survived [243]. Ames tests suggest that cordycepin has very weak or no mutagenic activity [193,294]. Moreover, chronic oral administration of lower doses of cordycepin appears to improve rather than decrease hepatic health [127,295]. Given the effects of cordycepin on the mTOR pathway, a remaining worry is that it might suppress the immune system, as does the mTOR inhibitor rapamycin (sirolimus). On the other hand, it is worth noting that low doses of mTOR inhibitors can improve immune responses in elderly patients [296]. Similarly, the inhibition of growth factor signalling could affect wound healing, although so far, cordycepin appears to promote healing [145]. Another concern is the documented effects of cordycepin on the meiotic cell cycle and early embryogenesis (see above) may affect especially female fertility. Full dose escalation experiments and careful study of the long-term effects of therapeutic doses with an emphasis on immunity, wound healing and fertility will be important to assess the safety of cordycepin.

Promising effects in animal models do not always translate into good medicines for human patients, and we will have to await the reports of clinical trials to know if cordycepin and its derivatives are also bioactive in people (e.g., NCT00003005, NCT00709215, NCT03829254, ChiCTR-INR-17014074). A trial of cordycepin in combination with the adenosine deaminase inhibitor pentostatin for acute lymphocytic leukaemia issued a preliminary account in 2000 [194], but unfortunately, this study has still not issued a final report. Promisingly, a trial of a partially purified cordycepin preparation for patients with chronic kidney disease has reported improvements in kidney function [109].

In conclusion, cordycepin has clear biological effects in a large number of animal models of disease. It has inhibitory effects on the PI3K/AKT/mTOR signalling pathway and activates AMPK. Most therapeutic effects of cordycepin are consistent with them being mediated by these effects on signal transduction. Moreover, the wide range of therapeutic effects reported in animal models is similar to other AMPK activators and mTOR inhibitors. Remaining challenges are the obscure mechanism of action of cordycepin, the lack of commercial availability of high purity cordycepin and the incomplete understanding of cordycepin biodistribution. Nevertheless, cordycepin appears to be an excellent drug lead for many common diseases and deserves to be further investigated.

## 4. Materials and Methods

### 4.1. Scoping of the Review

Only papers recorded in PubMed were considered. A PubMed search on “cordycepin” found 1167 publications entered in PubMed by 1 February 2021. We found 37 additional papers on the subject through references in other papers and by examining the abstracts for a PubMed search for “3’-deoxyadenosine NOT cordycepin” for relevant papers, yielding 1204 papers for initial examination.

We removed 39 articles not written in English from consideration. For the remaining papers, abstracts were then read to exclude publications solely containing data on cordycepin preparation, fungal production of cordycepin, crude *Cordyceps* extracts and chemical synthesis of cordycepin. We also excluded papers only containing data on chemical derivatives of cordycepin, with the exception of studies of the intracellular metabolite cordycepin triphosphate and animal studies. These filters removed 204 papers.

A total of 33 previous reviews not containing original data were also removed from consideration. Two corrections on included papers were removed from the database. The remaining publications were collated in an excel spreadsheet. Full articles were obtained from online sources, the University of Nottingham library and through the British Library. We failed to obtain full text copies for eight papers. Data on the biological effects of cordycepin were extracted from each paper by a member of the team using a spreadsheet.

A total of 910 papers potentially containing data on the biological effects of purified cordycepin remained at this stage.

### 4.2. Selecting Sources of Data

After initial reading, we classified these papers according to the system studied (vertebrate, arthropod, other animal, fungus, plant, protists, bacteria, Archaea and cell-free systems only). Viruses were classified with their host. If more than one system was studied, the earlier listed system (as above) was entered. Notes were entered on the characteristics and species studied and themes were extracted for further analysis.

We decided to exclude the papers on plants, as cordycepin is primarily used as a transcription inhibitor to elucidate contributions of this process to biological changes (123 papers). Some differences to other transcription inhibitors have been noted in seed germination, but the nature of this difference is unclear [297].

A total of 791 other papers remained containing data on effects of cordycepin (for a full list, see Supplementary Data). The organisms studied were vertebrates (655 papers), other animals (14 papers), fungi (31 papers), protists (30 papers) and cell-free systems (31 papers). Despite the fact that the most likely natural target of cordycepin is insects, only 13 papers tackled effects on arthropods. One paper on Archaea and 16 papers on bacteria were found. In most of this review, we concentrated on the effects on vertebrates.

For the third stage, papers describing vertebrate research were read in full to find data relating to our selected subjects. For each subject, the numbers of papers found are indicated in the relevant section. For tissue culture experiments, notes were entered in a standardised Excel spreadsheet under the following standardised headings: dose, time, cell survival, cell proliferation, cell migration, signal transduction (PI3K/mTOR/AKT, MEK/ERK, p38 and JunK, others), gene expression (inflammatory and other) and other effects. For papers describing animal experiments, notes were added on the following subjects: animal model, dose, administration route, inflammation, other immune system effects, bone and cartilage, tissue remodelling and fibrosis, tumour growth, neuronal effects, metabolic effects and other effects.

### 4.3. Quantifications

#### 4.3.1. Publication Year

The original 1204 papers were used to explore the publication year of cordycepin papers. Publication year was used as stated by PubMed.

#### 4.3.2. Geographic Origin

First affiliation of the last author was determined from the PubMed entry, or if absent, by consulting the paper if it was available to us. The continent of the affiliation was entered. If the last author had affiliations in more than one continent, the first stated was used. We determined the origin for 932 papers after excluding papers with unclear affiliation.

#### 4.3.3. Cordycepin Concentrations and Cell Viability in Mammalian Tissue Culture

We found 196 papers containing data on the effects of cordycepin on cell viability, death or proliferation and these were examined further for effects on cell number, cell survival and cell cycle.

A total of 103 papers contained data on the effects of cordycepin on cell numbers in vertebrate tissue culture. Data for modified cordycepin, cordycepin with other compounds such as pentostatin, cordycepin in protective or slow-release formulations or for cells in 3D culture were excluded. Of these 103, 74 papers included cell viability experiments which used colorimetric assays with tetrazolium salts (e.g., MTT: 3-(4,5-dimethylthiazol-2-yl)-2,5-diphenyltetrazolium bromide) or employed a live/dead cell counting method, e.g., trypan blue or propidium iodide, for which the 50% inhibitory concentration (IC_50_) for cells in conventional (2D) culture was determined or could be estimated from the data for the 24 h or 48 h timepoint for one or more cell lines (48 h was selected if both were available). These studies were used for the graph in Figure 2 [11,27,66,68,70,73,81,88,99,105,107,114,117,142,143,146,149,150,151,165,178,186,192,195,196,200,202,203,256,259,264,265,267,276,279,280,298,299,300,301,302,303,304,305,306,307,308,309,310,311,312,313,314,315,316,317,318,319,320]. A total of 65 papers were found to report cell death by apoptosis [10,27,65,66,73,77,88,99,107,142,143,146,150,151,155,174,178,186,189,192,196,198,200,201,202,203,264,276,279,280,299,300,302,303,304,305,307,308,309,310,311,314,319,320,321,322,323,324,325]. We did not filter these papers by the assay or by timing.

We noted 45 papers claiming that the cytotoxic effect of cordycepin at bioactive concentrations was low. We noted the highest concentration which promoted cell survival or did not cause statistically significant cell death in these papers and determined the median and the range [28,29,30,32,33,34,35,36,37,38,39,40,41,42,43,44,69,70,80,95,98,102,105,106,117,142,145,164,184,240,270,301,315,317,326,327,328,329,330,331,332,333,334,335,336].

Papers with notes on cell proliferation were examined for descriptions of the effect of cordycepin on cell cycle in vertebrate cells. A total of 58 papers containing data on cell cycle effects were found. We identified 20 papers with data generated by DNA staining and fluorescent automated cell counting that met our criteria [24,27,65,66,73,80,132,151,176,198,202,280,314,316,317,324,337,338]. We recorded cordycepin concentration and the effects on the distribution between G1/G0, S and G2/M for each cell line as increased, decreased or no significant change and the arrested stage was classed on the basis of these changes. Papers which did not separate the three cell cycle phases or which neglected to show replicated quantified data with statistical analysis were excluded. Data for modified cordycepin or cordycepin in combination with adenosine deaminase inhibitors were also excluded.

#### 4.3.4. Cell Migration

We found data on the effect of unmodified cordycepin in solution on the migration of vertebrate cells in 28 papers. Notes were entered on these experiments, including on effects on known regulators of cell migration. The lowest dose reported to give effects in scratch or transwell assays for each cell type was obtained from 19 papers [23,66,68,69,70,71,72,73,74,76,79,80,81,88,99,196,210,322,339]. Data in which cordycepin was combined with adenosine deaminase inhibitors, siRNAs or administered in a specific formulation (e.g., nanoparticles) were excluded from this analysis.

#### 4.3.5. Cellular Inflammation

Papers selected for analysis were examined for data of effects of cordycepin on cytokines, inflammation or regulation of inflammatory genes in tissue culture. We found 54 papers with data on these subjects [28,31,36,43,69,70,71,72,76,78,88,89,94,96,97,98,99,100,101,102,103,104,105,106,107,108,109,110,111,112,113,114,117,133,270,274,286,326,328,330,332,340,341,342,343,344,345,346,347,348,349,350,351,352]. We further examined data for nine genes: the cytokines Tumour Necrosis Factor α (TNFα), Interleukin 1β (IL1β) and Transforming Growth Factor β (TGFβ), the prostaglandin synthases Prostaglandin Synthase 2 (PTGS2, also known as cyclooxygenase 2-COX-2) and prostaglandin E synthase (PTGES), the inducible nitric oxide synthase NOS2 (also known as iNOS), the Vascular Adhesion Molecule 1 (VCAM1) and the metalloproteinases MMP-3 and MMP-9. Cordycepin-induced changes in the gene products were noted as increased, unchanged or decreased. We found 38 papers describing such changes [28,69,70,71,72,76,88,89,96,97,98,99,100,101,102,103,104,106,108,109,110,112,113,117,270,286,326,330,340,341,342,343,344,345,346,347,348,349]. In addition, notes were gathered on the effects on the nuclear localisation, level, phosphorylation and DNA binding of NFĸB in the 54 papers found for this subject.

#### 4.3.6. Signal Transduction

We found 76 papers on the effect of cordycepin on cellular signalling. Papers with data about PI3K/Akt/mTOR, AMPK and MAPK signalling cascades were examined for indicating the precise phosphorylation site of the studied kinases. A total of 47 papers were excluded because we could not find or infer which phosphorylation site was studied (i.e., no mention of the site or the antibody catalogue number). A total of 29 articles were finally included in the systematic review to study the effect of cordycepin on the following kinases: mTOR [11,38,44,142], Akt [11,101,103,142,144,145,149,151,152,153,155], AMPK [113,142,145,146,152,164,166,167], ERK [11,55,103,143,151,174,176,177,178], P38 [11,101,103,112,174,175,176,177,178] and JNK [11,35,103,176,177] (Table 1). The effects were classed as repressed, activated or unchanged/ambiguous (e.g., not statistically significant or conflicting results).

**Table 1:** Overview of the included publications on signal transduction: (**a**) PI3K/Akt/mTOR; (**b**) AMPK; (**c**) ERK; (**d**) P38, JNK and MAPK signal transduction pathways.

(a)	mTOR Ser^2448^	Akt Ser^473^	Akt Thr^308^	Akt Total
Repressed	[11,44,142]	[11,101,103,142,144,149,150,151,152,153]	[154]	[152,155]
Activated	[38]			
Unchanged/ambiguous		[145]	[145]	
(b)	AMPKα Thr^172^		AMPKβ Ser^108^	
Repressed				
Activated	[113,142,145,146,152,164,166,167]		[152]	
Unchanged/ambiguous				
(c)	ERK Thr^202^/Tyr^204^			
Repressed	[55,103,143,151,174,177,178]			
Activated	[11]			
Unchanged/ambiguous	[176]			
(d)	P38 Thr^180^/Tyr^182^		JNK Thr^183^/Tyr^185^	
Repressed	[101,103,112,174]		[35,103,177]	
Activated	[11,175,176,177]		[11,176]	
Unchanged/ambiguous	[178]			

#### 4.3.7. Effects in Animal Models

We initially retrieved 160 papers studying the effect of cordycepin in diverse animal models. Ten articles were excluded because they did not use purified cordycepin or employed cordycepin derivatives only. Papers studying combination treatment with adenosine deaminase inhibitors were not excluded. Notes were entered from these papers including animal species, type of human diseases models, dose in mg/kg and route of administration. Data were also gathered regarding effects on inflammation, neuronal function and metabolism. While gathering data from papers studying the effect of cordycepin on signal transduction in animal models, we excluded any papers that did not report the precise phosphorylation site (included papers in Table 2). At this stage, the animal models were classified depending on the human diseases and non-diseased models were excluded. The classes were as following depending on the number of publications in each class: cancer (breast cancer, liver cancer, glioma and leukaemia), cardiovascular diseases (dyslipidaemia, cardiac hypertrophy and hypertension), infection, central nervous system disorders (depressive disorders and learning disorders), respiratory diseases (asthma), reproductive disorders, metabolic disorders, bone disorders (osteoporosis and osteoarthritis), endocrine disorders, pain in hyperalgesic priming (a model of transition from acute to chronic pain), inflammation, hepatic diseases, aging (such as age-related sexual dysfunction and oxidative stress), skin disorders and wound healing (Table 3 specifies the allocation). If two relevant classes were identified in one animal model, the classification was assigned to the first field listed. For example, in papers studying the effect of cordycepin on osteoarthritic animal models where pain was also measured, the study was categorised under bone disorders. The range of cordycepin dose administered to the animal was examined in 131 papers, in which the cordycepin dose could be calculated as mg/kg [11,18,23,24,28,37,38,42,64,66,76,79,80,97,107,108,119,120,121,122,126,130,134,142,143,145,150,164,183,184,185,186,187,188,190,191,192,193,194,195,196,197,198,199,202,204,205,206,207,208,209,210,211,212,213,214,215,216,218,219,220,221,222,223,224,225,226,227,228,229,230,231,232,233,234,236,237,293,328,353,354,355].

**Table 2:** Overview of the included publications studying signal transduction pathways in animal models.

	mTOR Ser^2448^	Akt Ser^473^	AMPKα Thr^172^	ERK Thr^202^/Tyr^204^	JNK Thr^183^/Tyr^185^
Repressed	[142,184]	[142]		[184]	[35]
Activated		[183]	[142,164,184]		
Unchanged/Ambiguous					

**Table 3:** Classification of animal models treated with cordycepin. The animal models are classified according to the type of human disease.

Animal Model	Publications
Cancer	[11,18,23,24,64,66,74,79,80,107,108,134,142,143,185,186,187,188,189,190,191,192,193,194,195,196,197,198,199,200,201,202,203]
Cardiovascular	[37,76,120,122,164,183,184,204,206,207,208,209,211]
Infection	[220,221,222,223,224,225,226,227,228,229,230,231]
Central Nervous System	[28,38,116,119,232,233,234,235,236,237]
Respiratory Diseases	[94,117,118,123,124,128,129]
Reproductive Disorders	[244,245,246,247,248,249,250,251]
Metabolic Disorders	[166,238,239,240]
Bone	[59,126,334,342]
Inflammation/Pain	[42,97,110,121,131,133,212,213,214,215,216,217,218,219]

#### 4.3.8. Exclusion of Papers with Image Duplication

During this study, a key paper was retracted because of extensive data duplication (PubMed ID 32764880), and we became aware of the problem of so-called paper mill publications. To try to avoid including any so-far undetected paper mill publications, we examined all articles we had selected for inclusion in the review on duplicated images. No problems on a scale as large as the first paper were encountered, but we found three more papers with clear duplication of one panel. While these may be due to honest mistakes, it shows insufficient care and these papers were removed from consideration. We notified the publishers of our concerns. In two other cases, two images were very similar, but not identical, and we decided to include these but also notify the publishers of these papers.

## Figures and Tables

**Figure 10:**
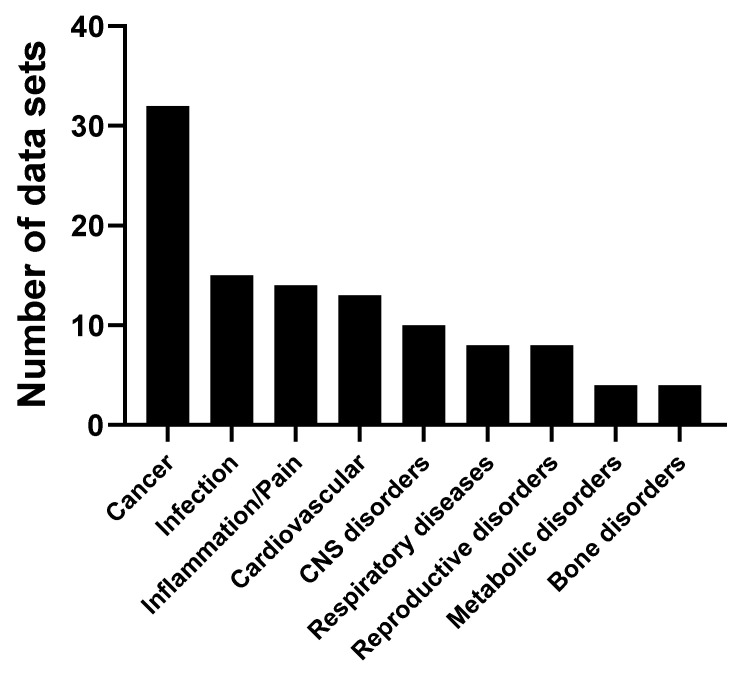
Classification of animal models. A total of 139 articles were classified according to the type of human disease modelled. CNS: central nervous system. Categories which had only one entry were excluded, as described in the Methods section. The exact allocation of papers to disease classes can be found in the Methods section (Table 3).

## Data Availability

All data in the article were extracted from the cited papers as outlined in the Methods section.

## Supplementary Materials

The Supplementary Data are available online.

## References

[1] Cunningham K., Manson W., Spring F., Hutchinson S. (1950). Cordycepin, a metabolic product isolated from cultures of Cordyceps militaris (Linn.) Link. Nature.

[2] Winkler D. (2010). Cordyceps sinensis—a precious parasitic fungus infecting Tibet. Field Mycol..

[3] Penman S., Rosbash M., Penman M. (1970). Messenger and heterogeneous nuclear RNA in HeLa cells: Differential inhibition by cordycepin. Proc. Natl. Acad. Sci. USA.

[4] Philipson L., Wall R., Glickman G., Darnell J. (1971). Addition of polyadenylate sequences to virus-specific RNA during adenovirus replication. Proc. Natl. Acad. Sci. USA.

[5] Wu A., Ting R., Paran M., Gallo R. (1972). Cordycepin inhibits induction of murine leukovirus production by 5-iodo-2′-deoxyuridine. Proc. Natl. Acad. Sci. USA.

[6] Brawerman G., Diez J. (1975). Metabolism of the polyadenylate sequence of nuclear RNA and messenger RNA in mammalian cells. Cell.

[7] Diez J., Brawerman G. (1974). Elongation of the polyadenylate segment of messenger RNA in the cytoplasm of mammalian cells. Proc. Natl. Acad. Sci. USA.

[8] Lotem J., Sachs L. (1975). Induction of specific changes in the surface membrane of myeloid leukemic cells by steroid hormones. Int. J. Cancer.

[9] Price P.J., Suk W.A., Peters R.L., Martin C.E., Bellew T.M., Huebner R.J. (1975). Cordycepin inhibition of 3-methylcholanthrene-induced transformation in vitro. Proc. Soc. Exp. Biol. Med..

[10] Li Y., Li R., Zhu S., Zhou R., Wang L., Du J., Wang Y., Zhou B., Mai L. (2015). Cordycepin induces apoptosis and autophagy in human neuroblastoma SK-N-SH and BE (2)-M17 cells. Oncol. Lett..

[11] Pan B.-S., Wang Y.-K., Lai M.-S., Mu Y.-F., Huang B.-M. (2015). Cordycepin induced MA-10 mouse Leydig tumor cell apoptosis by regulating p38 MAPKs and PI3K/AKT signaling pathways. Sci. Rep..

[12] Yu X., Ling J., Liu X., Guo S., Lin Y., Liu X., Su L. (2017). Cordycepin induces autophagy-mediated c-FLIPL degradation and leads to apoptosis in human non-small cell lung cancer cells. Oncotarget.

[13] Lee H.H., Kim S.O., Kim G.-Y., Moon S.-K., Kim W.-J., Jeong Y.K., Yoo Y.H., Choi Y.H. (2014). Involvement of autophagy in cordycepin-induced apoptosis in human prostate carcinoma LNCaP cells. Environ. Toxicol. Pharmacol..

[14] Choi S., Lim M.-H., Kim K.M., Jeon B.H., Song W.O., Kim T.W. (2011). Cordycepin-induced apoptosis and autophagy in breast cancer cells are independent of the estrogen receptor. Toxicol. Appl. Pharmacol..

[15] Chaicharoenaudomrung N., Jaroonwitchawan T., Noisa P. (2018). Cordycepin induces apoptotic cell death of human brain cancer through the modulation of autophagy. Toxicol. Vitr..

[16] Ho S.-Y., Wu W.-S., Lin L.-C., Wu Y.-H., Chiu H.-W., Yeh Y.-L., Huang B.-M., Wang Y.-J. (2019). Cordycepin enhances radiosensitivity in oral squamous carcinoma cells by inducing autophagy and apoptosis through cell cycle arrest. Int. J. Mol. Sci..

[17] Jang H.-J., Yang K.E., Hwang I.-H., Huh Y.H., Kim D.J., Yoo H.-S., Park S.J., Jang I.-S. (2019). Cordycepin inhibits human ovarian cancer by inducing autophagy and apoptosis through Dickkopf-related protein 1/β-catenin signaling. Am. J. Transl. Res..

[18] Wang D., Zhang Y., Lu J., Wang Y., Wang J., Meng Q., Lee R.J., Wang D., Teng L. (2016). Cordycepin, a natural antineoplastic agent, induces apoptosis of breast cancer cells via caspase-dependent pathways. Nat. Prod. Commun..

[19] Lee D., Lee W.-Y., Jung K., Kwon Y.S., Kim D., Hwang G.S., Kim C.-E., Lee S., Kang K.S. (2019). The inhibitory effect of cordycepin on the proliferation of MCF-7 breast cancer cells, and its mechanism: An investigation using network pharmacology-based analysis. Biomolecules.

[20] He W., Zhang M.-f., Ye J., Jiang T.-t., Fang X., Song Y. (2010). Cordycepin induces apoptosis by enhancing JNK and p38 kinase activity and increasing the protein expression of Bcl-2 pro-apoptotic molecules. J. Zhejiang Univ. Sci. B.

[21] Lee H.H., Park C., Jeong J.-W., Kim M.J., Seo M.J., Kang B.W., Park J.U., Kim G.-Y., Choi B.T., Choi Y.H. (2013). Apoptosis induction of human prostate carcinoma cells by cordycepin through reactive oxygen species-mediated mitochondrial death pathway. Int. J. Oncol..

[22] Baik J.-S., Mun S.-W., Kim K.-S., Park S.-J., Yoon H.-K., Kim D.-H., Park M.-K., Kim C.-H., Lee Y.-C. (2016). Apoptotic effects of cordycepin through the extrinsic pathway and p38 MAPK activation in human glioblastoma U87MG cells. J. Microbiol. Biotechnol..

[23] Su N.-W., Wu S.-H., Chi C.-W., Liu C.-J., Tsai T.-H., Chen Y.-J. (2017). Metronomic cordycepin therapy prolongs survival of oral cancer-bearing mice and inhibits epithelial-mesenchymal transition. Molecules.

[24] Wang X.-A., Xiang S.-S., Li H.-F., Wu X.-S., Li M.-L., Shu Y.-J., Zhang F., Cao Y., Ye Y.-Y., Bao R.-F. (2014). Cordycepin induces S phase arrest and apoptosis in human gallbladder cancer cells. Molecules.

[25] Jeong J.-W., Jin C.-Y., Park C., Hong S.H., Kim G.-Y., Jeong Y.K., Lee J.-D., Yoo Y.H., Choi Y.H. (2011). Induction of apoptosis by cordycepin via reactive oxygen species generation in human leukemia cells. Toxicol. Vitr..

[26] Jang K.-J., Kwon G.-S., Jeong J.-W., Kim C.-H., Yoon H.-M., Kim G.-Y., Shim J.-H., Moon S.-K., Kim W.-J., Choi Y.H. (2015). Cordyceptin induces apoptosis through repressing hTERT expression and inducing extranuclear export of hTERT. J. Biosci. Bioeng..

[27] Jen C.-Y., Lin C.-Y., Huang B.-M., Leu S.-F. (2011). Cordycepin induced MA-10 mouse Leydig tumor cell apoptosis through caspase-9 pathway. Evid. -Based Complementary Altern. Med..

[28] Cheng C., Zhu X. (2019). Cordycepin mitigates MPTP-induced Parkinson’s disease through inhibiting TLR/NF-κB signaling pathway. Life Sci..

[29] Comella J.X., Sanz-Rodriguez C., Aldea M., Esquerda J.E. (1994). Skeletal muscle-derived trophic factors prevent motoneurons from entering an active cell death program in vitro. J. Neurosci..

[30] D’Arpa P., Beardmore C., Liu L.F. (1990). Involvement of nucleic acid synthesis in cell killing mechanisms of topoisomerase poisons. Cancer Res..

[31] Gu L., Johno H., Nakajima S., Kato H., Takahashi S., Katoh R., Kitamura M. (2013). Blockade of Smad signaling by 3′-deoxyadenosine: A mechanism for its anti-fibrotic potential. Lab. Investig..

[32] Jin M.L., Park S.Y., Kim Y.H., Oh J.-I., Lee S.J., Park G. (2014). The neuroprotective effects of cordycepin inhibit glutamate-induced oxidative and ER stress-associated apoptosis in hippocampal HT22 cells. Neurotoxicology.

[33] Kan H., Wang Y., Wang D., Sun H., Zhou S., Wang H., Guan J., Li M. (2018). Cordycepin rescues lidocaine-induced neurotoxicity in dorsal root ganglion by interacting with inflammatory signaling pathway MMP3. Eur. J. Pharmacol..

[34] Kaufmann S.H. (1991). Antagonism between camptothecin and topoisomerase II-directed chemotherapeutic agents in a human leukemia cell line. Cancer Res..

[35] Kitamura M., Kato H., Saito Y., Nakajima S., Takahashi S., Johno H., Gu L., Katoh R. (2011). Aberrant, differential and bidirectional regulation of the unfolded protein response towards cell survival by 3’-deoxyadenosine. Cell Death Differ.

[36] Li L., He D., Yang J., Wang X. (2011). Cordycepin inhibits renal interstitial myofibroblast activation probably by inducing hepatocyte growth factor expression. J. Pharmacol. Sci..

[37] Li X., Zhou Y., Zhang X., Cao X., Wu C., Guo P. (2017). Cordycepin stimulates autophagy in macrophages and prevents atherosclerotic plaque formation in ApoE-/-mice. Oncotarget.

[38] Marcelo A., Brito F., Carmo-Silva S., Matos C.A., Alves-Cruzeiro J., Vasconcelos-Ferreira A., Koppenol R., Mendonça L., de Almeida L.P., Nóbrega C. (2019). Cordycepin activates autophagy through AMPK phosphorylation to reduce abnormalities in Machado–Joseph disease models. Hum. Mol. Genet..

[39] Schneider E., Lawson P.A., Ralph R.K. (1989). Inhibition of protein synthesis reduces the cytotoxicity of 4’-(9-acridinylamino) methane-sulfon-m-anisidide without affecting DNA breakage and DNA topoisomerase ii in a murine mastocytoma cell line. Biochem. Pharmacol..

[40] Song H., Huang L.-P., Li Y., Liu C., Wang S., Meng W., Wei S., Liu X.-P., Gong Y., Yao L.-H. (2018). Neuroprotective effects of cordycepin inhibit Aβ-induced apoptosis in hippocampal neurons. Neurotoxicology.

[41] Wotring L.L., Townsend L.B. (1989). Identification of 6-azauridine triphosphate in l1210 cells and its possible relevance to cytotoxicity. Cancer Res..

[42] Xia C., Chen P., Mei S., Ning L., Lei C., Wang J., Zhang J., Ma J., Fan S. (2017). Photo-crosslinked HAMA hydrogel with cordycepin encapsulated chitosan microspheres for osteoarthritis treatment. Oncotarget.

[43] Yang J., Cao Y., Lv Z., Jiang T., Wang L., Li Z. (2015). Cordycepin protected against the TNF-α-induced inhibition of osteogenic differentiation of human adipose-derived mesenchymal stem cells. Int. J. Immunopathol. Pharmacol..

[44] Li T., Wen L., Cheng B. (2019). Cordycepin alleviates hepatic lipid accumulation by inducing protective autophagy via PKA/mTOR pathway. Biochem. Biophys. Res. Commun..

[45] Charlesworth A., Meijer H.A., de Moor C.H. (2013). Specificity factors in cytoplasmic polyadenylation. Wiley Interdiscip. Rev. Rna.

[46] Kuge H., Inoue A. (1992). Maturation of Xenopus laevis oocyte by progesterone requires poly (A) tail elongation of mRNA. Exp. Cell Res..

[47] Nagahama Y. (1997). 17α, 20β-Dihydroxy-4-pregnen-3-one, a maturation-inducing hormone in fish oocytes: Mechanisms of synthesis and action. Steroids.

[48] Barkoff A., Ballantyne S., Wickens M. (1998). Meiotic maturation in Xenopus requires polyadenylation of multiple mRNAs. Embo J..

[49] Katsu Y., Yamashita M., Nagahama Y. (1999). Translational regulation of cyclin B mRNA by 17α, 20β-dihydroxy-4-pregnen-3-one (maturation-inducing hormone) during oocyte maturation in a teleost fish, the goldfish (Carassius auratus). Mol. Cell. Endocrinol..

[50] Fuchimoto D.-i., Mizukoshi A., Schultz R.M., Sakai S., Aoki F. (2001). Posttranscriptional regulation of cyclin A1 and cyclin A2 during mouse oocyte meiotic maturation and preimplantation development. Biol. Reprod..

[51] Faerge I., Terry B., Kalous J., Wahl P., Lessl M., Ottesen J., Hyttel P., Grøndahl C. (2001). Resumption of meiosis induced by meiosis-activating sterol has a different signal transduction pathway than spontaneous resumption of meiosis in denuded mouse oocytes cultured in vitro. Biol. Reprod..

[52] Traverso J.M., Donnay I., Lequarre A.S. (2005). Effects of polyadenylation inhibition on meiosis progression in relation to the polyadenylation status of cyclins A2 and B1 during in vitro maturation of bovine oocytes. Mol. Reprod. Dev. Inc. Gamete Res..

[53] Hara K.T., Oda S., Naito K., Nagata M., Schultz R.M., Aoki F. (2005). Cyclin A2-CDK2 regulates embryonic gene activation in 1-cell mouse embryos. Dev. Biol..

[54] Gershon E., Galiani D., Dekel N. (2006). Cytoplasmic polyadenylation controls cdc25B mRNA translation in rat oocytes resuming meiosis. Reproduction.

[55] Zhang D.X., Cui X.S., Kim N.H. (2009). Involvement of polyadenylation status on maternal gene expression during in vitro maturation of porcine oocytes. Mol. Reprod. Dev..

[56] Dobbs K.B., Spollen W.G., Springer G., Prather R.S. (2010). The role of cytoplasmic polyadenylation element sequence on mRNA abundance during porcine embryogenesis and parthenogenetic development. Mol. Reprod. Dev..

[57] Liu H., Gao Y., Zhai B., Jiang H., Ding Y., Zhang L., Li C., Deng Q., Yu X., Zhang J. (2016). The Effects of polyadenylation status on MPFs during in vitro porcine oocyte maturation. Cell. Physiol. Biochem..

[58] Nair R., Victor A.C., Paul V., Paul-Prasanth B. (2017). Effects of N-Nitrosodiethylamine, a Potent Carcinogen, on Sexual Development, Gametogenesis, and Oocyte Maturation. Sex. Dev..

[59] Krischek C., Meinecke B. (2002). In vitro maturation of bovine oocytes requires polyadenylation of mRNAs coding proteins for chromatin condensation, spindle assembly, MPF and MAP kinase activation. Anim. Reprod. Sci..

[60] Novoa I., Gallego J., Ferreira P.G., Mendez R. (2010). Mitotic cell-cycle progression is regulated by CPEB1 and CPEB4-dependent translational control. Nat. Cell Biol..

[61] Osborn J., Moor R. (1983). Time-dependent effects of α-amanitin on nuclear maturation and protein synthesis in mammalian oocytes. J. Embryol. Exp. Morphol..

[62] Park J.-E., Yi H., Kim Y., Chang H., Kim V.N. (2016). Regulation of poly (A) tail and translation during the somatic cell cycle. Mol. Cell.

[63] Čermák V., Dostál V., Jelínek M., Libusová L., Kovář J., Rösel D., Brábek J. (2020). Microtubule-targeting agents and their impact on cancer treatment. Eur. J. Cell Biol..

[64] Su N.-W., Wu S.-H., Chi C.-W., Tsai T.-H., Chen Y.-J. (2019). Cordycepin, isolated from medicinal fungus Cordyceps sinensis, enhances radiosensitivity of oral cancer associated with modulation of DNA damage repair. Food Chem. Toxicol..

[65] Thomadaki H., Tsiapalis C.M., Scorilas A. (2008). The effect of the polyadenylation inhibitor cordycepin on human Molt-4 and Daudi leukaemia and lymphoma cell lines. Cancer Chemother. Pharmacol..

[66] Wei C., Yao X., Jiang Z., Wang Y., Zhang D., Chen X., Fan X., Xie C., Cheng J., Fu J. (2019). Cordycepin inhibits drug-resistance non-small cell lung cancer progression by activating AMPK signaling pathway. Pharmacol. Res..

[67] Tomasovic S.P., Dewey W.C. (1978). Acceleration of CHO cells into mitosis and reduction of X-ray-induced G2 delay by cordycepin. Exp. Cell Res..

[68] Jeong J.-W., Jin C.-Y., Park C., Han M.H., Kim G.-Y., Moon S.-K., Kim C.G., Jeong Y.K., Kim W.-J., Lee J.-D. (2012). Inhibition of migration and invasion of LNCaP human prostate carcinoma cells by cordycepin through inactivation of Akt. Int. J. Oncol..

[69] Jeong J.-W., Park C., Cha H.-J., Hong S.H., Park S.-H., Kim G.-Y., Kim W.J., Kim C.H., Song K.S., Choi Y.H. (2018). Cordycepin inhibits lipopolysaccharide-induced cell migration and invasion in human colorectal carcinoma HCT-116 cells through down-regulation of prostaglandin E2 receptor EP4. Bmb Rep..

[70] Lee E.J., Kim W.J., Moon S.K. (2010). Cordycepin suppresses TNF-alpha-induced invasion, migration and matrix metalloproteinase-9 expression in human bladder cancer cells. Phytother. Res..

[71] Li Y., Li K., Mao L., Han X., Zhang K., Zhao C., Zhao J. (2016). Cordycepin inhibits LPS-induced inflammatory and matrix degradation in the intervertebral disc. PeerJ.

[72] Noh E.-M., Jung S.H., Han J.-H., Chung E.-Y., Jung J.-Y., Kim B.-S., Lee S.-H., Lee Y.-R., Kim J.-S. (2010). Cordycepin inhibits TPA-induced matrix metalloproteinase-9 expression by suppressing the MAPK/AP-1 pathway in MCF-7 human breast cancer cells. Int. J. Mol. Med..

[73] Wang Y., Lv Y., Liu T.S., Di Yan W., Chen L.Y., Li Z.H., Piao Y.S., An R.B., Lin Z.H., Ren X.S. (2019). Cordycepin suppresses cell proliferation and migration by targeting CLEC2 in human gastric cancer cells via Akt signaling pathway. Life Sci..

[74] Zhang P., Huang C., Fu C., Tian Y., Hu Y., Wang B., Strasner A., Song Y., Song E. (2015). Cordycepin (3′-deoxyadenosine) suppressed HMGA2, Twist1 and ZEB1-dependent melanoma invasion and metastasis by targeting miR-33b. Oncotarget.

[75] Yu Q., Stamenkovic I. (2000). Cell surface-localized matrix metalloproteinase-9 proteolytically activates TGF-β and promotes tumor invasion and angiogenesis. Genes Dev..

[76] Chang W., Lim S., Song H., Song B.-W., Kim H.-J., Cha M.-J., Sung J.M., Kim T.W., Hwang K.-C. (2008). Cordycepin inhibits vascular smooth muscle cell proliferation. Eur. J. Pharmacol..

[77] Tao X., Ning Y., Zhao X., Pan T. (2016). The effects of cordycepin on the cell proliferation, migration and apoptosis in human lung cancer cell lines A549 and NCI-H460. J. Pharm. Pharmacol..

[78] Cao Z., Dou C., Li J., Tang X., Xiang J., Zhao C., Zhu L., Bai Y., Xiang Q., Dong S. (2016). Cordycepin inhibits chondrocyte hypertrophy of mesenchymal stem cells through PI3K/Bapx1 and Notch signaling pathway. Bmb Rep..

[79] Hueng D.-Y., Hsieh C.-H., Cheng Y.-C., Tsai W.-C., Chen Y. (2017). Cordycepin inhibits migration of human glioblastoma cells by affecting lysosomal degradation and protein phosphatase activation. J. Nutr. Biochem..

[80] Lin Y.-T., Liang S.-M., Wu Y.-J., Wu Y.-J., Lu Y.-J., Jan Y.-J., Ko B.-S., Chuang Y.-J., Shyue S.-K., Kuo C.-C. (2019). Cordycepin suppresses endothelial cell proliferation, migration, angiogenesis, and tumor growth by regulating focal adhesion kinase and p53. Cancers.

[81] Yao W.-L., Ko B.-S., Liu T.-A., Liang S.-M., Liu C.-C., Lu Y.-J., Tzean S.-S., Shen T.-L., Liou J.-Y. (2014). Cordycepin suppresses integrin/FAK signaling and epithelial-mesenchymal transition in hepatocellular carcinoma. Anti-Cancer Agents Med. Chem. (Former. Curr. Med. Chem. -Anti-Cancer Agents).

[82] Mitchell J.P., Carmody R.J. (2018). NF-κB and the transcriptional control of inflammation. Int. Rev. Cell Mol. Biol..

[83] Khandia R., Munjal A. (2020). Interplay between inflammation and cancer. Adv. Protein Chem. Struct. Biol..

[84] Stewart A.G., Thomas B., Koff J. (2018). TGF-β: Master regulator of inflammation and fibrosis. Respirology.

[85] Ji R.-R., Chamessian A., Zhang Y.-Q. (2016). Pain regulation by non-neuronal cells and inflammation. Science.

[86] Heppner F.L., Ransohoff R.M., Becher B. (2015). Immune attack: The role of inflammation in Alzheimer disease. Nat. Rev. Neurosci..

[87] Wyss-Coray T. (2016). Ageing, neurodegeneration and brain rejuvenation. Nature.

[88] Hwang J.H., Park S.J., Ko W.G., Kang S.-M., Lee D.B., Bang J., Park B.-J., Wee C.-B., Kim D.J., Jang I.-S. (2017). Cordycepin induces human lung cancer cell apoptosis by inhibiting nitric oxide mediated ERK/Slug signaling pathway. Am. J. Cancer Res..

[89] Zhou X., Luo L., Dressel W., Shadier G., Krumbiegel D., Schmidtke P., Zepp F., Meyer C.U. (2008). Cordycepin is an immunoregulatory active ingredient of Cordyceps sinensis. Am. J. Chin. Med..

[90] Lichtman M.K., Otero-Vinas M., Falanga V. (2016). Transforming growth factor beta (TGF-β) isoforms in wound healing and fibrosis. Wound Repair Regen..

[91] Finnson K.W., Chi Y., Bou-Gharios G., Leask A., Philip A. (2012). TGF-β signaling in cartilage homeostasis and osteoarthritis. Front Biosci.

[92] Meng X.-m., Nikolic-Paterson D.J., Lan H.Y. (2016). TGF-β: The master regulator of fibrosis. Nat. Rev. Nephrol..

[93] Ma Z.-G., Yuan Y.-P., Wu H.-M., Zhang X., Tang Q.-Z. (2018). Cardiac fibrosis: New insights into the pathogenesis. Int. J. Biol. Sci..

[94] Chen M., Cheung F.W., Chan M.H., Hui P.K., Ip S.-P., Ling Y.H., Che C.-T., Liu W.K. (2012). Protective roles of Cordyceps on lung fibrosis in cellular and rat models. J. Ethnopharmacol..

[95] Gu L., Johno H., Nakajima S., Yoshitomi T., Takahashi S., Kitamura M. (2013). Intervention in Genotoxic Stress–Induced Senescence by Cordycepin Through Activation of eIF2α and Suppression of Sp1. Toxicol. Sci..

[96] Wang C.-W., Lee B.-H., Tai C.-J. (2017). The inhibition of cordycepin on cancer stemness in TGF-beta induced chemo-resistant ovarian cancer cell. Oncotarget.

[97] Ashraf S., Radhi M., Gowler P., Burston J.J., Gandhi R.D., Thorn G.J., Piccinini A.M., Walsh D.A., Chapman V., De Moor C.H. (2019). The polyadenylation inhibitor cordycepin reduces pain, inflammation and joint pathology in rodent models of osteoarthritis. Sci. Rep..

[98] Choi Y.H., Kim G.-Y., Lee H.H. (2014). Anti-inflammatory effects of cordycepin in lipopolysaccharide-stimulated RAW 264.7 macrophages through Toll-like receptor 4-mediated suppression of mitogen-activated protein kinases and NF-κB signaling pathways. Drug Des. Dev. Ther..

[99] Cui Z.Y., Park S.J., Jo E., Hwang I.-H., Lee K.-B., Kim S.-W., Kim D.J., Joo J.C., Hong S.H., Lee M.-G. (2018). Cordycepin induces apoptosis of human ovarian cancer cells by inhibiting CCL5-mediated Akt/NF-κB signaling pathway. Cell Death Discov..

[100] Jeong J.-W., Jin C.-Y., Kim G.-Y., Lee J.-D., Park C., Kim G.-D., Kim W.-J., Jung W.-K., Seo S.K., Choi I.-W. (2010). Anti-inflammatory effects of cordycepin via suppression of inflammatory mediators in BV2 microglial cells. Int. Immunopharmacol..

[101] Kim H.G., Shrestha B., Lim S.Y., Yoon D.H., Chang W.C., Shin D.-J., Han S.K., Park S.M., Park J.H., Park H.I. (2006). Cordycepin inhibits lipopolysaccharide-induced inflammation by the suppression of NF-κB through Akt and p38 inhibition in RAW 264.7 macrophage cells. Eur. J. Pharmacol..

[102] Peng J., Wang P., Ge H., Qu X., Jin X. (2015). Effects of cordycepin on the microglia-overactivation-induced impairments of growth and development of hippocampal cultured neurons. PLoS ONE.

[103] Yan L.J., Yang H.T., Duan H.Y., Wu J.T., Qian P., Fan X.W., Wang S. (2017). Cordycepin inhibits vascular adhesion molecule expression in TNF-α-stimulated vascular muscle cells. Exp. Ther. Med..

[104] Ying X., Peng L., Chen H., Shen Y., Yu K., Cheng S. (2014). Cordycepin prevented IL-β-induced expression of inflammatory mediators in human osteoarthritis chondrocytes. Int. Orthop..

[105] Guo Z., Chen W., Dai G., Huang Y. (2020). Cordycepin suppresses the migration and invasion of human liver cancer cells by downregulating the expression of CXCR4. Int. J. Mol. Med..

[106] Kondrashov A., Meijer H.A., Barthet-Barateig A., Parker H.N., Khurshid A., Tessier S., Sicard M., Knox A.J., Pang L., De Moor C.H. (2012). Inhibition of polyadenylation reduces inflammatory gene induction. Rna.

[107] Hwang I.-H., Oh S.Y., Jang H.-J., Jo E., Joo J.C., Lee K.-B., Yoo H.-S., Lee M.Y., Park S.J., Jang I.-S. (2017). Cordycepin promotes apoptosis in renal carcinoma cells by activating the MKK7-JNK signaling pathway through inhibition of c-FLIPL expression. PLoS ONE.

[108] Liang S.-M., Lu Y.-J., Ko B.-S., Jan Y.-J., Shyue S.-K., Yet S.-F., Liou J.-Y. (2017). Cordycepin disrupts leukemia association with mesenchymal stromal cells and eliminates leukemia stem cell activity. Sci. Rep..

[109] Sun T., Dong W., Jiang G., Yang J., Liu J., Zhao L., Ma P. (2019). Cordyceps militaris improves chronic kidney disease by affecting TLR4/NF-κB redox signaling pathway. Oxidative Med. Cell. Longev..

[110] Yang J., Zhou Y., Shi J. (2020). Cordycepin protects against acute pancreatitis by modulating NF-κB and NLRP3 inflammasome activation via AMPK. Life Sci..

[111] Sun J., Jin M., Zhou W., Diao S., Zhou Y., Li S., Wang X., Pan S., Jin X., Li G. (2017). A new ribonucleotide from Cordyceps militaris. Nat. Prod. Res..

[112] Kim J., Lee H., Kang K.S., Chun K.-H., Hwang G.S. (2015). Cordyceps militaris mushroom and cordycepin inhibit RANKL-induced osteoclast differentiation. J. Med. Food.

[113] Zhang J.L., Xu Y., Shen J. (2014). Cordycepin inhibits lipopolysaccharide (LPS)-induced tumor necrosis factor (TNF)-alpha production via activating amp-activated protein kinase (AMPK) signaling. Int. J. Mol. Sci..

[114] Baik J.-S., Kim K.-S., Moon H.-I., An H.-K., Park S.-J., Kim C.-H., Lee Y.-C. (2014). Cordycepin-mediated transcriptional regulation of human GD3 synthase (hST8Sia I) in human neuroblastoma SK-N-BE (2)-C cells. Acta Biochim Biophys Sin.

[115] Han M.W., Ryu I.S., Lee J.C., Kim S.H., Chang H.W., Lee Y.S., Lee S., Kim S.W., Kim S.Y. (2018). Phosphorylation of PI3K regulatory subunit p85 contributes to resistance against PI3K inhibitors in radioresistant head and neck cancer. Oral Oncol..

[116] Jia Y., Li H., Bao H., Zhang D., Feng L., Xiao Y., Zhu K., Hou Y., Luo S., Zhang Y. (2019). Cordycepin (3′-deoxyadenosine) promotes remyelination via suppression of neuroinflammation in a cuprizone-induced mouse model of demyelination. Int. Immunopharmacol..

[117] Kim H., Naura A.S., Errami Y., Ju J., Boulares A.H. (2011). Cordycepin blocks lung injury-associated inflammation and promotes BRCA1-deficient breast cancer cell killing by effectively inhibiting PARP. Mol. Med..

[118] Qing R., Huang Z., Tang Y., Xiang Q., Yang F. (2018). Cordycepin alleviates lipopolysaccharide-induced acute lung injury via Nrf2/HO-1 pathway. Int. Immunopharmacol..

[119] Tianzhu Z., Shihai Y., Juan D. (2014). Antidepressant-like effects of cordycepin in a mice model of chronic unpredictable mild stress. Evid. -Based Complementary Altern. Med..

[120] Wang M.-j., Peng X.-y., Lian Z.-q., Zhu H.-b. (2019). The cordycepin derivative IMM-H007 improves endothelial dysfunction by suppressing vascular inflammation and promoting AMPK-dependent eNOS activation in high-fat diet-fed ApoE knockout mice. Eur. J. Pharmacol..

[121] Won S.-Y., Park E.-H. (2005). Anti-inflammatory and related pharmacological activities of cultured mycelia and fruiting bodies of Cordyceps militaris. J. Ethnopharmacol..

[122] Okur M.H., Arslan S., Aydogdu B., Zeytun H., Basuguy E., Arslan M.S., Ibiloglu I., Kaplan I. (2018). Protective effect of cordycepin on experimental testicular ischemia/reperfusion injury in rats. J. Investig. Surg..

[123] Tianzhu Z., Shihai Y., Juan D. (2015). The effects of cordycepin on ovalbumin-induced allergic inflammation by strengthening Treg response and suppressing Th17 responses in ovalbumin-sensitized mice. Inflammation.

[124] Yang X., Li Y., He Y., Li T., Wang W., Zhang J., Wei J., Deng Y., Lin R. (2015). Cordycepin alleviates airway hyperreactivity in a murine model of asthma by attenuating the inflammatory process. Int. Immunopharmacol..

[125] Hung Y.-P., Lee C.-L. (2017). Higher anti-liver fibrosis effect of cordyceps militaris-fermented product cultured with deep ocean water via inhibiting proinflammatory factors and fibrosis-related factors expressions. Mar. Drugs.

[126] Zhang D.-w., Wang Z.-l., Qi W., Lei W., Zhao G.-y. (2014). Cordycepin (3′-deoxyadenosine) down-regulates the proinflammatory cytokines in inflammation-induced osteoporosis model. Inflammation.

[127] Li J., Zhong L., Zhu H., Wang F. (2017). The protective effect of cordycepin on D-galactosamine/lipopolysaccharide-induced acute liver injury. Mediat. Inflamm..

[128] Fei X., Zhang X., Zhang G.-q., Bao W.-p., Zhang Y.-y., Zhang M., Zhou X. (2017). Cordycepin inhibits airway remodeling in a rat model of chronic asthma. Biomed. Pharmacother..

[129] Lei J., Wei Y., Song P., Li Y., Zhang T., Feng Q., Xu G. (2018). Cordycepin inhibits LPS-induced acute lung injury by inhibiting inflammation and oxidative stress. Eur. J. Pharmacol..

[130] Rottenberg M.E., Masocha W., Ferella M., Petitto-Assis F., Goto H., Kristensson K., McCaffrey R., Wigzell H. (2005). Treatment of African trypanosomiasis with cordycepin and adenosine deaminase inhibitors in a mouse model. J. Infect. Dis..

[131] Wang X., Xi D., Mo J., Wang K., Luo Y., Xia E., Huang R., Luo S., Wei J., Ren Z. (2020). Cordycepin exhibits a suppressive effect on T cells through inhibiting TCR signaling cascade in CFA-induced inflammation mice model. Immunopharmacol. Immunotoxicol..

[132] Xiong Y., Zhang S., Xu L., Song B., Huang G., Lu J., Guan S. (2013). Suppression of T-cell activation in vitro and in vivo by cordycepin from Cordyceps militaris. J. Surg. Res..

[133] Yang R., Wang X., Xi D., Mo J., Wang K., Luo S., Wei J., Ren Z., Pang H., Luo Y. (2020). Cordycepin Attenuates IFN-γ-Induced Macrophage IP-10 and Mig Expressions by Inhibiting STAT1 Activity in CFA-Induced Inflammation Mice Model. Inflammation.

[134] Zeng Y., Lian S., Li D., Lin X., Chen B., Wei H., Yang T. (2017). Anti-hepatocarcinoma effect of cordycepin against NDEA-induced hepatocellular carcinomas via the PI3K/Akt/mTOR and Nrf2/HO-1/NF-κB pathway in mice. Biomed. Pharmacother..

[135] Gong X., Li T., Wan R., Sha L. (2021). Cordycepin attenuates high-fat diet-induced non-alcoholic fatty liver disease via down-regulation of lipid metabolism and inflammatory responses. Int. Immunopharmacol..

[136] Chamcheu J.C., Roy T., Uddin M.B., Banang-Mbeumi S., Chamcheu R.-C.N., Walker A.L., Liu Y.-Y., Huang S. (2019). Role and therapeutic targeting of the PI3K/Akt/mTOR signaling pathway in skin cancer: A review of current status and future trends on natural and synthetic agents therapy. Cells.

[137] Gong J., Zhang L., Zhang Q., Li X., Xia X.-J., Liu Y.-Y., Yang Q.-S. (2018). Lentiviral vector-mediated SHC3 silencing exacerbates oxidative stress injury in nigral dopamine neurons by regulating the PI3K-AKT-FoxO signaling pathway in rats with Parkinson’s disease. Cell. Physiol. Biochem..

[138] Huang X., Liu G., Guo J., Su Z. (2018). The PI3K/AKT pathway in obesity and type 2 diabetes. Int. J. Biol. Sci..

[139] Riquelme I., Tapia O., Espinoza J.A., Leal P., Buchegger K., Sandoval A., Bizama C., Araya J.C., Peek R.M., Roa J.C. (2016). The gene expression status of the PI3K/AKT/mTOR pathway in gastric cancer tissues and cell lines. Pathol. Oncol. Res..

[140] Smith H.J., Sharma A., Mair W.B. (2020). Metabolic Communication and Healthy Aging: Where Should We Focus Our Energy?. Dev. Cell.

[141] Chiang G.G., Abraham R.T. (2005). Phosphorylation of mammalian target of rapamycin (mTOR) at Ser-2448 is mediated by p70S6 kinase. J Biol Chem.

[142] Bi Y., Li H., Yi D., Sun Y., Bai Y., Zhong S., Song Y., Zhao G., Chen Y. (2018). Cordycepin augments the chemosensitivity of human glioma cells to temozolomide by activating AMPK and inhibiting the AKT signaling pathway. Mol. Pharm..

[143] Hsu P.-Y., Lin Y.-H., Yeh E.-L., Lo H.-C., Hsu T.-H., Su C.-C. (2017). Cordycepin and a preparation from Cordyceps militaris inhibit malignant transformation and proliferation by decreasing EGFR and IL-17RA signaling in a murine oral cancer model. Oncotarget.

[144] Takahashi S., Tamai M., Nakajima S., Kato H., Johno H., Nakamura T., Kitamura M. (2012). Blockade of adipocyte differentiation by cordycepin. Br. J. Pharmacol..

[145] Wang Z., Chen Z., Jiang Z., Luo P., Liu L., Huang Y., Wang H., Wang Y., Long L., Tan X. (2019). Cordycepin prevents radiation ulcer by inhibiting cell senescence via NRF2 and AMPK in rodents. Nat. Commun..

[146] Wu W.-D., Hu Z.-M., Shang M.-J., Zhao D.-J., Zhang C.-W., Hong D.-F., Huang D.-S. (2014). Cordycepin down-regulates multiple drug resistant (MDR)/HIF-1α through regulating AMPK/mTORC1 signaling in GBC-SD gallbladder cancer cells. Int. J. Mol. Sci..

[147] Kumar C.C., Madison V. (2005). AKT crystal structure and AKT-specific inhibitors. Oncogene.

[148] Wei Y., Zhou J., Yu H., Jin X. (2019). AKT phosphorylation sites of Ser473 and Thr308 regulate AKT degradation. Biosci Biotechnol Biochem.

[149] Ko B.S., Lu Y.J., Yao W.L., Liu T.A., Tzean S.S., Shen T.L., Liou J.Y. (2013). Cordycepin regulates GSK-3beta/beta-catenin signaling in human leukemia cells. PLoS ONE.

[150] Wang Y., Mo H., Gu J., Chen K., Han Z., Liu Y. (2017). Cordycepin induces apoptosis of human acute monocytic leukemia cells via downregulation of the ERK/Akt signaling pathway. Exp. Ther. Med..

[151] Wang Z., Wu X., Liang Y.-N., Wang L., Song Z.-X., Liu J.-L., Tang Z.-S. (2016). Cordycepin induces apoptosis and inhibits proliferation of human lung cancer cell line H1975 via inhibiting the phosphorylation of EGFR. Molecules.

[152] Wong Y.Y., Moon A., Duffin R., Barthet-Barateig A., Meijer H.A., Clemens M.J., de Moor C.H. (2010). Cordycepin inhibits protein synthesis and cell adhesion through effects on signal transduction. J. Biol. Chem..

[153] Yang C., Zhao L., Yuan W., Wen J. (2017). Cordycepin induces apoptotic cell death and inhibits cell migration in renal cell carcinoma via regulation of microRNA-21 and PTEN phosphatase. Biomed. Res..

[154] Oh S.-S., Lee K.W., Madhi H., Jeong J.-W., Park S., Kim M., Lee Y., Han H.-T., Hwangbo C., Yoo J. (2020). Cordycepin Resensitizes T24R2 Cisplatin-Resistant Human Bladder Cancer Cells to Cisplatin by Inactivating Ets-1 Dependent MDR1 Transcription. Int. J. Mol. Sci..

[155] Cao H.-L., Liu Z.-J., Chang Z. (2017). Cordycepin induces apoptosis in human bladder cancer cells via activation of A3 adenosine receptors. Tumor Biol..

[156] Jeon S.-M. (2016). Regulation and function of AMPK in physiology and diseases. Exp. Mol. Med..

[157] Wong K.A., Lodish H.F. (2006). A revised model for AMP-activated protein kinase structure: The alpha-subunit binds to both the beta- and gamma-subunits although there is no direct binding between the beta- and gamma-subunits. J Biol Chem.

[158] Hardie D.G. (2011). AMP-activated protein kinase—An energy sensor that regulates all aspects of cell function. Genes Dev..

[159] Dasgupta B., Chhipa R.R. (2016). Evolving lessons on the complex role of AMPK in normal physiology and cancer. Trends Pharmacol. Sci..

[160] Gauthier M.-S., O’Brien E.L., Bigornia S., Mott M., Cacicedo J.M., Xu X.J., Gokce N., Apovian C., Ruderman N. (2011). Decreased AMP-activated protein kinase activity is associated with increased inflammation in visceral adipose tissue and with whole-body insulin resistance in morbidly obese humans. Biochem. Biophys. Res. Commun..

[161] Valentine R.J., Coughlan K.A., Ruderman N.B., Saha A.K. (2014). Insulin inhibits AMPK activity and phosphorylates AMPK Ser485/491 through Akt in hepatocytes, myotubes and incubated rat skeletal muscle. Arch. Biochem. Biophys..

[162] Willows R., Sanders M.J., Xiao B., Patel B.R., Martin S.R., Read J., Wilson J.R., Hubbard J., Gamblin S.J., Carling D. (2017). Phosphorylation of AMPK by upstream kinases is required for activity in mammalian cells. Biochem J.

[163] Dite T.A., Ling N.X., Scott J.W., Hoque A., Galic S., Parker B.L., Ngoei K.R., Langendorf C.G., O’Brien M.T., Kundu M. (2017). The autophagy initiator ULK1 sensitizes AMPK to allosteric drugs. Nat. Commun..

[164] Guo P., Kai Q., Gao J., Lian Z.Q., Wu C.M., Wu C.A., Zhu H.B. (2010). Cordycepin prevents hyperlipidemia in hamsters fed a high-fat diet via activation of AMP-activated protein kinase. J Pharm. Sci.

[165] Hawley S.A., Ross F.A., Russell F.M., Atrih A., Lamont D.J., Hardie D.G. (2020). Mechanism of Activation of AMPK by Cordycepin. Cell Chem. Biol..

[166] Qi G., Zhou Y., Zhang X., Yu J., Li X., Cao X., Wu C., Guo P. (2019). Cordycepin promotes browning of white adipose tissue through an AMP-activated protein kinase (AMPK)-dependent pathway. Acta Pharm. Sin. B.

[167] Wu C., Guo Y., Su Y., Zhang X., Luan H., Zhang X., Zhu H., He H., Wang X., Sun G. (2014). Cordycepin activates AMP-activated protein kinase (AMPK) via interaction with the γ1 subunit. J. Cell. Mol. Med..

[168] Lee C., Kim Y., Jeon J.H. (2016). JNK and p38 mitogen-activated protein kinase pathways contribute to porcine epidemic diarrhea virus infection. Virus Res..

[169] Ko H.M., Joo S.H., Kim P., Park J.H., Kim H.J., Bahn G.H., Kim H.Y., Lee J., Han S.-H., Shin C.Y. (2013). Effects of Korean Red Ginseng extract on tissue plasminogen activator and plasminogen activator inhibitor-1 expression in cultured rat primary astrocytes. J. Ginseng Res..

[170] Koul H.K., Pal M., Koul S. (2013). Role of p38 MAP kinase signal transduction in solid tumors. Genes Cancer.

[171] Lim A.K., Tesch G.H. (2012). Inflammation in diabetic nephropathy. Mediat. Inflamm..

[172] Ren F., Zhang H., Piao Z., Zheng S., Chen Y., Chen D., Duan Z. (2012). Inhibition of glycogen synthase kinase 3b activity regulates Toll-like receptor 4-mediated liver inflammation. Zhonghua Gan Zang Bing Za Zhi = Zhonghua Ganzangbing Zazhi = Chin. J. Hepatol..

[173] Cuenda A., Rousseau S. (2007). p38 MAP-kinases pathway regulation, function and role in human diseases. Biochim. Et Biophys. Acta (Bba)-Mol. Cell Res..

[174] Imesch P., Hornung R., Fink D., Fedier A. (2011). Cordycepin (3′-deoxyadenosine), an inhibitor of mRNA polyadenylation, suppresses proliferation and activates apoptosis in human epithelial endometriotic cells in vitro. Gynecol. Obstet. Investig..

[175] Du Y., Yu J., Du L., Tang J., Feng W.-H. (2016). Cordycepin enhances Epstein–Barr virus lytic infection and Epstein–Barr virus-positive tumor treatment efficacy by doxorubicin. Cancer Lett..

[176] Lee S.-J., Kim S.-K., Choi W.-S., Kim W.-J., Moon S.-K. (2009). Cordycepin causes p21WAF1-mediated G2/M cell-cycle arrest by regulating c-Jun N-terminal kinase activation in human bladder cancer cells. Arch. Biochem. Biophys..

[177] Pao H.-Y., Pan B.-S., Leu S.-F., Huang B.-M. (2012). Cordycepin stimulated steroidogenesis in MA-10 mouse Leydig tumor cells through the protein kinase C Pathway. J. Agric. Food Chem..

[178] Liao Y., Ling J., Zhang G., Liu F., Tao S., Han Z., Chen S., Chen Z., Le H. (2015). Cordycepin induces cell cycle arrest and apoptosis by inducing DNA damage and up-regulation of p53 in Leukemia cells. Cell Cycle.

[179] Yarza R., Vela S., Solas M., Ramirez M.J. (2016). c-Jun N-terminal kinase (JNK) signaling as a therapeutic target for Alzheimer’s disease. Front. Pharmacol..

[180] Zeke A., Misheva M., Reményi A., Bogoyevitch M.A. (2016). JNK signaling: Regulation and functions based on complex protein-protein partnerships. Microbiol. Mol. Biol. Rev..

[181] Guo Y.J., Pan W.W., Liu S.B., Shen Z.F., Xu Y., Hu L.L. (2020). ERK/MAPK signalling pathway and tumorigenesis. Exp. Ther. Med..

[182] Kraus I., Besong Agbo D., Otto M., Wiltfang J., Klafki H. (2015). Detection and Differentiation of Threonine- and Tyrosine-Monophosphorylated Forms of ERK1/2 by Capillary Isoelectric Focusing-Immunoassay. Sci. Rep..

[183] Park E.-S., Kang D.-H., Yang M.-K., Kang J.C., Jang Y.C., Park J.S., Kim S.-K., Shin H.-S. (2014). Cordycepin, 3′-deoxyadenosine, prevents rat hearts from ischemia/reperfusion injury via activation of Akt/GSK-3β/p70S6K signaling pathway and HO-1 expression. Cardiovasc. Toxicol..

[184] Wang H.B., Duan M.X., Xu M., Huang S.H., Yang J., Yang J., Liu L.B., Huang R., Wan C.X., Ma Z.G. (2019). Cordycepin ameliorates cardiac hypertrophy via activating the AMPKalpha pathway. J. Cell Mol. Med..

[185] Jagger D.V., Kredich N.M., Guarino A.J. (1961). Inhibition of Ehrlich mouse ascites tumor growth by cordycepin. Cancer Res..

[186] Bi Y., Li H., Yi D., Bai Y., Zhong S., Liu Q., Chen Y., Zhao G. (2018). β-catenin contributes to cordycepin-induced MGMT inhibition and reduction of temozolomide resistance in glioma cells by increasing intracellular reactive oxygen species. Cancer Lett..

[187] Dong J., Li Y., Xiao H., Luo D., Zhang S., Zhu C., Jiang M., Cui M., Lu L., Fan S. (2019). Cordycepin sensitizes breast cancer cells toward irradiation through elevating ROS production involving Nrf2. Toxicol. Appl. Pharmacol..

[188] Horsman M.R., Brown D.M., Hirst D.G., Brown J.M. (1986). The effects of purine nucleoside analogs on the response of the RIF-1 tumor to melphalan in vivo. Int. J. Radiat. Oncol. Biol. Phys..

[189] Wang C., Mao Z., Wang L., Zhang F., Wu G., Wang D., Shi J. (2017). Cordycepin inhibits cell growth and induces apoptosis in human cholangiocarcinoma. Neoplasma.

[190] Wu J.Y., Zhang Q.X., Leung P.H. (2007). Inhibitory effects of ethyl acetate extract of Cordyceps sinensis mycelium on various cancer cells in culture and B16 melanoma in C57BL/6 mice. Phytomedicine.

[191] Yoshikawa N., Nakamura K., Yamaguchi Y., Kagota S., Shinozuka K., Kunitomo M. (2004). Antitumour activity of cordycepin in mice. Clin. Exp. Pharmacol. Physiol..

[192] Zhou Y., Guo Z., Meng Q., Lu J., Wang N., Liu H., Liang Q., Quan Y., Wang D., Xie J. (2017). Cordycepin affects multiple apoptotic pathways to mediate hepatocellular carcinoma cell death. Anti-Cancer Agents Med. Chem. (Former. Curr. Med. Chem. -Anti-Cancer Agents).

[193] Aramwit P., Porasuphatana S., Srichana T., Nakpheng T. (2015). Toxicity evaluation of cordycepin and its delivery system for sustained in vitro anti-lung cancer activity. Nanoscale Res. Lett..

[194] Foss F.M. (2000). Combination therapy with purine nucleoside analogs. Oncol. (Williston ParkNy).

[195] Johns D.G., Adamson R.H. (1976). Enhancement of the biological activity of cordycepin (3’-deoxyadenosine) by the adenosine deaminase inhibitor 2’-deoxycoformycin. Biochem. Pharmacol..

[196] Joo J.C., Hwang J.H., Jo E., Kim Y.-R., Kim D.J., Lee K.-B., Park S.J., Jang I.-S. (2017). Cordycepin induces apoptosis by caveolin-1-mediated JNK regulation of Foxo3a in human lung adenocarcinoma. Oncotarget.

[197] Sato A., Yoshikawa N., Kubo E., Kakuda M., Nishiuchi A., Kimoto Y., Takahashi Y., Kagota S., Shinozuka K., Nakamura K. (2013). Inhibitory effect of cordycepin on experimental hepatic metastasis of B16-F0 mouse melanoma cells. Vivo.

[198] Wu P.-K., Tao Z., Ouyang Z., Cao J.-Y., Geng D., Liu J., Wang C.-M. (2016). The anti-tumor effects of cordycepin-loaded liposomes on the growth of hepatoma 22 tumors in mice and human hepatoma BEL-7402 cells in culture. Drug Dev. Ind. Pharm..

[199] Yoshikawa N., Kunitomo M., Kagota S., Shinozuka K., Nakamura K. (2009). Inhibitory effect of cordycepin on hematogenic metastasis of B16-F1 mouse melanoma cells accelerated by adenosine-5′-diphosphate. Anticancer Res..

[200] Liu T., Zhu G., Yan W., Lv Y., Wang X., Jin G., Cui M., Lin Z., Ren X. (2020). Cordycepin inhibits cancer cell proliferation and angiogenesis through a DEK interaction via ERK signaling in cholangiocarcinoma. J. Pharmacol. Exp. Ther..

[201] Xue-Ying L., Homng T., Can J., Zhen-Yun D., Wen-Feng L., Qing-Jiu T., Kan D. (2020). Cordycepin inhibits pancreatic cancer cell growth in vitro and in vivo via targeting FGFR2 and blocking ERK signaling. Chin. J. Nat. Med..

[202] Zhang Y., Zhang X.X., Yuan R.Y., Ren T., Shao Z.Y., Wang H.F., Cai W.L., Chen L.T., Wang X.A., Wang P. (2018). Cordycepin induces apoptosis in human pancreatic cancer cells via the mitochondrial-mediated intrinsic pathway and suppresses tumor growth in vivo. Oncotargets Ther..

[203] Zheng Q., Sun J., Li W., Li S., Zhang K. (2020). Cordycepin induces apoptosis in human tongue cancer cells in vitro and has antitumor effects in vivo. Arch. Oral Biol..

[204] Cao Z.-P., Shang Y.-J., Liu Q.-Y., Wu B.-Y., Liu W.-X., Li C.-H. (2019). Neuroprotection of cordycepin in NMDA-induced excitotoxicity by modulating adenosine A1 receptors. Eur. J. Pharmacol..

[205] Cheng Z., He W., Zhou X., Lv Q., Xu X., Yang S., Zhao C., Guo L. (2011). Cordycepin protects against cerebral ischemia/reperfusion injury in vivo and in vitro. Eur. J. Pharmacol..

[206] Hwang I.K., Lim S.S., Yoo K.-Y., Lee Y.S., Kim H.G., Kang I.-J., Kwon H.J., Park J., Choi S.Y., Won M.-H. (2008). A phytochemically characterized extract of Cordyceps militaris and cordycepin protect hippocampal neurons from ischemic injury in gerbils. Planta Med..

[207] Gao J., Lian Z., Zhu P., Zhu H. (2011). Lipid-lowering effect of cordycepin (3’-deoxyadenosine) from Cordyceps militaris on hyperlipidemic hamsters and rats. Yao Xue Xue Bao= Acta Pharm. Sin..

[208] Sun Y., Wang Y.-H., Qu K., Zhu H.-B. (2011). Beneficial effects of cordycepin on metabolic profiles of liver and plasma from hyperlipidemic hamsters. J. Asian Nat. Prod. Res..

[209] Cheng Y., Wei Y., Yang W., Song Y., Shang H., Cai Y., Wu Z., Zhao W. (2017). Cordycepin confers neuroprotection in mice models of intracerebral hemorrhage via suppressing NLRP3 inflammasome activation. Metab. Brain Dis..

[210] Won K.-J., Lee S.-C., Lee C.-K., Lee H.M., Lee S.H., Fang Z., Choi O.B., Jin M., Kim J., Park T. (2009). Cordycepin attenuates neointimal formation by inhibiting reactive oxygen species–mediated responses in vascular smooth muscle cells in rats. J. Pharmacol. Sci..

[211] Xingqiang L., Fen N., Zhongpeng Y., Tiantian W., Lei Z., Jiali F., Junjie M., Guanghui L., Lu X., Yuhe G. (2019). Ethylene carbodiimide-fixed donor splenocytes combined with cordycepin induce long-term protection to mice cardiac allografts. Transpl. Immunol..

[212] Araldi D., Ferrari L.F., Levine J.D. (2016). Gi-protein coupled 5-HT1B/D receptor agonist sumatriptan induces type I hyperalgesic priming. Pain.

[213] Araldi D., Ferrari L.F., Levine J.D. (2018). Mu-opioid receptor (MOR) biased agonists induce biphasic dose-dependent hyperalgesia and analgesia, and hyperalgesic priming in the rat. Neuroscience.

[214] Ferrari L.F., Araldi D., Bogen O., Green P.G., Levine J.D. (2019). Systemic morphine produces dose-dependent nociceptor-mediated biphasic changes in nociceptive threshold and neuroplasticity. Neuroscience.

[215] Ferrari L.F., Araldi D., Levine J.D. (2015). Distinct terminal and cell body mechanisms in the nociceptor mediate hyperalgesic priming. J. Neurosci..

[216] Ferrari L.F., Bogen O., Chu C., Levine J.D. (2013). Peripheral administration of translation inhibitors reverses increased hyperalgesia in a model of chronic pain in the rat. J. Pain.

[217] Khomula E.V., Araldi D., Levine J.D. (2019). In vitro nociceptor neuroplasticity associated with in vivo opioid-induced hyperalgesia. J. Neurosci..

[218] Song R., Zheng J., Liu Y., Tan Y., Yang Z., Song X., Yang S., Fan R., Zhang Y., Wang Y. (2019). A natural cordycepin/chitosan complex hydrogel with outstanding self-healable and wound healing properties. Int. J. Biol. Macromol..

[219] Yang F., Sun W., Luo W.-J., Yang Y., Yang F., Wang X.-L., Chen J. (2017). SDF1-CXCR4 signaling contributes to the transition from acute to chronic pain state. Mol. Neurobiol..

[220] Aiyedun B., Williamson J., Amodu A. (1973). The effect of cordy-cepin on tsetse-borne Trypanosoma vivax infections. Acta Trop..

[221] Da Silva A.S., Wolkmer P., Nunes J.T., Duck M.R., Oliveira C.B., Gressler L.T., Costa M.M., Zanette R.A., Mazzanti C.M., Lopes S.T. (2011). Susceptibility of Trypanosoma evansi to cordycepin. Biomed. Pharmacother..

[222] Dalla Rosa L., Da Silva A.S., Oliveira C.B., Gressler L.T., Arnold C.B., Baldissera M.D., Sagrillo M., Sangoi M., Moresco R., Mendes R.E. (2015). Dose finding of 3′ deoxyadenosine and deoxycoformycin for the treatment of Trypanosoma evansi infection: An effective and nontoxic dose. Microb. Pathog..

[223] Dalla Rosa L., Da Silva A.S., Ruchel J.B., Gressler L.T., Oliveira C.B., França R.T., Lopes S.T., Leal D.B., Monteiro S.G. (2013). Influence of treatment with 3′-deoxyadenosine associated deoxycoformycin on hematological parameters and activity of adenosine deaminase in infected mice with Trypanosoma evansi. Exp. Parasitol..

[224] Dalla Rosa L., Da Silva A.S., Gressler L.T., Oliveira C.B., Dambros M.G., Miletti L.C., T. FRANÇA R., Lopes S.T., Samara Y.N., Da Veiga M.L. (2013). Cordycepin (3’-deoxyadenosine) pentostatin (deoxycoformycin) combination treatment of mice experimentally infected with Trypanosoma evansi. Parasitology.

[225] do Carmo G.M., de Sá M.F., Grando T.H., Gressler L.T., Baldissera M.D., Monteiro S.G., Henker L.C., Mendes R.E., Stefani L.M., Da Silva A.S. (2019). Cordycepin (3′-deoxyadenosine) and pentostatin (deoxycoformycin) against Trypanosoma cruzi. Exp. Parasitol..

[226] do Carmo G.M., Doleski P.H., de Sá M.F., Grando T.H., Azevedo M.I., Manzoni A.G., Leal D.B., Gressler L.T., Henker L.C., Mendes R.E. (2017). Treatment with 3′-deoxyadenosine and deoxycoformycin in mice infected by Trypanosoma cruzi and its side effect on purinergic enzymes. Microb. Pathog..

[227] Vodnala S.K., Ferella M., Lundén-Miguel H., Betha E., Van Reet N., Amin D.N., Öberg B., Andersson B., Kristensson K., Wigzell H. (2009). Preclinical assessment of the treatment of second-stage African trypanosomiasis with cordycepin and deoxycoformycin. PLoS Negl. Trop. Dis..

[228] Vodnala S.K., Lundbäck T., Yeheskieli E., Sjöberg B., Gustavsson A.-L., Svensson R., Olivera G.C., Eze A.A., de Koning H.P., Hammarström L.G. (2013). Structure–activity relationships of synthetic cordycepin analogues as experimental therapeutics for African trypanosomiasis. J. Med. Chem..

[229] Williamson J. (1972). Further experiments with the nucleoside trypanocide, cordycepin. Trans. R. Soc. Trop. Med. Hyg..

[230] Williamson J., Macadam R. (1976). Drug effects on the fine structure of Trypanosoma rhodesiense: Puromycin and its aminonucleoside, Cordycepin and Nucleocidin. Trans. R. Soc. Trop. Med. Hyg..

[231] Williamson J., Scott-Finnigan T. (1978). Trypanocidal activity of antitumor antibiotics and other metabolic inhibitors. Antimicrob. Agents Chemother..

[232] Li B., Hou Y., Zhu M., Bao H., Nie J., Zhang G.Y., Shan L., Yao Y., Du K., Yang H. (2016). 3’-deoxyadenosine (cordycepin) produces a rapid and robust antidepressant effect via enhancing prefrontal AMPA receptor signaling pathway. Int. J. Neuropsychopharmacol..

[233] Cai Z.-L., Wang C.-Y., Jiang Z.-J., Li H.-H., Liu W.-X., Gong L.-W., Xiao P., Li C.-H. (2013). Effects of cordycepin on Y-maze learning task in mice. Eur. J. Pharmacol..

[234] Cao Z.-P., Dai D., Wei P.-J., Han Y.-Y., Guan Y.-Q., Li H.-H., Liu W.-X., Xiao P., Li C.-H. (2018). Effects of cordycepin on spontaneous alternation behavior and adenosine receptors expression in hippocampus. Physiol. Behav..

[235] Han Y.Y., Chen Z.H., Shang Y.J., Yan W.W., Wu B.Y., Li C.H. (2019). Cordycepin improves behavioral-LTP and dendritic structure in hippocampal CA1 area of rats. J. Neurochem..

[236] Zearfoss N.R., Alarcon J.M., Trifilieff P., Kandel E., Richter J.D. (2008). A molecular circuit composed of CPEB-1 and c-Jun controls growth hormone-mediated synaptic plasticity in the mouse hippocampus. J. Neurosci..

[237] Hu Z., Lee C.-I., Shah V.K., Oh E.-H., Han J.-Y., Bae J.-R., Lee K., Chong M.-S., Hong J.T., Oh K.-W. (2013). Cordycepin increases nonrapid eye movement sleep via adenosine receptors in rats. Evid. -Based Complementary Altern. Med..

[238] An Y., Li Y., Wang X., Chen Z., Xu H., Wu L., Li S., Wang C., Luan W., Wang X. (2018). Cordycepin reduces weight through regulating gut microbiota in high-fat diet-induced obese rats. Lipids Health Dis..

[239] Li Y., Li Y., Wang X., Xu H., Wang C., An Y., Luan W., Wang X., Li S., Ma F. (2018). Cordycepin modulates body weight by reducing prolactin via an adenosine A1 receptor. Curr. Pharm. Des..

[240] Xu H., Wu B., Wang X., Ma F., Li Y., An Y., Wang C., Wang X., Luan W., Li S. (2019). Cordycepin regulates body weight by inhibiting lipid droplet formation, promoting lipolysis and recruiting beige adipocytes. J. Pharm. Pharmacol..

[241] Cao T., Xu R., Xu Y., Liu Y., Qi D., Wan Q. (2019). The protective effect of Cordycepin on diabetic nephropathy through autophagy induction in vivo and in vitro. Int. Urol. Nephrol..

[242] Cimbala M., Lamers W., Nelson K., Monahan J., Yoo-Warren H., Hanson R. (1982). Rapid changes in the concentration of phosphoenolpyruvate carboxykinase mRNA in rat liver and kidney. Effects of insulin and cyclic AMP. J. Biol. Chem..

[243] Ma L., Zhang S., Du M. (2015). Cordycepin from Cordyceps militaris prevents hyperglycemia in alloxan-induced diabetic mice. Nutr. Res..

[244] Borgan J.-L., Bonnafous J.-C., Mousseron-Canet M., Mani J.-C., Cazaubon C. (1976). Cordycepin and early effects of estradiol on the immature rat uterus. Biochimie.

[245] Fernandez-Noval A., Leroy F. (1979). 3′-DEOXYADENOSINE AND IMPLANTATION OF DELAYED BLASTOCYSTS IN MICE. J. Endocrinol..

[246] Leu S.-F., Poon S.L., Pao H.-Y., Huang B.-M. (2011). The in vivo and in vitro stimulatory effects of cordycepin on mouse leydig cell steroidogenesis. Biosci. Biotechnol. Biochem..

[247] Lin W.-H., Tsai M.-T., Chen Y.-S., Hou R.C.-W., Hung H.-F., Li C.-H., Wang H.-K., Lai M.-N., Jeng K.-C.G. (2007). Improvement of sperm production in subfertile boars by Cordyceps militaris supplement. Am. J. Chin. Med..

[248] Sohn S.-H., Lee S.-C., Hwang S.-Y., Kim S.-W., Kim I.-W., Michael B.Y., Kim S.-K. (2012). Effect of long-term administration of cordycepin from Cordyceps militaris on testicular function in middle-aged rats. Planta Med..

[249] Ulibarri C., Yahr P. (1987). Poly-A+ mRNA and defeminization of sexual behavior and gonadotropin secretion in rats. Physiol. Behav..

[250] Yahr P., Ulibarri C. (1986). Estrogen induction of sexual behavior in female rats and synthesis of polyadenylated messenger RNA in the ventromedial nucleus of the hypothalamus. Mol. Brain Res..

[251] Yahr P., Ulibarri C. (1987). Polyadenylated and nonadenylated messenger RNA and androgen control of sexual behavior and scent marking in male gerbils. Horm. Behav..

[252] Dickson B.M., Roelofs A.J., Rochford J.J., Wilson H.M., De Bari C. (2019). The burden of metabolic syndrome on osteoarthritic joints. Arthritis Res. Ther..

[253] Kang S., Kumanogoh A. (2020). The spectrum of macrophage activation by immunometabolism. Int. Immunol..

[254] Noe J.T., Mitchell R.A. (2019). Tricarboxylic acid cycle metabolites in the control of macrophage activation and effector phenotypes. J. Leukoc. Biol..

[255] O’Neill L.A., Pearce E.J. (2016). Immunometabolism governs dendritic cell and macrophage function. J. Exp. Med..

[256] Marslin G., Khandelwal V., Franklin G. (2020). Cordycepin nanoencapsulated in poly (Lactic-Co-Glycolic acid) exhibits better cytotoxicity and lower hemotoxicity than free drug. Nanotechnol. Sci. Appl..

[257] Lee J.B., Adrower C., Qin C., Fischer P.M., de Moor C.H., Gershkovich P. (2017). Development of cordycepin formulations for preclinical and clinical studies. Aaps Pharmscitech.

[258] Zhang J.-Q., Wu D., Jiang K.-M., Zhang D., Zheng X., Wan C.-P., Zhu H.-Y., Xie X.-G., Jin Y., Lin J. (2015). Preparation, spectroscopy and molecular modelling studies of the inclusion complex of cordycepin with cyclodextrins. Carbohydr. Res..

[259] Bi Y., Zhou Y., Wang M., Li L., Lee R.J., Xie J., Teng L. (2017). Targeted delivery of cordycepin to liver cancer cells using transferrin-conjugated liposomes. Anticancer Res..

[260] Cheek M.A., Dobrikov M.I., Wennefors C.K., Xu Z., Hashmi S.N., Shen X., Shaw B.R. (2008). Synthesis and properties of (α-P-borano)-nucleoside 5′-triphosphate analogues as potential antiviral agents. Nucleic Acids Symp. Ser..

[261] Hulpia F., Mabille D., Campagnaro G.D., Schumann G., Maes L., Roditi I., Hofer A., De Koning H.P., Caljon G., Van Calenbergh S. (2019). Combining tubercidin and cordycepin scaffolds results in highly active candidates to treat late-stage sleeping sickness. Nat. Commun..

[262] Miao Z.-X., Yang L., Jiang C.-Y., Wang Y.-H., Zhu H.-B. (2014). Evaluation of dose-related effects of 2’, 3’, 5’-tri-O-acetyl-N6-(3-hydroxylaniline) adenosine using NMR-based metabolomics. Yao Xue Xue Bao= Acta Pharm. Sin..

[263] Kubota Y., Ehara M., Haraguchi K., Tanaka H. (2011). Phenylsulfanylation of 3′, 4′-Unsaturated Adenosine Employing Thiophenol-N-Iodosuccinimide Leads to 4′-Phenylsulfanylcordycepin: Synthesis of 4′-Substituted Cordycepins on the Basis of Substitution of the Phenylsulfanyl Leaving Group. J. Org. Chem..

[264] Fong P., Ao C.N., Tou K.I., Huang K.M., Cheong C.C., Meng L.R. (2019). Experimental and in silico analysis of cordycepin and its derivatives as endometrial cancer treatment. Oncol. Res..

[265] Chaicharoenaudomrung N., Kunhorm P., Promjantuek W., Heebkaew N., Rujanapun N., Noisa P. (2019). Fabrication of 3D calcium-alginate scaffolds for human glioblastoma modeling and anticancer drug response evaluation. J. Cell. Physiol..

[266] El Khadem H.S., El Sayed H. (1974). Synthesis of a c-nucleoside analog of the antibiotic cordycepin. Carbohydr. Res..

[267] Kaokaen P., Jaiboonma A., Chaicharoenaudomrung N., Kunhorm P., Janebodin K., Noisa P., Jitprasertwong P. (2020). Cordycepin-loaded Nanoparticles from Cassava Starch Promote the Proliferation of Submandibular Gland Cells and Inhibit the Growth of Oral Squamous Carcinoma Cells. Nutr. Cancer.

[268] Holbein S., Wengi A., Decourty L., Freimoser F.M., Jacquier A., Dichtl B. (2009). Cordycepin interferes with 3′ end formation in yeast independently of its potential to terminate RNA chain elongation. RNA.

[269] Wang Z., Wang X., Qu K., Zhu P., Guo N., Zhang R., Abliz Z., Yu H., Zhu H. (2010). Binding of Cordycepin Monophosphate to AMP-Activated Protein Kinase and its Effect on AMP-Activated Protein Kinase Activation. Chem. Biol. Drug Des..

[270] Yoon J.Y., Kim J.H., Baek K.-S., Kim G.S., Lee S.E., Lee D.Y., Choi J.H., Kim S.Y., Park H.B., Sung G.-H. (2015). A direct protein kinase B-targeted anti-inflammatory activity of cordycepin from artificially cultured fruit body of Cordyceps militaris. Pharmacogn. Mag..

[271] Bard J., Zhelkovsky A.M., Helmling S., Earnest T.N., Moore C.L., Bohm A. (2000). Structure of yeast poly (A) polymerase alone and in complex with 3’-dATP. Science.

[272] Ryner L.C., Manley J.L. (1987). Requirements for accurate and efficient mRNA 3’end cleavage and polyadenylation of a simian virus 40 early pre-RNA in vitro. Mol. Cell. Biol..

[273] Ju D., Zhang W., Yan J., Zhao H., Li W., Wang J., Liao M., Xu Z., Wang Z., Zhou G. (2020). Chemical perturbations reveal that RUVBL2 regulates the circadian phase in mammals. Sci. Transl. Med..

[274] Kadomatsu M., Nakajima S., Kato H., Gu L., Chi Y., Yao J., Kitamura M. (2012). Cordycepin as a sensitizer to tumour necrosis factor (TNF)-α-induced apoptosis through eukaryotic translation initiation factor 2α (eIF2α)-and mammalian target of rapamycin complex 1 (mTORC1)-mediated inhibition of nuclear factor (NF)-κB. Clin. Exp. Immunol..

[275] Yoshikawa N., Yamada S., Takeuchi C., Kagota S., Shinozuka K., Kunitomo M., Nakamura K. (2008). Cordycepin (3′-deoxyadenosine) inhibits the growth of B16-BL6 mouse melanoma cells through the stimulation of adenosine A 3 receptor followed by glycogen synthase kinase-3β activation and cyclin D 1 suppression. Naunyn-Schmiedeberg’s Arch. Pharmacol..

[276] Chen Y., Yang S.-H., Hueng D.-Y., Syu J.-P., Liao C.-C., Wu Y.-C. (2014). Cordycepin induces apoptosis of C6 glioma cells through the adenosine 2A receptor-p53-caspase-7-PARP pathway. Chem. -Biol. Interact..

[277] Pan B.-S., Lin C.-Y., Huang B.-M. (2011). The effect of cordycepin on steroidogenesis and apoptosis in MA-10 mouse Leydig tumor cells. Evid. -Based Complementary Altern. Med..

[278] Zarkower D., Wickens M. (1987). Formation of mRNA 3′ termini: Stability and dissociation of a complex involving the AAUAAA sequence. Embo J..

[279] Kim S.O., Cha H.-J., Park C., Lee H., Hong S.H., Jeong S.-J., Park S.-H., Kim G.-Y., Leem S.-H., Jin C.-Y. (2019). Cordycepin induces apoptosis in human bladder cancer T24 cells through ROS-dependent inhibition of the PI3K/Akt signaling pathway. Biosci. Trends.

[280] Nasser M.I., Masood M., Wei W., Li X., Zhou Y., Liu B., Li J., Li X. (2017). Cordycepin induces apoptosis in SGC-7901 cells through mitochondrial extrinsic phosphorylation of PI3K/Akt by generating ROS. Int. J. Oncol..

[281] Wellham P.A., Kim D.-H., Brock M., de Moor C.H. (2019). Coupled biosynthesis of cordycepin and pentostatin in Cordyceps militaris: Implications for fungal biology and medicinal natural products. Ann. Transl. Med..

[282] Hiraoka W., Tanabe K., Kuwabara M., Sato F. (1988). Metabolic Effects of 3′-Deoxyadenosine (Cordycepin) and 2-Halo-3′-Deoxyadenosine on Repair of X-Ray-Induced Potentially Lethal Damage in Chinese Hamster V79 Cells. Radiat. Res..

[283] Kefford R.F., Taylor I.W., Fox R.M. (1983). Flow cytometric analysis of adenosine analogue lymphocytotoxicity. Cancer Res..

[284] Zieve G.W., Feeney R.J., Roemer E.J. (1987). Cordycepin disrupts the microtubule networks and arrests nil 8 hamster fibroblasts at the onset of mitosis. Cell Motil. Cytoskelet..

[285] Overgaard-Hansen K. (1964). The inhibition of 5-phosphoribosyl-1-pyrophosphate formation by cordycepin triphosphate in extracts of Ehrlich ascites tumor cells. Biochim. Et Biophys. Acta (Bba)-Spec. Sect. Nucleic Acids Relat. Subj..

[286] Lee J.B., Radhi M., Cipolla E., Gandhi R.D., Sarmad S., Zgair A., Kim T.H., Feng W., Qin C., Adrower C. (2019). A novel nucleoside rescue metabolic pathway may be responsible for therapeutic effect of orally administered cordycepin. Sci. Rep..

[287] Tsai Y.-J., Lin L.-C., Tsai T.-H. (2010). Pharmacokinetics of adenosine and cordycepin, a bioactive constituent of Cordyceps sinensis in rat. J. Agric. Food Chem..

[288] Li G., Nakagome I., Hirono S., Itoh T., Fujiwara R. (2015). Inhibition of adenosine deaminase (ADA)-mediated metabolism of cordycepin by natural substances. Pharmacol. Res. Perspect..

[289] Kodama E.N., McCaffrey R.P., Yusa K., Mitsuya H. (2000). Antileukemic activity and mechanism of action of cordycepin against terminal deoxynucleotidyl transferase-positive (TdT+) leukemic cells. Biochem. Pharmacol..

[290] Hirschhorn R. (1990). Adenosine deaminase deficiency. Immunodefic. Rev..

[291] Niu Y.-J., Tao R.-Y., Liu Q., Tian J.-Y., Ye F., Zhu P., Zhu H.-B. (2010). Improvement on lipid metabolic disorder by 3′-deoxyadenosine in high-fat-diet-induced fatty mice. Am. J. Chin. Med..

[292] Rodman L.E., Farnell D.R., Coyne J.M., Allan P.W., Hill D.L., Duncan K.L., Tomaszewski J.E., Smith A.C., Page J.G. (1997). Toxicity of cordycepin in combination with the adenosine deaminase inhibitor 2′-deoxycoformycin in beagle dogs. Toxicol. Appl. Pharmacol..

[293] Trigo P., Gutteridge W., Williamson J. (1971). The effects of cordycepin on malaria parasites. Trans. R. Soc. Trop. Med. Hyg..

[294] Inoue T., Murakami K., Fujii T. (1986). Mutagenic potential of cordycepin (3′-deoxyadenosine) in Salmonella and soybean tester strains. Mutat. Res. Lett..

[295] Ramesh T., Yoo S.-K., Kim S.-W., Hwang S.-Y., Sohn S.-H., Kim I.-W., Kim S.-K. (2012). Cordycepin (3′-deoxyadenosine) attenuates age-related oxidative stress and ameliorates antioxidant capacity in rats. Exp. Gerontol..

[296] Mannick J.B., Morris M., Hockey H.-U.P., Roma G., Beibel M., Kulmatycki K., Watkins M., Shavlakadze T., Zhou W., Quinn D. (2018). TORC1 inhibition enhances immune function and reduces infections in the elderly. Sci. Transl. Med..

[297] Sano N., Rajjou L., North H.M. (2020). Lost in translation: Physiological roles of stored mrnas in seed germination. Plants.

[298] Jung S.-M., Park S.-S., Kim W.-J., Moon S.-K. (2012). Ras/ERK1 pathway regulation of p27KIP1-mediated G1-phase cell-cycle arrest in cordycepin-induced inhibition of the proliferation of vascular smooth muscle cells. Eur. J. Pharmacol..

[299] Imesch P., Goerens A., Fink D., Fedier A. (2012). MLH1-deficient HCT116 colon tumor cells exhibit resistance to the cytostatic and cytotoxic effect of the poly (A) polymerase inhibitor cordycepin (3’-deoxyadenosine) in vitro. Oncol. Lett..

[300] Chen Y.-H., Wang J.-Y., Pan B.-S., Mu Y.-F., Lai M.-S., So E.C., Wong T.-S., Huang B.-M. (2013). Cordycepin enhances cisplatin apoptotic effect through caspase/MAPK pathways in human head and neck tumor cells. Oncotargets Ther..

[301] Wang F., Yin P., Lu Y., Zhou Z., Jiang C., Liu Y., Yu X. (2015). Cordycepin prevents oxidative stress-induced inhibition of osteogenesis. Oncotarget.

[302] Shao L.W., Huang L.H., Yan S., Jin J.D., Ren S.Y. (2016). Cordycepin induces apoptosis in human liver cancer HepG2 cells through extrinsic and intrinsic signaling pathways. Oncol. Lett..

[303] Hwang J.-H., Joo J.C., Kim D.J., Jo E., Yoo H.-S., Lee K.-B., Park S.J., Jang I.-S. (2016). Cordycepin promotes apoptosis by modulating the ERK-JNK signaling pathway via DUSP5 in renal cancer cells. Am. J. Cancer Res..

[304] Li S.Z., Ren J.W., Fei J., Zhang X.D., Du R.L. (2019). Cordycepin induces Bax-dependent apoptosis in colorectal cancer cells. Mol. Med. Rep..

[305] Wu W.-C., Hsiao J.-R., Lian Y.-Y., Lin C.-Y., Huang B.-M. (2007). The apoptotic effect of cordycepin on human OEC-M1 oral cancer cell line. Cancer Chemother. Pharmacol..

[306] Yamamoto K., Shichiri H., Uda A., Yamashita K., Nishioka T., Kume M., Makimoto H., Nakagawa T., Hirano T., Hirai M. (2015). Apoptotic effects of the extracts of cordyceps militaris via Erk phosphorylation in a renal cell carcinoma cell line. Phytother. Res..

[307] Tania M., Shawon J., Saif K., Kiefer R., Khorram M.S., Halim M.A., Khan M. (2019). Cordycepin downregulates Cdk-2 to interfere with cell cycle and increases apoptosis by generating ROS in cervical cancer cells: In vitro and in silico study. Curr. Cancer Drug Targets.

[308] Jiapeng T., Yiting L., Li Z. (2014). Optimization of fermentation conditions and purification of cordycepin from Cordyceps militaris. Prep. Biochem. Biotechnol..

[309] Lee S.Y., Debnath T., Kim S.-K., Lim B.O. (2013). Anti-cancer effect and apoptosis induction of cordycepin through DR3 pathway in the human colonic cancer cell HT-29. Food Chem. Toxicol..

[310] Baik J.-S., Kwon H.-Y., Kim K.-S. (2012). Cordycepin induces apoptosis in human neuroblastoma SK-N-BE (2)-C and melanoma SK-MEL-2 cells. Indian J. Biochem. Biophys..

[311] Shi P., Huang Z., Tan X., Chen G. (2008). Proteomic detection of changes in protein expression induced by cordycepin in human hepatocellular carcinoma BEL-7402 cells. Methods Find. Exp. Clin. Pharmacol..

[312] Müller W.E., Seibert G., Beyer R., Breter H.J., Maidhof A., Zahn R.K. (1977). Effect of cordycepin on nucleic acid metabolism in L5178Y cells and on nucleic acid-synthesizing enzyme systems. Cancer Res..

[313] Rosowsky A., Lazarus H., Yamashita A. (1976). Nucleosides. 1. 9-(3’-Alkyl-3’-deoxy-. beta.-D-ribofuranosyl) adenines as lipophilic analogs of cordycepin. Synthesis and preliminary biological studies. J. Med. Chem..

[314] Xu J.-C., Zhou X.-P., Wang X.-A., Xu M.-D., Chen T., Chen T.-Y., Zhou P.-H., Zhang Y.-Q. (2019). Cordycepin induces apoptosis and G2/M phase arrest through the ERK pathways in esophageal cancer cells. J. Cancer.

[315] Liu C., Qi M., Li L., Yuan Y., Wu X., Fu J. (2020). Natural cordycepin induces apoptosis and suppresses metastasis in breast cancer cells by inhibiting the Hedgehog pathway. Food Funct..

[316] Liao X., Tao L., Guo W., Wu Z.-X., Du H., Wang J., Zhang J., Chen H., Chen Z.-S., Lin L. (2020). Combination of Cordycepin and Apatinib synergistically inhibits NSCLC cells by down-regulating VEGF/PI3K/Akt signaling pathway. Front. Oncol..

[317] Wang C.-Y., Tsai S.-W., Chien H.-H., Chen T.-Y., Sheu S.-Y., So E.C., Huang B.-M. (2020). Cordycepin Inhibits Human Gestational Choriocarcinoma Cell Growth by Disrupting Centrosome Homeostasis. Drug Des. Dev. Ther..

[318] Kengkittipat W., Kaewmalun S., Khongkow M., Iempridee T., Jantimaporn A., Bunwatcharaphansakun P., Yostawonkul J., Yata T., Phoolcharoen W., Namdee K. (2021). Improvement of the multi-performance biocharacteristics of cordycepin using BiloNiosome-core/chitosan-shell hybrid nanocarriers. Colloids Surf. B: Biointerfaces.

[319] Gao Y., Chen D.-L., Zhou M., He M.-F., Huang S., Liao X.-Z., Zhang J.-X. (2020). Cordycepin enhances the chemosensitivity of esophageal cancer cells to cisplatin by inducing the activation of AMPK and suppressing the AKT signaling pathway. Cell Death Dis..

[320] Chang M.-M., Hong S.-Y., Yang S.-H., Wu C.-C., Wang C.-Y., Huang B.-M. (2020). Anti-Cancer Effect of Cordycepin on FGF9-Induced Testicular Tumorigenesis. Int. J. Mol. Sci..

[321] Chen Y., Chen Y.-C., Lin Y.-T., Huang S.-H., Wang S.-M. (2010). Cordycepin Induces Apoptosis of CGTH W-2 Thyroid Carcinoma Cells through the Calcium− Calpain− Caspase 7-PARP Pathway. J. Agric. Food Chem..

[322] Lu H., Li X., Zhang J., Shi H., Zhu X., He X. (2014). Effects of cordycepin on HepG2 and EA. hy926 cells: Potential antiproliferative, antimetastatic and anti-angiogenic effects on hepatocellular carcinoma. Oncol. Lett..

[323] Cho S.H., Kang I.-C. (2018). The inhibitory effect of Cordycepin on the proliferation of cisplatin-resistant A549 lung cancer cells. Biochem. Biophys. Res. Commun..

[324] Chen X., Wang Y., Liu J., Xu P., Zhang X.M., Tian Y.Y., Xue Y.M., Gao X.Y., Liu Y., Wang J.H. (2015). Synergistic effect of HMGB1 knockdown and cordycepin in the K562 human chronic myeloid leukemia cell line. Mol. Med. Rep..

[325] Chang M.M., Pan B.S., Wang C.Y., Huang B.M. (2019). Cordycepin-induced unfolded protein response-dependent cell death, and AKT/MAPK-mediated drug resistance in mouse testicular tumor cells. Cancer Med..

[326] Hu P., Chen W., Bao J., Jiang L., Wu L. (2014). Cordycepin modulates inflammatory and catabolic gene expression in interleukin-1beta-induced human chondrocytes from advanced-stage osteoarthritis: An in vitro study. Int. J. Clin. Exp. Pathol..

[327] Ryu E., Son M., Lee M., Lee K., Cho J.Y., Cho S., Lee S.K., Lee Y.M., Cho H., Sung G.-H. (2014). Cordycepin is a novel chemical suppressor of Epstein-Barr virus replication. Oncoscience.

[328] Dou C., Cao Z., Ding N., Hou T., Luo F., Kang F., Yang X., Jiang H., Xie Z., Hu M. (2016). Cordycepin prevents bone loss through inhibiting osteoclastogenesis by scavenging ROS generation. Nutrients.

[329] Olatunji O.J., Feng Y., Olatunji O.O., Tang J., Ouyang Z., Su Z. (2016). Cordycepin protects PC12 cells against 6-hydroxydopamine induced neurotoxicity via its antioxidant properties. Biomed. Pharmacother..

[330] Yu S.-B., Kim H.-J., Kang H.-M., Park B.-S., Lee J.-H., Kim I.-R. (2018). Cordycepin accelerates osteoblast mineralization and attenuates osteoclast differentiation in vitro. Evid. -Based Complementary Altern. Med..

[331] Jiang Y.-X., Chen Y., Yang Y., Chen X.-X., Zhang D.-D. (2019). Screening five Qi-Tonifying herbs on M2 phenotype macrophages. Evid. -Based Complementary Altern. Med..

[332] Zhou X., Meyer C.U., Schmidtke P., Zepp F. (2002). Effect of cordycepin on interleukin-10 production of human peripheral blood mononuclear cells. Eur. J. Pharmacol..

[333] Potten C., Darzynkiewicz Z., Sasaki K., Reshkin S., Tedone T., Correale M., Mangia A., Casavola V., Paradiso A. (1999). Association of pS2 (TFF1) release with breast tumour proliferative rate: In vitro and in vivo studies. Cell Prolif..

[334] Maytin E.V. (1992). Differential effects of heat shock and UVB light upon stress protein expression in epidermal keratinocytes. J. Biol. Chem..

[335] Jaiboonma A., Kaokaen P., Chaicharoenaudomrung N., Kunhorm P., Janebodin K., Noisa P., Jitprasertwong P. (2020). Cordycepin attenuates salivary hypofunction through the prevention of oxidative stress in human submandibular gland cells. Int. J. Med. Sci..

[336] Wang C.-H., Chang C.-H., Lin T.-L., Fu R.-H., Huang Y.-C., Chen S.-Y., Shyu W.-C., Liu S.-P. (2020). The novel application of cordycepin in maintaining stem cell pluripotency and increasing iPS cell generation efficiency. Sci. Rep..

[337] Seong D.B., Hong S., Muthusami S., Kim W.-D., Yu J.-R., Park W.-Y. (2016). Cordycepin increases radiosensitivity in cervical cancer cells by overriding or prolonging radiation-induced G2/M arrest. Eur. J. Pharmacol..

[338] Lee S.-J., Moon G.-S., Jung K.-H., Kim W.-J., Moon S.-K. (2010). c-Jun N-terminal kinase 1 is required for cordycepin-mediated induction of G2/M cell-cycle arrest via p21WAF1 expression in human colon cancer cells. Food Chem. Toxicol..

[339] Wang C.-W., Hsu W.-H., Tai C.-J. (2017). Antimetastatic effects of cordycepin mediated by the inhibition of mitochondrial activity and estrogen-related receptor α in human ovarian carcinoma cells. Oncotarget.

[340] Yoou M.-s., Yoon K.W., Choi Y., Kim H.-M., Jeong H.-J. (2017). Cordycepin diminishes thymic stromal lymphopoietin-induced interleukin-13 production. Eur. J. Pharmacol..

[341] Yoou M.-s., Jin M.H., Lee S.Y., Lee S.H., Kim B., Roh S.S., Choi I.H., Lee M.S., Kim H.-M., Jeong H.-J. (2016). Cordycepin suppresses thymic stromal lymphopoietin expression via blocking caspase-1 and receptor-interacting protein 2 signaling pathways in mast cells. Biol. Pharm. Bull..

[342] Shin S., Lee S., Kwon J., Moon S., Lee S., Lee C.-K., Cho K., Ha N.-J., Kim K. (2009). Cordycepin suppresses expression of diabetes regulating genes by inhibition of lipopolysaccharide-induced inflammation in macrophages. Immune Netw..

[343] Shin S., Moon S., Park Y., Kwon J., Lee S., Lee C.-K., Cho K., Ha N.-J., Kim K. (2009). Role of cordycepin and adenosine on the phenotypic switch of macrophages via induced anti-inflammatory cytokines. Immune Netw..

[344] Noh E.-M., Kim J.-S., Hur H., Park B.-H., Song E.-K., Han M.-K., Kwon K.-B., Yoo W.-H., Shim I.-K., Lee S. (2009). Cordycepin inhibits IL-1β-induced MMP-1 and MMP-3 expression in rheumatoid arthritis synovial fibroblasts. Rheumatology.

[345] Imamura K., Asai M., Sugamoto K., Matsumoto T., Yamasaki Y., Kamei I., Hattori T., Kishimoto M., Niisaka S., Kubo M. (2015). Suppressing effect of cordycepin on the lipopolysaccharide-induced nitric oxide production in RAW 264.7 cells. Biosci. Biotechnol. Biochem..

[346] Qing R., Huang Z., Tang Y., Xiang Q., Yang F. (2017). Cordycepin negatively modulates lipopolysaccharide-induced cytokine production by up-regulation of heme oxygenase-1. Int. Immunopharmacol..

[347] Yang J., Li Y.-z., Hylemon P.B., Zhang L.-y., Zhou H.-p. (2017). Cordycepin inhibits LPS-induced inflammatory responses by modulating NOD-Like Receptor Protein 3 inflammasome activation. Biomed. Pharmacother..

[348] Lu M.-Y., Chen C.-C., Lee L.-Y., Lin T.-W., Kuo C.-F. (2015). N 6-(2-Hydroxyethyl) adenosine in the medicinal mushroom Cordyceps cicadae attenuates lipopolysaccharide-stimulated pro-inflammatory responses by suppressing TLR4-mediated NF-κB signaling pathways. J. Nat. Prod..

[349] Seo M.J., Kim M.J., Lee H.H., Park J.U., Kang B.W., Kim G.-Y., Rhu E.J., Kim J.-I., Kim K.H., Jeong Y.K. (2013). Effect of cordycepin on the expression of the inflammatory cytokines TNF-alpha, IL-6, and IL-17A in C57BL/6 mice. J. Microbiol. Biotechnol..

[350] Ren Z., Cui J., Huo Z., Xue J., Cui H., Luo B., Jiang L., Yang R. (2012). Cordycepin suppresses TNF-α-induced NF-κB activation by reducing p65 transcriptional activity, inhibiting IκBα phosphorylation, and blocking IKKγ ubiquitination. Int. Immunopharmacol..

[351] Jeong M.-H., Seo M.J., Park J.U., Kang B.W., Kim K.-S., Lee J.Y., Kim G.-Y., Kim J.-I., Choi Y.H., Kim K.H. (2012). Effect of cordycepin purified from Cordyceps militaris on Th1 and Th2 cytokines in mouse splenocytes. J. Microbiol. Biotechnol..

[352] Aksamit R., Backlund P., Cantoni G. (1983). Chemotaxis and the synthesis of specific proteins are inhibited by 3-deazaadenosine and other adenosine analogs in a mouse macrophage cell line. J. Biol. Chem..

[353] Sugar A.M., McCaffrey R.P. (1998). Antifungal activity of 3′-deoxyadenosine (cordycepin). Antimicrob. Agents Chemother..

[354] Zhang D.-w., Deng H., Qi W., Zhao G.-y., Cao X.-r. (2015). Osteoprotective effect of cordycepin on estrogen deficiency-induced osteoporosis in vitro and in vivo. Biomed Res. Int..

[355] Chen Y.-X., Zhu D.-Y., Xu Z.-L., Yin J.-H., Yu X.-W., Mei J., Gao Y.-S., Zhang C.-Q. (2017). The protective effect of cordycepin on alcohol-induced osteonecrosis of the femoral head. Cell. Physiol. Biochem..

