# Supplementary material for: A Systematic Review of the Biological Effects of Cordycepin
Source: Molecules. 2021 Sep 28;26(19):5886. doi: 10.3390/molecules26195886 (PMC8510467; doi:10.3390/molecules26195886)
Supplement: Supplementary file 1 [file molecules-26-05886-s001.zip › molecules-1346401-supplementary.pdf]

database entry no	Title	PubMed entry (link) -compulsory	Paper ob-tained?	year	First author
2	Antifungal activity of 3'-deoxyadenosine (cordycepin).	https://www.ncbi.nlm.nih.gov/pubmed/9624488	y	1998	Sugal AM
3	Effect of cordycepin (3'-deoxyadenosine) on hematogenic lung metastatic model mice.	https://www.ncbi.nlm.nih.gov/pubmed/15796166	y	2005	Nakamura K
4	Antitumor effect of cordycepin (3'-deoxyadenosine) on mouse melanoma and lung carcinoma cells involves adenosine A3 receptor stimulation.	https://www.ncbi.nlm.nih.gov/pubmed/16475677	y	2006	Nakamura K
5	Cordycepin/Hydroxyurea synergy allows low dosage efficacy of cordycepin in MOLT-4 leukemia cells.	https://www.ncbi.nlm.nih.gov/pubmed/17970055	y	2007	Janek HW
6	Inhibitory effects of cordycepin (3'-deoxyadenosine), a component of Cordyceps militaris, on human platelet aggregation induced by thapsigargin.	https://www.ncbi.nlm.nih.gov/pubmed/18051324	y	2007	Jeong CH
7	Cordycepin Induced MA-10 Mouse Leydig Tumor Cell Apoptosis through Caspase-9 Pathway.	https://www.ncbi.nlm.nih.gov/pubmed/19131393	y	2011	Jen CY
8	Cordycepin attenuates neointimal formation by inhibiting reactive oxygen species-mediated responses in vascular smooth muscle cells in rats.	https://www.ncbi.nlm.nih.gov/pubmed/19305122	y	2009	Won KJ
9	Cordycepin inhibits UVB-induced matrix metalloproteinase expression by suppressing the NF-kappaB pathway in human dermal fibroblasts.	https://www.ncbi.nlm.nih.gov/pubmed/19381070	y	2009	Lee YR
10	Preclinical assessment of the treatment of second-stage African trypanosomiasis with cordycepin and deoxycoformycin.	https://www.ncbi.nlm.nih.gov/pubmed/19652702	y	2009	Vodnala SK
11	Cordycepin causes p21WAF1-mediated G2/M cell-cycle arrest by regulating c-Jun N-terminal kinase activation in human bladder cancer cells.	https://www.ncbi.nlm.nih.gov/pubmed/19733546	y	2009	Lee SJ
12	c-Jun N-terminal kinase 1 is required for cordycepin-mediated induction of G2/M cell-cycle arrest via p21WAF1 expression in human colon cancer cells.	https://www.ncbi.nlm.nih.gov/pubmed/19833164	y	2010	Lee SJ
13	Inhibitory effect of cordycepin on hematogenic metastasis of B16-F1 mouse melanoma cells accelerated by adenosine-5'-diphosphate.	https://www.ncbi.nlm.nih.gov/pubmed/19846919	y	2009	Yoshikawa N

14	Cordycepin inhibits protein synthesis and cell adhesion through effects on signal transduction.	https://www.ncbi.nlm.nih.gov/pubmed/19940154	y	2010	Wong YY
15	Cordycepin inhibits TPA-induced matrix metalloproteinase-9 expression by suppressing the MAPK/AP-1 pathway in MCF-7 human breast cancer cells.	https://www.ncbi.nlm.nih.gov/pubmed/20043135	y	2010	Noh EM
17	Role of Cordycepin and Adenosine on the Phenotypic Switch of Macrophages via Induced Anti-inflammatory Cytokines.	https://www.ncbi.nlm.nih.gov/pubmed/20157613	y	2009	Shin S
18	Pharmacokinetics of adenosine and cordycepin, a bioactive constituent of Cordyceps sinensis in rat.	https://www.ncbi.nlm.nih.gov/pubmed/20302371	y	2010	Tsai YJ
19	Cordycepin suppresses TNF-alpha-induced invasion, migration and matrix metalloproteinase-9 expression in human bladder cancer cells.	https://www.ncbi.nlm.nih.gov/pubmed/20564512	y	2010	Lee EJ
20	Cordycepin prevents hyperlipidemia in hamsters fed a high-fat diet via activation of AMP-activated protein kinase.	https://www.ncbi.nlm.nih.gov/pubmed/20724804	y	2010	Guo P
21	Binding of cordycepin monophosphate to AMP-activated protein kinase and its effect on AMP-activated protein kinase activation.	https://www.ncbi.nlm.nih.gov/pubmed/20738312	y	2010	Wang Z
22	Cordycepin induces apoptosis by enhancing JNK and p38 kinase activity and increasing the protein expression of Bcl-2 pro-apoptotic molecules.	https://www.ncbi.nlm.nih.gov/pubmed/20803769	y	2010	He W
23	Anti-inflammatory effects of cordycepin via suppression of inflammatory mediators in BV2 microglial cells.	https://www.ncbi.nlm.nih.gov/pubmed/20937401	y	2010	Jeong JW
24	Cordycepin induces apoptosis of CGTH W-2 thyroid carcinoma cells through the calcium-calpain-caspase 7-PARP pathway.	https://www.ncbi.nlm.nih.gov/pubmed/20961042	y	2010	Chen Y
25	Cordycepin (3'-deoxyadenosine), an inhibitor of mRNA polyadenylation, suppresses proliferation and activates apoptosis in human epithelial endometriotic cells in vitro.	https://www.ncbi.nlm.nih.gov/pubmed/21196698	y	2011	Imesch P
26	Induction of apoptosis by cordycepin via reactive oxygen species generation in human leukemia cells.	https://www.ncbi.nlm.nih.gov/pubmed/21310227	y	2011	Jeong JW
27	The in vivo and in vitro stimulatory effects of cordycepin on mouse leydig cell steroidogenesis.	https://www.ncbi.nlm.nih.gov/pubmed/21512251	y	2011	Leu SF
28	Cordycepin protects against cerebral ischemia/reperfusion injury in vivo and in vitro.	https://www.ncbi.nlm.nih.gov/pubmed/21554870	y	2011	Cheng Z

29	Aberrant, differential and bidirectional regulation of the unfolded protein response towards cell survival by 3'-deoxyadenosine.	https://www.ncbi.nlm.nih.gov/pubmed/21597460	y	2011	Kitamura M
30	Cordycepin blocks lung injury-associated inflammation and promotes BRCA1-deficient breast cancer cell killing by effectively inhibiting PARP.	https://www.ncbi.nlm.nih.gov/pubmed/21607289	y	2011	Kim H
31	Susceptibility of Trypanosoma evansi to cordycepin.	https://www.ncbi.nlm.nih.gov/pubmed/21620640	y	2011	Silva AS
32	The Effect of Cordycepin on Steroidogenesis and Apoptosis in MA-10 Mouse Leydig Tumor Cells.	https://www.ncbi.nlm.nih.gov/pubmed/21716681	y	2011	Pan BS
33	Cordycepin decreases activity of hippocampal CA1 pyramidal neuron through membrane hyperpolarization.	https://www.ncbi.nlm.nih.gov/pubmed/21896311	y	2011	Yao LH
34	Cordycepin-induced apoptosis and autophagy in breast cancer cells are independent of the estrogen receptor.	https://www.ncbi.nlm.nih.gov/pubmed/21933677	y	2011	Choi S
35	Suppression of Î±-MSH and IBMX-induced melanogenesis by cordycepin via inhibition of CREB and MITF, and activation of PI3K/Akt and ERK-dependent mechanisms.	https://www.ncbi.nlm.nih.gov/pubmed/21972008	y	2012	Jin M
36	Cordycepin inhibits renal interstitial myofibroblast activation probably by inducing hepatocyte growth factor expression.	https://www.ncbi.nlm.nih.gov/pubmed/22134049	y	2011	Li L
37	Cordycepin inhibits albumin-induced epithelial-mesenchymal transition of renal tubular epithelial cells by reducing reactive oxygen species production.	https://www.ncbi.nlm.nih.gov/pubmed/22149621	y	2012	Xiao L
38	Inhibition of migration and invasion of LNCaP human prostate carcinoma cells by cordycepin through inactivation of Akt.	https://www.ncbi.nlm.nih.gov/pubmed/22246470	y	2012	Jeong JW
39	Ras/ERK1 pathway regulation of p27KIP1-mediated G1-phase cell-cycle arrest in cordycepin-induced inhibition of the proliferation of vascular smooth muscle cells.	https://www.ncbi.nlm.nih.gov/pubmed/22366198	y	2012	Jung SM
40	Cordycepin stimulated steroidogenesis in MA-10 mouse Leydig tumor cells through the protein kinase C Pathway.	https://www.ncbi.nlm.nih.gov/pubmed/22512531	y	2012	Pao HY
41	Cordycepin as a sensitizer to tumour necrosis factor (TNF)-Î±-induced apoptosis through eukaryotic translation initiation factor 2Î± (eIF2Î±)- and mammalian target of rapamycin complex 1 (mTORC1)-mediated inhibition of nuclear factor (NF)-ÎºB.	https://www.ncbi.nlm.nih.gov/pubmed/22519596	y	2012	Kadomatsu M
42	Blockade of adipocyte differentiation by cordycepin.	https://www.ncbi.nlm.nih.gov/pubmed/22537056	y	2012	Takahashi S

43	Effect of cordycepin purified from Cordyceps militaris on Th1 and Th2 cytokines in mouse splenocytes.	https://www.ncbi.nlm.nih.gov/pubmed/22713995	y	2012	Ho JM
44	MLH1-deficient HCT116 colon tumor cells exhibit resistance to the cytostatic and cytotoxic effect of the poly(A) polymerase inhibitor cordycepin (3'-deoxyadenosine) in vitro.	https://www.ncbi.nlm.nih.gov/pubmed/22740928	y	2012	Imesch P
45	Effect of long-term administration of cordycepin from Cordyceps militaris on testicular function in middle-aged rats.	https://www.ncbi.nlm.nih.gov/pubmed/22872590	y	2012	Sohn SH
46	Cordycepin (3'-deoxyadenosine) attenuates age-related oxidative stress and ameliorates antioxidant capacity in rats.	https://www.ncbi.nlm.nih.gov/pubmed/23000874	y	2012	Ramesh T
47	Cordycepin suppresses TNF-Î±-induced NF-ÎºB activation by reducing p65 transcriptional activity, inhibiting IÎºBÎ± phosphorylation, and blocking IKKÎ³ ubiquitination.	https://www.ncbi.nlm.nih.gov/pubmed/23102662	y	2012	Ren Z
48	Inhibition of polyadenylation reduces inflammatory gene induction.	https://www.ncbi.nlm.nih.gov/pubmed/23118416	y	2012	Kondrashov A
49	Effect of cordycepin on the expression of the inflammatory cytokines TNF-alpha, IL-6, and IL-17A in C57BL/6 mice.	https://www.ncbi.nlm.nih.gov/pubmed/23412056	y	2013	Jeong SM
50	Blockade of Smad signaling by 3'-deoxyadenosine: a mechanism for its anti-fibrotic potential.	https://www.ncbi.nlm.nih.gov/pubmed/23439432	y	2013	Gu L
51	Cordycepin Increases Nonrapid Eye Movement Sleep via Adenosine Receptors in Rats.	https://www.ncbi.nlm.nih.gov/pubmed/23710239	y	2013	Hu Z
52	Cordycepin enhances cisplatin apoptotic effect through caspase/MAPK pathways in human head and neck tumor cells.	https://www.ncbi.nlm.nih.gov/pubmed/23926438	y	2013	Chen YH
53	Cordycepin regulates GSK-3Î²/Î²-catenin signaling in human leukemia cells.	https://www.ncbi.nlm.nih.gov/pubmed/24086728	y	2013	Ko BS
54	Cordycepin, 3'-deoxyadenosine, prevents rat hearts from ischemia/reperfusion injury via activation of Akt/GSK-3Î²/p70S6K signaling pathway and HO-1 expression.	https://www.ncbi.nlm.nih.gov/pubmed/24178833	y	2015	Park ES
55	Cordycepin-mediated transcriptional regulation of human GD3 synthase (hST8Sia I) in human neuroblastoma SK-N-BE(2)-C cells.	https://www.ncbi.nlm.nih.gov/pubmed/24225218	y	2014	Baik JS
56	Structure-activity relationships of synthetic cordycepin analogues as experimental therapeutics for African trypanosomiasis.	https://www.ncbi.nlm.nih.gov/pubmed/24283924	y	2013	Vodnala SK

57	Cordycepin activates AMP-activated protein kinase (AMPK) via interaction with the Î³1 subunit.	https://www.ncbi.nlm.nih.gov/pubmed/24286368	y	2014	Wu C
58	Inhibitory effect of cordycepin on experimental hepatic metastasis of B16-F0 mouse melanoma cells.	https://www.ncbi.nlm.nih.gov/pubmed/24292575	y	2013	Sato A
59	Cordycepin prevented IL-Î²-induced expression of inflammatory mediators in human osteoarthritis chondrocytes.	https://www.ncbi.nlm.nih.gov/pubmed/24346509	y	2014	Ying X
60	Modulation effects of cordycepin on the skeletal muscle contraction of toad gastrocnemius muscle.	https://www.ncbi.nlm.nih.gov/pubmed/24447979	y	2014	Yao LH
61	The neuroprotective effects of cordycepin inhibit glutamate-induced oxidative and ER stress-associated apoptosis in hippocampal HT22 cells.	https://www.ncbi.nlm.nih.gov/pubmed/24486958	y	2014	Jin ML
62	Cordycepin (3'-deoxyadenosine) down-regulates the proinflammatory cytokines in inflammation-induced osteoporosis model.	https://www.ncbi.nlm.nih.gov/pubmed/24493324	y	2014	Zhang DW
63	Cordycepin induces apoptosis of C6 glioma cells through the adenosine 2A receptor-p53-caspase-7-PARP pathway.	https://www.ncbi.nlm.nih.gov/pubmed/24704558	y	2014	Chen Y
64	Effects of cordycepin on HepG2 and EA.hy926 cells: Potential antiproliferative, antimetastatic and anti-angiogenic effects on hepatocellular carcinoma.	https://www.ncbi.nlm.nih.gov/pubmed/24765175	y	2014	Lu H
65	Involvement of autophagy in cordycepin-induced apoptosis in human prostate carcinoma LNCaP cells.	https://www.ncbi.nlm.nih.gov/pubmed/24973666	y	2014	Lee HH
66	Cordycepin inhibits lipopolysaccharide (LPS)-induced tumor necrosis factor (TNF)-Î± production via activating amp-activated protein kinase (AMPK) signaling.	https://www.ncbi.nlm.nih.gov/pubmed/25007068	y	2014	Zhang JL
68	Cordycepin down-regulates multiple drug resistant (MDR)/HIF-1Î± through regulating AMPK/mTORC1 signaling in GBC-SD gallbladder cancer cells.	https://www.ncbi.nlm.nih.gov/pubmed/25046749	y	2014	Wu WD
69	Cordycepin induces S phase arrest and apoptosis in human gallbladder cancer cells.	https://www.ncbi.nlm.nih.gov/pubmed/25090123	y	2014	Wang XA
70	Cordyceptin induces apoptosis through repressing hTERT expression and inducing extranuclear export of hTERT.	https://www.ncbi.nlm.nih.gov/pubmed/25282637	y	2014	Jang KJ
71	Anti-inflammatory effects of cordycepin in lipopolysaccharide-stimulated RAW 264.7 macrophages through Toll-like receptor 4-mediated suppression of mitogen-activated protein kinases and NF-ÎºB signaling pathways.	https://www.ncbi.nlm.nih.gov/pubmed/25342887	y	2014	Choi YH

72	Cordycepin modulates inflammatory and catabolic gene expression in interleukin-1beta-induced human chondrocytes from advanced-stage osteoarthritis: an in vitro study.	https://www.ncbi.nlm.nih.gov/pubmed/25400736	y	2014	Hu P
73	The effects of cordycepin on ovalbumin-induced allergic inflammation by strengthening Treg response and suppressing Th17 responses in ovalbumin-sensitized mice.	https://www.ncbi.nlm.nih.gov/pubmed/25417131	y	2015	Tianzhu Z
74	Synergistic property of cordycepin in cultivated Cordyceps militaris-mediated apoptosis in human leukemia cells.	https://www.ncbi.nlm.nih.gov/pubmed/25442260	y	2014	Chou SM
75	Antidepressant-like effects of cordycepin in a mice model of chronic unpredictable mild stress.	https://www.ncbi.nlm.nih.gov/pubmed/25587342	y	2014	Tianzhu Z
76	Cordycepin induces cell cycle arrest and apoptosis by inducing DNA damage and up-regulation of p53 in Leukemia cells.	https://www.ncbi.nlm.nih.gov/pubmed/25590866	y	2015	Liao Y
77	Cordycepin is a novel chemical suppressor of Epstein-Barr virus replication.	https://www.ncbi.nlm.nih.gov/pubmed/25621301	y	2014	Ryu E
78	Apoptosis of Bel-7402 human hepatoma cells induced by a ruthenium(II) complex coordinated by cordycepin through the p53 pathway.	https://www.ncbi.nlm.nih.gov/pubmed/25651236	y	2015	Lu Q
79	Suppressing effect of cordycepin on the lipopolysaccharide-induced nitric oxide production in RAW 264.7 cells.	https://www.ncbi.nlm.nih.gov/pubmed/25652735	y	2015	Imamura K
80	Cordyceps militaris mushroom and cordycepin inhibit RANKL-induced osteoclast differentiation.	https://www.ncbi.nlm.nih.gov/pubmed/25789604	y	2015	Kim J
81	Cordycepin (3'-deoxyadenosine) suppressed HMGA2, Twist1 and ZEB1-dependent melanoma invasion and metastasis by targeting miR-33b.	https://www.ncbi.nlm.nih.gov/pubmed/25868853	y	2015	Zhang P
82	Osteoprotective effect of cordycepin on estrogen deficiency-induced osteoporosis in vitro and in vivo.	https://www.ncbi.nlm.nih.gov/pubmed/25874211	y	2015	Zhang DW
83	Cordycepin alleviates airway hyperreactivity in a murine model of asthma by attenuating the inflammatory process.	https://www.ncbi.nlm.nih.gov/pubmed/25912153	y	2015	Yang X
84	Effects of cordycepin on the microglia-overactivation-induced impairments of growth and development of hippocampal cultured neurons.	https://www.ncbi.nlm.nih.gov/pubmed/25932642	y	2015	Peng J
85	Cordycepin from Cordyceps militaris prevents hyperglycemia in alloxan-induced diabetic mice.	https://www.ncbi.nlm.nih.gov/pubmed/25940982	y	2015	Ma L

86	Cordycepin Decreases Compound Action Potential Conduction of Frog Sciatic Nerve In Vitro Involving Ca (2+) -Dependent Mechanisms.	https://www.ncbi.nlm.nih.gov/pubmed/26078886	y	2015	Yao LH
87	Synergistic effect of HMGB1 knockdown and cordycepin in the K562 human chronic myeloid leukemia cell line.	https://www.ncbi.nlm.nih.gov/pubmed/26081986	y	2015	Chen X
88	Cordycepin protected against the TNF-Î±-induced inhibition of osteogenic differentiation of human adipose-derived mesenchymal stem cells.	https://www.ncbi.nlm.nih.gov/pubmed/26130747	y	2015	Yang J
89	Cordycepin induces apoptosis and autophagy in human neuroblastoma SK-N-SH and BE(2)-M17 cells.	https://www.ncbi.nlm.nih.gov/pubmed/26137103	y	2015	Li Y
90	Cordycepin induced MA-10 mouse Leydig tumor cell apoptosis by regulating p38 MAPKs and PI3K/AKT signaling pathways.	https://www.ncbi.nlm.nih.gov/pubmed/26303320	y	2015	Pan B
91	3'-Deoxyadenosine (Cordycepin) Produces a Rapid and Robust Antidepressant Effect via Enhancing Prefrontal AMPA Receptor Signaling Pathway.	https://www.ncbi.nlm.nih.gov/pubmed/26443809	y	2016	Li B
92	Cordycepin prevents oxidative stress-induced inhibition of osteogenesis.	https://www.ncbi.nlm.nih.gov/pubmed/26462178	y	2015	Wang F
93	Cordycepin increases radiosensitivity in cervical cancer cells by overriding or prolonging radiation-induced G2/M arrest.	https://www.ncbi.nlm.nih.gov/pubmed/26688569	y	2016	Seong DB
94	Cordycepin Suppresses Thymic Stromal Lymphopoietin Expression via Blocking Caspase-1 and Receptor-Interacting Protein 2 Signaling Pathways in Mast Cells.	https://www.ncbi.nlm.nih.gov/pubmed/26725432	y	2016	Yoou MS
95	The anti-tumor effects of cordycepin-loaded liposomes on the growth of hepatoma 22 tumors in mice and human hepatoma BEL-7402 cells in culture.	https://www.ncbi.nlm.nih.gov/pubmed/26984179	y	2016	Wu PK
97	Cordycepin enhances Epstein-Barr virus lytic infection and Epstein-Barr virus-positive tumor treatment efficacy by doxorubicin.	https://www.ncbi.nlm.nih.gov/pubmed/27063964	y	2016	Du Y
98	Cordycepin Prevents Bone Loss through Inhibiting Osteoclastogenesis by Scavenging ROS Generation.	https://www.ncbi.nlm.nih.gov/pubmed/27104563	y	2016	Dou C
99	The effects of cordycepin on the cell proliferation, migration and apoptosis in human lung cancer cell lines A549 and NCI-H460.	https://www.ncbi.nlm.nih.gov/pubmed/27138740	y	2016	Tao X
100	Cordycepin inhibits LPS-induced inflammatory and matrix degradation in the intervertebral disc.	https://www.ncbi.nlm.nih.gov/pubmed/27190710	y	2016	Li Y
101	Cordycepin protects PC12 cells against 6-hydroxydopamine induced neurotoxicity via its antioxidant properties.	https://www.ncbi.nlm.nih.gov/pubmed/27261571	y	2016	Olatunji OJ

102	Cordycepin inhibits chondrocyte hypertrophy of mesenchymal stem cells through PI3K/Bapx1 and Notch signaling pathway.	https://www.ncbi.nlm.nih.gov/pubmed/27439604	y	2016	Cao Z
103	Cordycepin induces apoptosis in human liver cancer HepG2 cells through extrinsic and intrinsic signaling pathways.	https://www.ncbi.nlm.nih.gov/pubmed/27446383	y	2016	Shao LW
104	Cordycepin attenuates traumatic brain injury-induced impairments of blood-brain barrier integrity in rats.	https://www.ncbi.nlm.nih.gov/pubmed/27646481	y	2016	Yuan J
105	Cordycepin promotes apoptosis by modulating the ERK-JNK signaling pathway via DUSP5 in renal cancer cells.	https://www.ncbi.nlm.nih.gov/pubmed/27648363	y	2016	Hwang JH
106	Cordycepin Induces Apoptosis and Inhibits Proliferation of Human Lung Cancer Cell Line H1975 via Inhibiting the Phosphorylation of EGFR.	https://www.ncbi.nlm.nih.gov/pubmed/27689974	y	2016	Wang Z
107	Antimetastatic effects of cordycepin mediated by the inhibition of mitochondrial activity and estrogen-related receptor Î± in human ovarian carcinoma cells.	https://www.ncbi.nlm.nih.gov/pubmed/27966445	y	2017	Wang CW
108	Anti-effects of cordycepin to hypoxia-induced membrane depolarization on hippocampal CA1 pyramidal neuron.	https://www.ncbi.nlm.nih.gov/pubmed/27988284	y	2017	Chen C
109	Cordycepin induces autophagy-mediated c-FLIPL degradation and leads to apoptosis in human non-small cell lung cancer cells.	https://www.ncbi.nlm.nih.gov/pubmed/28035061	y	2017	Yu X
110	Cordycepin inhibits migration of human glioblastoma cells by affecting lysosomal degradation and protein phosphatase activation.	https://www.ncbi.nlm.nih.gov/pubmed/28068557	y	2017	Hueng DY
111	Cordycepin induces apoptosis by caveolin-1-mediated JNK regulation of Foxo3a in human lung adenocarcinoma.	https://www.ncbi.nlm.nih.gov/pubmed/28099944	y	2017	Joo JC
112	Cordycepin inhibits airway remodeling in a rat model of chronic asthma.	https://www.ncbi.nlm.nih.gov/pubmed/28119235	y	2017	Fei X
113	Cordycepin induces apoptosis in SGCâ€'7901 cells through mitochondrial extrinsic phosphorylation of PI3K/Akt by generating ROS.	https://www.ncbi.nlm.nih.gov/pubmed/28197639	y	2017	Nasser MI
114	Cordycepin diminishes thymic stromal lymphopoietin-induced interleukin-13 production.	https://www.ncbi.nlm.nih.gov/pubmed/28219709	y	2017	Yoou MS
115	Cordycepin disrupts leukemia association with mesenchymal stromal cells and eliminates leukemia stem cell activity.	https://www.ncbi.nlm.nih.gov/pubmed/28266575	y	2017	Liang SM
116	Cordycepin negatively modulates lipopolysaccharide-induced cytokine production by up-regulation of heme oxygenase-1.	https://www.ncbi.nlm.nih.gov/pubmed/28351780	y	2017	Qing R

118	Cordycepin induces human lung cancer cell apoptosis by inhibiting nitric oxide mediated ERK/Slug signaling pathway.	https://www.ncbi.nlm.nih.gov/pubmed/28401001	y	2017	Hwang JH
119	Cordycepin confers neuroprotection in mice models of intracerebral hemorrhage via suppressing NLRP3 inflammasome activation.	https://www.ncbi.nlm.nih.gov/pubmed/28401330	y	2017	Cheng Y
120	Protective Effect of Cordycepin on Experimental Testicular Ischemia/Reperfusion Injury in Rats.	https://www.ncbi.nlm.nih.gov/pubmed/28402715	y	2017	Okur MH
121	Metronomic Cordycepin Therapy Prolongs Survival of Oral Cancer-Bearing Mice and Inhibits Epithelial-Mesenchymal Transition.	https://www.ncbi.nlm.nih.gov/pubmed/28406456	y	2017	Su NW
122	The Protective Effect of Cordycepin on D-Galactosamine/Lipopolysaccharide-Induced Acute Liver Injury.	https://www.ncbi.nlm.nih.gov/pubmed/28522898	y	2017	Li J
123	Development of Cordycepin Formulations for Preclinical and Clinical Studies.	https://www.ncbi.nlm.nih.gov/pubmed/28560504	y	2017	Lee JB
124	Cordycepin induces apoptosis in human bladder cancer cells via activation of A3 adenosine receptors.	https://www.ncbi.nlm.nih.gov/pubmed/28714368	y	2017	Cao HL
125	Cordycepin prevents postoperative formation of intra-abdominal adhesion in a rat model: An experimental study.	https://www.ncbi.nlm.nih.gov/pubmed/28762461	y	2017	Arslan S
126	The Protective Effect of Cordycepin On Alcohol-Induced Osteonecrosis of the Femoral Head.	https://www.ncbi.nlm.nih.gov/pubmed/28848161	y	2017	Chen YX
127	Targeted Delivery of Cordycepin to Liver Cancer Cells Using Transferrin-conjugated Liposomes.	https://www.ncbi.nlm.nih.gov/pubmed/28870956	y	2017	Bi Y
128	Cordycepin inhibits cell growth and induces apoptosis in human cholangiocarcinoma.	https://www.ncbi.nlm.nih.gov/pubmed/28895407	y	2017	Wang C
129	Cordycepin induces apoptosis of human acute monocytic leukemia cells via downregulation of the ERK/Akt signaling pathway.	https://www.ncbi.nlm.nih.gov/pubmed/28912858	y	2017	Wang Y 2017
130	Cordycepin inhibits LPS-induced inflammatory responses by modulating NOD-Like Receptor Protein 3 inflammasome activation.	https://www.ncbi.nlm.nih.gov/pubmed/28962083	y	2017	Yang J
131	Cordycepin inhibits vascular adhesion molecule expression in TNF-Î±-stimulated vascular muscle cells.	https://www.ncbi.nlm.nih.gov/pubmed/28962164	y	2017	Yan LJ
132	Anti-hepatocarcinoma effect of cordycepin against NDEA-induced hepatocellular carcinomas via the PI3K/Akt/mTOR and Nrf2/HO-1/NF-ÎºB pathway in mice.	https://www.ncbi.nlm.nih.gov/pubmed/28968944	y	2017	Zheng Y
133	Cordycepin induces apoptotic cell death of human brain cancer through the modulation of autophagy.	https://www.ncbi.nlm.nih.gov/pubmed/28987792	y	2018	Chaicharoenaudomrung N

135	Treatment with 3'-deoxyadenosine and deoxycoformycin in mice infected by Trypanosoma cruzi and its side effect on purinergic enzymes.	https://www.ncbi.nlm.nih.gov/pubmed/29051060	y	2017	Carmo GM
136	Cordycepin inhibits LPS-induced acute lung injury by inhibiting inflammation and oxidative stress.	https://www.ncbi.nlm.nih.gov/pubmed/29054740	y	2018	Lei J
139	Effects of cordycepin on spontaneous alternation behavior and adenosine receptors expression in hippocampus.	https://www.ncbi.nlm.nih.gov/pubmed/29174913	y	2018	Gao ZP
140	Cordycepin and a preparation from Cordyceps militaris inhibit malignant transformation and proliferation by decreasing EGFR and IL-17RA signaling in a murine oral cancer model.	https://www.ncbi.nlm.nih.gov/pubmed/29212184	y	2017	Hsu PY
141	Cordycepin stimulates autophagy in macrophages and prevents atherosclerotic plaque formation in ApoE(-/-) mice.	https://www.ncbi.nlm.nih.gov/pubmed/29212261	y	2017	Li X
142	Modulation Effects of Cordycepin on Voltage-Gated Sodium Channels in Rat Hippocampal CA1 Pyramidal Neurons in the Presence/Absence of Oxygen.	https://www.ncbi.nlm.nih.gov/pubmed/29225974	y	2017	Liu ZB
143	Cordycepin rescues lidocaine-induced neurotoxicity in dorsal root ganglion by interacting with inflammatory signaling pathway MMP3.	https://www.ncbi.nlm.nih.gov/pubmed/29382532	y	2018	Kan H
144	The inhibitory effect of Cordycepin on the proliferation of cisplatin-resistant A549 lung cancer cells.	https://www.ncbi.nlm.nih.gov/pubmed/29496448	y	2018	Cho SH
145	Experimental and In Silico Analysis of Cordycepin and its Derivatives as Endometrial Cancer Treatment.	https://www.ncbi.nlm.nih.gov/pubmed/29673423	y	2019	Fong P
146	Cordycepin alleviates lipopolysaccharide-induced acute lung injury via Nrf2/HO-1 pathway.	https://www.ncbi.nlm.nih.gov/pubmed/29702279	y	2018	Qing R
147	Cordycepin induces apoptosis of human ovarian cancer cells by inhibiting CCL5-mediated Akt/NF-Î°B signaling pathway.	https://www.ncbi.nlm.nih.gov/pubmed/29844932	y	2018	Cui ZY
148	Neuroprotective effects of cordycepin inhibit AÎ²-induced apoptosis in hippocampal neurons.	https://www.ncbi.nlm.nih.gov/pubmed/30031108	y	2018	Song H
149	Î²-catenin contributes to cordycepin-induced MGMT inhibition and reduction of temozolomide resistance in glioma cells by increasing intracellular reactive oxygen species.	https://www.ncbi.nlm.nih.gov/pubmed/30081068	y	2018	Bi Y
150	Cordycepin induces apoptosis in human pancreatic cancer cells via the mitochondrial-mediated intrinsic pathway and suppresses tumor growth in vivo.	https://www.ncbi.nlm.nih.gov/pubmed/30122940	y	2018	Zhang y
151	Cordycepin ameliorates skin inflammation in a DNFB-challenged murine model of atopic dermatitis.	https://www.ncbi.nlm.nih.gov/pubmed/30183461	y	2018	Han NR

152	Cordycepin activates autophagy through AMPK phosphorylation to reduce abnormalities in Machado-Joseph disease models.	https://www.ncbi.nlm.nih.gov/pubmed/30219871	y	2019	Marcelo A
153	Cordycepin inhibits lipopolysaccharide-induced cell migration and invasion in human colorectal carcinoma HCT-116 cells through down-regulation of prostaglandin E2 receptor EP4.	https://www.ncbi.nlm.nih.gov/pubmed/30269738	y	2018	Jeong JW
154	Cordycepin Augments the Chemosensitivity of Human Glioma Cells to Temozolomide by Activating AMPK and Inhibiting the AKT Signaling Pathway.	https://www.ncbi.nlm.nih.gov/pubmed/30336060	y	2018	Bi Y
155	Cordycepin Accelerates Osteoblast Mineralization and Attenuates Osteoclast Differentiation In Vitro.	https://www.ncbi.nlm.nih.gov/pubmed/30410556	y	2018	Yu SB
156	Cordycepin reduces weight through regulating gut microbiota in high-fat diet-induced obese rats.	https://www.ncbi.nlm.nih.gov/pubmed/30522511	y	2018	An Y
157	Cordycepin sensitizes breast cancer cells toward irradiation through elevating ROS production involving Nrf2.	https://www.ncbi.nlm.nih.gov/pubmed/30529626	y	2018	Dong J
158	Cordycepin induces Baxâ€‘dependent apoptosis in colorectal cancer cells.	https://www.ncbi.nlm.nih.gov/pubmed/30535479	y	2019	Li SZ
159	Cordycepin, isolated from medicinal fungus Cordyceps sinensis, enhances radiosensitivity of oral cancer associated with modulation of DNA damage repair.	https://www.ncbi.nlm.nih.gov/pubmed/30576710	y	2018	Su NW
160	Cordycepin Suppresses Endothelial Cell Proliferation, Migration, Angiogenesis, and Tumor Growth by Regulating Focal Adhesion Kinase and p53.	https://www.ncbi.nlm.nih.gov/pubmed/30717276	y	2019	Lin YT
162	Cordycepin promotes browning of white adipose tissue through an AMP-activated protein kinase (AMPK)-dependent pathway.	https://www.ncbi.nlm.nih.gov/pubmed/30766785	y	2019	Qi J
163	Cordycepin mitigates MPTP-induced Parkinson's disease through inhibiting TLR/NF-ÎºB signaling pathway.	https://www.ncbi.nlm.nih.gov/pubmed/30790609	y	2019	Cheng C
164	Cordycepin (3'-deoxyadenosine) and pentostatin (deoxycoformycin) against Trypanosoma cruzi.	https://www.ncbi.nlm.nih.gov/pubmed/30825499	y	2019	Carmo GM
165	The cordycepin derivative IMM-H007 improves endothelial dysfunction by suppressing vascular inflammation and promoting AMPK-dependent eNOS activation in high-fat diet-fed ApoE knockout mice.	https://www.ncbi.nlm.nih.gov/pubmed/30826323	y	2019	Wang MJ
166	Cordycepin suppresses cell proliferation and migration by targeting CLEC2 in human gastric cancer cells via Akt signaling pathway.	https://www.ncbi.nlm.nih.gov/pubmed/30878262	y	2019	Wang Y

167	The polyadenylation inhibitor cordycepin reduces pain, inflammation and joint pathology in rodent models of osteoarthritis.	https://www.ncbi.nlm.nih.gov/pubmed/30886197	y	2019	Ashraf S
168	Cordycepin, an Active Constituent of Nutrient Powerhouse and Potential Medicinal Mushroom Cordyceps militaris Linn., Ameliorates Age-Related Testicular Dysfunction in Rats.	https://www.ncbi.nlm.nih.gov/pubmed/31018574	y	2019	Kopalli SR
169	Cordycepin blocks recovery of non-heat-shock mRNA translation following heat shock in Drosophila.	https://www.ncbi.nlm.nih.gov/pubmed/8521843	y	1995	Duncan RF
170	The polyadenylation inhibitor cordycepin (3'dA) causes a decline in c-MYC mRNA levels without affecting c-MYC protein levels.	https://www.ncbi.nlm.nih.gov/pubmed/9926926	y	1999	Ioannidis P
171	Cordyceps militaris Improves Chronic Kidney Disease by Affecting TLR4/NF-ÎºB Redox Signaling Pathway.	https://www.ncbi.nlm.nih.gov/pubmed/31049139	y	2019	Sun T
172	Neuroprotection of cordycepin in NMDA-induced excitotoxicity by modulating adenosine A(1) receptors.	https://www.ncbi.nlm.nih.gov/pubmed/30978320	y	2019	Dong ZW
173	Cordycepin Inhibits Drug-resistance Non-small Cell Lung Cancer Progression by Activating AMPK Signaling Pathway.	https://www.ncbi.nlm.nih.gov/pubmed/30974169	y	2019	Wei C
174	Antimicrobial effect and proposed action mechanism of cordycepin against Escherichia coli and Bacillus subtilis.	https://www.ncbi.nlm.nih.gov/pubmed/30929229	y	2019	Jiang Q
181	Cordycepin inhibits lipopolysaccharide-induced inflammation by the suppression of NF-kappaB through Akt and p38 inhibition in RAW 264.7 macrophage cells.	https://www.ncbi.nlm.nih.gov/pubmed/16899239	y	2006	Kim HG
183	Cordycepin (3'-deoxyadenosine) inhibits human platelet aggregation in a cyclic AMP- and cyclic GMP-dependent manner.	https://www.ncbi.nlm.nih.gov/pubmed/17229422	y	2007	Cho HJ
184	Cordycepin inhibits vascular smooth muscle cell proliferation.	https://www.ncbi.nlm.nih.gov/pubmed/18782572	y	2008	Chang W
185	Effect of Cordyceps militaris supplementation on sperm production, sperm motility and hormones in Sprague-Dawley rats.	https://www.ncbi.nlm.nih.gov/pubmed/19051352	y	2008	Chang Y
186	Synthesis and properties of cordycepin intercalates of Mg-Al-nitrate layered double hydroxides.	https://www.ncbi.nlm.nih.gov/pubmed/16889916	y	2006	Yang QZ
187	Synthesis and pharmacokinetic evaluation of novel N-acyl-cordycepin derivatives with a normal alkyl chain.	https://www.ncbi.nlm.nih.gov/pubmed/18599159	y	2009	Wei HP
188	Effects of Cordyceps sinensis, Cordyceps militaris and their isolated compounds on ion transport in Calu-3 human airway epithelial cells.	https://www.ncbi.nlm.nih.gov/pubmed/18358654	y	2008	Yue GG

189	Improvement of sperm production in subfertile boars by Cordyceps militaris supplement.	https://www.ncbi.nlm.nih.gov/pubmed/17708629	y	2007	Lin WH
190	Cordycepin inhibits IL-1beta-induced MMP-1 and MMP-3 expression in rheumatoid arthritis synovial fibroblasts.	https://www.ncbi.nlm.nih.gov/pubmed/19056796	y	2009	Noh EM
191	Cordycepin (3'-deoxyadenosine) inhibits human platelet aggregation induced by U46619, a TXA2 analogue.	https://www.ncbi.nlm.nih.gov/pubmed/17331333	y	2006	Cho HJ
192	Cordycepin induced eryptosis in mouse erythrocytes through a Ca2+-dependent pathway without caspase-3 activation.	https://www.ncbi.nlm.nih.gov/pubmed/17541556	y	2007	Lui JC
193	A Phytochemically characterized extract of Cordyceps militaris and cordycepin protect hippocampal neurons from ischemic injury in gerbils.	https://www.ncbi.nlm.nih.gov/pubmed/18214814	y	2007	Hwang IK
194	The effect of the polyadenylation inhibitor cordycepin on human Molt-4 and Daudi leukaemia and lymphoma cell lines.	https://www.ncbi.nlm.nih.gov/pubmed/17564706	y	2008	Thomadaki H
195	Cordycepin is an immunoregulatory active ingredient of Cordyceps sinensis.	https://www.ncbi.nlm.nih.gov/pubmed/19051361	y	2008	Zhou X
196	Cordycepin (3'-deoxyadenosine) inhibits the growth of B16-BL6 mouse melanoma cells through the stimulation of adenosine A3 receptor followed by glycogen synthase kinase-3beta activation and cyclin D1 suppression.	https://www.ncbi.nlm.nih.gov/pubmed/18084742	y	2008	Yoshikawa N
197	The apoptotic effect of cordycepin on human OEC-M1 oral cancer cell line.	https://www.ncbi.nlm.nih.gov/pubmed/17031645	y	2007	Wu WC
198	Treatment of African trypanosomiasis with cordycepin and adenosine deaminase inhibitors in a mouse model.	https://www.ncbi.nlm.nih.gov/pubmed/16206083	y	2005	Rottenberg ME
199	Toxicity of cordycepin in combination with the adenosine deaminase inhibitor 2'-deoxycoformycin in beagle dogs.	https://www.ncbi.nlm.nih.gov/pubmed/9356305	y	1997	Rodman LE
200	Antileukemic activity and mechanism of action of cordycepin against terminal deoxynucleotidyl transferase-positive (TdT+) leukemic cells.	https://www.ncbi.nlm.nih.gov/pubmed/10609556	y	2000	Kodama EN
201	Effect of cordycepin on interleukin-10 production of human peripheral blood mononuclear cells.	https://www.ncbi.nlm.nih.gov/pubmed/12398919	y	2002	Zhou X
202	Effect of cordycepin on Hantaan virus 76-118 infection of primary human embryonic pulmonary fibroblasts--characterization of apoptotic effects.	https://www.ncbi.nlm.nih.gov/pubmed/16178516	y	2005	Xu FL

204	Cordycepin: selective growth inhibitor derived from liquid culture of Cordyceps militaris against Clostridium spp.	https://www.ncbi.nlm.nih.gov/pubmed/10898616	y	2000	Ahn YJ
205	Larvicidal activity against Plutella xylostella of cordycepin from the fruiting body of Cordyceps militaris.	https://www.ncbi.nlm.nih.gov/pubmed/12146173	y	2002	Kim JR
207	Effects of 3'-deoxyadenosine (cordycepin) on the repair of X-ray-induced DNA single- and double-strand breaks in Chinese hamster V79 cells.	https://www.ncbi.nlm.nih.gov/pubmed/2213687	y	2000	Hiraoka W
209	Phosphorothioate and cordycepin analogues of 2',5'-oligoadenylate: inhibition of human immunodeficiency virus type 1 reverse transcriptase and infection in vitro.	https://www.ncbi.nlm.nih.gov/pubmed/2476814	y	1989	MONTEFIORI DC
210	Identification of 6-azauridine triphosphate in L1210 cells and its possible relevance to cytotoxicity.	https://www.ncbi.nlm.nih.gov/pubmed/2463073	y	1989	Wotring LL
212	Comparison of the effects of NGF, activators of protein kinase C, and a calcium ionophore on the expression of Thy-1 and N-CAM in PC12 cell cultures.	https://www.ncbi.nlm.nih.gov/pubmed/2899083	y	1988	Doherty P
213	Factors controlling the expression of the NGF receptor in PC12 cells.	https://www.ncbi.nlm.nih.gov/pubmed/2847094	y	1988	Doherty P
214	Modulation of Na+-Pi cotransport in opossum kidney cells by extracellular phosphate.	https://www.ncbi.nlm.nih.gov/pubmed/3407761	y	1988	Biber J
215	Cordycepin rapidly collapses the intermediate filament networks into juxtanuclear caps in fibroblasts and epidermal cells.	https://www.ncbi.nlm.nih.gov/pubmed/2455649	y	1988	Zieve GW
216	Components required for in vitro cleavage and polyadenylation of eukaryotic mRNA.	https://www.ncbi.nlm.nih.gov/pubmed/2898767	y	1988	McLauchlan J
217	Metabolic effects of 3'-deoxyadenosine (cordycepin) and 2-halo-3'-deoxyadenosine on repair of X-ray-induced potentially lethal damage in Chinese hamster V79 cells.	https://www.ncbi.nlm.nih.gov/pubmed/2836881	y	1988	HIRAOKA W
218	The molecular mode of brain mRNA processing damage followed by the suppression of post-transcriptional poly(A) synthesis with cordycepin.	https://www.ncbi.nlm.nih.gov/pubmed/2265908	y	1990	KUZNETSOV DA
219	Enhanced messenger RNA stability and differentiation of HL 60 cells treated with 1,25-dihydroxyvitamin D3 and cordycepin.	https://www.ncbi.nlm.nih.gov/pubmed/2526134	y	1989	MATHEW PA
220	Cordycepin analogues of 2',5'-oligoadenylate inhibit human immunodeficiency virus infection via inhibition of reverse transcriptase.	https://www.ncbi.nlm.nih.gov/pubmed/1705437	y	1999	Muller WE

224	Influence of protein and RNA synthesis inhibitors on methylcholanthrene-mediated induction of aryl hydrocarbon hydroxylase activity in intestinal mucosa.	https://www.ncbi.nlm.nih.gov/pubmed/2469089	y	1988	Turk WA
225	Precommitted erythroid cells enriched in cultures of suboptimally induced Friend erythroleukemia cells.	https://www.ncbi.nlm.nih.gov/pubmed/3478103	y	1987	DiMambro E
226	Isolation and characterization of DNA-dependent RNA polymerase III from Leishmania mexicana and inhibition by purine analogs.	https://www.ncbi.nlm.nih.gov/pubmed/3435122	y	1987	Nolan LL
227	Identification of a complex associated with processing and polyadenylation in vitro of herpes simplex virus type 1 thymidine kinase precursor RNA.	https://www.ncbi.nlm.nih.gov/pubmed/2823124	y	1987	Zhang F
228	Detection of mRNAs coding for translationally regulated heat-shock proteins in non-heat-shocked thymic lymphocytes.	https://www.ncbi.nlm.nih.gov/pubmed/2440866	y	1987	Colbert RA
230	Differential effects of cordycepin on the induction of sister-chromatid exchange and chromatid breaks in BALB/Mo mouse lymphocytes treated with mitomycin C.	https://www.ncbi.nlm.nih.gov/pubmed/3125403	y	1987	Majione F
231	Regulation of hepatic glucokinase gene expression. Role of carbohydrates, and glucocorticoid and thyroid hormones.	https://www.ncbi.nlm.nih.gov/pubmed/3830179	y	1987	Minderop RH
232	Translation of ferritin light and heavy subunit mRNAs is regulated by intracellular chelatable iron levels in rat hepatoma cells.	https://www.ncbi.nlm.nih.gov/pubmed/3470792	y	1987	Rogers J
233	Alpha-melanocyte-stimulating hormone regulation of tyrosinase in Cloudman S-91 mouse melanoma cell cultures.	https://www.ncbi.nlm.nih.gov/pubmed/3031058	y	1987	Fuller BB
234	Ribosomal protein phosphorylation in vivo and in vitro by vaccinia virus.	https://www.ncbi.nlm.nih.gov/pubmed/3493134	y	1987	BUENDIA B
235	Cordycepin disrupts the microtubule networks and arrests Nil 8 hamster fibroblasts at the onset of mitosis.	https://www.ncbi.nlm.nih.gov/pubmed/3301008	y	1987	Zieve GW
236	The synergism of nucleoside antibiotics combined with guanine 7-N-oxide against a rhabdovirus, infectious hematopoietic necrosis virus (IHNV).	https://www.ncbi.nlm.nih.gov/pubmed/3781928	y	1986	Hasobe M
237	Cooperative effect of thyroid and glucocorticoid hormones on the induction of hepatic phosphoenolpyruvate carboxykinase in vivo and in cultured hepatocytes.	https://www.ncbi.nlm.nih.gov/pubmed/3019691	y	1986	Hoppner W

239	Mutagenic potential of cordycepin (3'-deoxyadenosine) in salmonella and soybean tester strains.	https://www.ncbi.nlm.nih.gov/pubmed/3523234	y	1986	Inoue T
241	The relation of RNA synthesis to chondroitin sulphate biosynthesis in cultured bovine cartilage.	https://www.ncbi.nlm.nih.gov/pubmed/2427073	y	1986	McQUILLAN DJ
245	Acute stimulation by glucocorticoids of gluconeogenesis from lactate/pyruvate in isolated hepatocytes from normal and adrenalectomized rats.	https://www.ncbi.nlm.nih.gov/pubmed/3876337	y	1985	Sistare FD
246	Gene expression during terminal differentiation: dexamethasone suppression of inducer-mediated alpha 1- and beta maj-globin gene expression.	https://www.ncbi.nlm.nih.gov/pubmed/3860841	y	1985	KANEDA T
248	Early heat shock proteins in primary thymocytes. Evidence for transcriptional and translational regulation.	https://www.ncbi.nlm.nih.gov/pubmed/3871771	y	1985	Maytin EV
249	Cytoplasmic microtubules are essential for the formation of membrane-bound polyribosomes.	https://www.ncbi.nlm.nih.gov/pubmed/2857170	y	1985	Walker PR
251	Cordycepin reduces the sensitivity of BALB/Mo mouse lymphocytes to the induction of sister chromatid exchanges.	https://www.ncbi.nlm.nih.gov/pubmed/3917867	y	1985	Majone F
253	Glucose-dependent induction of acetyl-CoA carboxylase in rat hepatocyte cultures.	https://www.ncbi.nlm.nih.gov/pubmed/6148072	y	1984	GIFFHORN S
254	Macromolecular and cell cycle effects of different classes of agents inducing the maturation of human myeloblastic leukemia (ML-1) cells.	https://www.ncbi.nlm.nih.gov/pubmed/6586286	y	1984	Craig RW
255	Inducer-mediated commitment of murine erythroleukemia cells to terminal cell division: the expression of commitment.	https://www.ncbi.nlm.nih.gov/pubmed/6203120	y	1984	Murate T
257	Rapid action of insulin and cyclic AMP in the regulation of functional messenger RNA coding for glucokinase in rat liver.	https://www.ncbi23405.nlm.nih.gov/pubmed/63	y	1984	Sibrowsk W
258	Induction of differentiation of the human histiocytic lymphoma cell line U-937 by 1 alpha,25-dihydroxycholecalciferol.	https://www.ncbi.nlm.nih.gov/pubmed/6315218	y	1983	Olsson I

259	Pretranslational control of tyrosine aminotransferase synthesis by 8-bromo-cyclic AMP in H-4 rat hepatoma cells.	https://www.ncbi.nlm.nih.gov/pubmed/6196356	y	1983	Culpepper JA
262	Flow cytometric analysis of adenosine analogue lymphocytotoxicity.	https://www.ncbi.nlm.nih.gov/pubmed/6577944	y	1983	Kefford RF
264	Inhibition of protein synthesis by the cordycepin analog of (2'-5')ppp(Ap)nA, (2'-5')ppp(3'dAp)n3'dA, in intact mammalian cells.	https://www.ncbi.nlm.nih.gov/pubmed/6862016	y	1983	Lee C
267	Characterization of an ATPase/dATPase activity associated with the Drosophila nuclear matrix-pore complex-lamina fraction. Identification of the putative enzyme polypeptide by direct ultraviolet photoaffinity labeling	https://www.ncbi.nlm.nih.gov/pubmed/6131895	y	1983	Berrios M
269	Time-dependent effects of alpha-amanitin on nuclear maturation and protein synthesis in mammalian oocytes.	https://www.ncbi.nlm.nih.gov/pubmed/6192196	y	1983	Osborn JC
270	Degradation of short-lived proteins is decreased by centrifugation.	https://www.ncbi.nlm.nih.gov/pubmed/6984395	y	1982	Knecht E
272	Induction of lipogenic enzymes in primary cultures of rat hepatocytes. Relationship between lipogenesis and carbohydrate metabolism.	https://www.ncbi.nlm.nih.gov/pubmed/6293823	y	1982	Spence JT
273	Utilization by Saccharomyces cerevisiae of 5'-methylthioadenosine as a source of both purine and methionine.	https://www.ncbi.nlm.nih.gov/pubmed/7045086	y	1982	Cone MC
276	Glucocorticoid-induced proteins in rat thymus cells.	https://www.ncbi.nlm.nih.gov/pubmed/6974733	y	1981	Voris BP
278	Isoleucyl-tRNA synthetase from Baker's yeast. Action of ATP analogs in pyrophosphate exchange and aminoacylation, two pathways of the aminoacylation depending on concentration of pyrophosphate.	https://www.ncbi.nlm.nih.gov/pubmed/6281001	y	1981	FREIST W
279	Inhibitory effects of 3'deoxycytidine 5'-triphosphate and 3'-deoxyuridine 5'-triphosphate on DNA-dependent RNA polymerases I and II purified from Dictyostelium discoideum cells.	https://www.ncbi.nlm.nih.gov/pubmed/7279664	y	1981	Saneyoshi M
280	Synthesis and turnover of mitochondrial ribonucleic acid in HeLa cells: the mature ribosomal and messenger ribonucleic acid species are metabolically unstable.	https://www.ncbi.nlm.nih.gov/pubmed/6086013	y	1981	GELFAND R

281	Nucleotide sequence at the termini of the DNA of Bacillus subtilis phage phi 29.	https://www.ncbi.nlm.nih.gov/pubmed/6262800	y	1981	ESCARMIS C
282	Role of insulin, glucose, and cyclic GMP in the regulation of glucokinase in cultured hepatocytes.	https://www.ncbi.nlm.nih.gov/pubmed/6257679	y	1981	Spence JT
283	Inhibition of nucleic acid methylation by cordycepin. In vivo synthesis of S-3'-DEOXYADENOSYLMETHIONINE BY WI-L2 human lymphoblasts.	https://www.ncbi.nlm.nih.gov/pubmed/6156168	y	1980	Kredich NM
284	Haem control in experimental porphyria. The effect of haemin on the induction of delta-aminolaevulinate synthase in isolated chick-embryo liver cells.	https://www.ncbi.nlm.nih.gov/pubmed/7470035	y	1980	SRIVASTAVA G
286	Nucleoside conformation is determined by the electronegativity of the sugar substituent.	https://www.ncbi.nlm.nih.gov/pubmed/7433125	y	1980	Guschlbauer W
288	Proteins of vesicular stomatitis virus. V. Identification of a precursor to the phosphoprotein of Piry virus.	https://www.ncbi.nlm.nih.gov/pubmed/225529	y	1979	Bell JC
289	Host range restriction of vaccinia virus in Chinese hamster ovary cells: relationship to shutoff of protein synthesis.	https://www.ncbi.nlm.nih.gov/pubmed/310474	y	1978	DRILLIEN R
290	Induction of ornithine decarboxylase activity in a temperature-sensitive cell cycle mutant of Chinese hamster cells.	https://www.ncbi.nlm.nih.gov/pubmed/283409	y	1978	OTSUKA FL
291	Erythro-9-(2-hydroxy-3-nonyl)adenine as a specific inhibitor of herpes simplex virus replication in the presence and absence of adenosine analogues.	https://www.ncbi.nlm.nih.gov/pubmed/216993	y	1978	North TW
293	Trypanocidal activity of antitumor antibiotics and other metabolic inhibitors.	https://www.ncbi.nlm.nih.gov/pubmed/666298	y	1978	WILLIAMSON J
294	Induction of a biopolyester hydrolase (cutinase) by low levels of cutin monomers in Fusarium solani f.sp. pisi.	https://www.ncbi.nlm.nih.gov/pubmed/415052	y	1978	LIN TS
296	Control of zinc-thionein synthesis in rat liver.	https://www.ncbi.nlm.nih.gov/pubmed/301740	y	1977	SQUIBB KS
297	Proton magnetic resonance studies of 2'-,3'-, and 5'-deoxyadenosine conformations in solution.	https://www.ncbi.nlm.nih.gov/pubmed/301272	y	1977	Westhof E
300	Adenosine utilization in cordycepin-sensitive mutants of Saccharomyces cerevisiae.	https://www.ncbi.nlm.nih.gov/pubmed/789363	y	1976	Anderson JM

301	Mitogen receptors in chick embryo fibroblasts. Kinetics, specificity, unmasking, and synthesis of 125I-insulin binding sites.	https://www.ncbi.nlm.nih.gov/pubmed/988022	y	1976	Raizada MK
303	Infulence of hormones and medium composition on the degradation of phosphoenolpyruvate carboxykinase (GTP) and total protein in Reuber H35 cells.	https://www.ncbi.nlm.nih.gov/pubmed/180001	y	1976	Gunn JM
305	Novel mechanism for translational control in regulation of ferritin synthesis by iron.	https://www.ncbi.nlm.nih.gov/pubmed/1083028	y	1976	ZAHRINGER J
307	Stimulation by aldosterone of the sodium efflux in barnacle muscle fibres: effects of RNA inhibitors and spironolactone.	https://www.ncbi.nlm.nih.gov/pubmed/1080804	y	1975	BITTAR EE
308	Synthesis of reovirus-specific polypeptides in cells pretreated with cycloheximide.	https://www.ncbi.nlm.nih.gov/pubmed/1080523	y	1975	Lau RY
309	Effect of cordycepin and cycloheximide on the induction of phosphoenolpyruvate carboxykinase by dexamethasone or N6, O2'-dibutyrylcyclic AMP in the isolated perfused rat liver.	https://www.ncbi.nlm.nih.gov/pubmed/170132	y	1975	KRONE W
310	The regulation of phosphoenolpyruvate carboxykinase (GTP) synthesis in rat kidney cortex. The role of acid-base balance and glucocorticoids.	https://www.ncbi.nlm.nih.gov/pubmed/167019	y	1975	IYNEDJIAN PB
311	Induction of the polyamine-biosynthetic enzymes in mouse epidermis by tumor-promoting agents.	https://www.ncbi.nlm.nih.gov/pubmed/48421	y	1975	O'Brien TG
313	Deinduction of phosphoenolpyruvate carboxykinase (guanosine triphosphate) synthesis in Reuber H-35 cells.	https://www.ncbi.nlm.nih.gov/pubmed/164466	y	1975	TILGHMAN SM
317	Increased rate of acetylcholinesterase synthesis in differentiating neuroblastoma cells.	https://www.ncbi.nlm.nih.gov/pubmed/4548065	y	1974	LANKS KW
319	Mechanism of stimulation of murine type-C RNA tumor virus production by glucocorticoids: post-transcriptional effects.	https://www.ncbi.nlm.nih.gov/pubmed/4370654	y	1974	Wu AM
320	Evidence for the genetic control of the sodium pump density in HeLa cells.	https://www.ncbi.nlm.nih.gov/pubmed/4279985	y	1974	BOARDMAN L
322	Spontaneous induction of endogenous murine leukemia virus-related antigen expression during short-term in vitro incubation of mouse lymphocytes.	https://www.ncbi.nlm.nih.gov/pubmed/4365582	y	1974	Lonai P
323	Rapid loss of translatable messenger RNA of phosphoenolpyruvate carboxykinase during glucose repression in liver.	https://www.ncbi.nlm.nih.gov/pubmed/4364533	y	1974	TILGHMAN SM

325	Stabilization of interferon messenger RNA activity by treatment of cells with metabolic inhibitors and lowering of the incubation temperature.	https://www.ncbi.nlm.nih.gov/pubmed/4544057	y	1973	VILCEK J
326	Inhibition of hepatic deoxyribonucleic acid-dependent ribonucleic acid polymerases by the exotoxin of Bacillus thuringiensis in comparison with the effects of -amanitin and cordycepin.	https://www.ncbi.nlm.nih.gov/pubmed/4539593	y	1972	SMUCKLER EA
327	Further experiments with the nucleoside trypanocide, cordycepin.	https://www.ncbi.nlm.nih.gov/pubmed/4558831	y	1972	WILLIAMSON J
328	The effects of cordycepin on malaria parasites.	https://www.ncbi.nlm.nih.gov/pubmed/4999656	y	1971	TRIGG PI
331	Inhibition of Ehrlich mouse ascites tumor growth by cordycepin.	https://www.ncbi.nlm.nih.gov/pubmed/13789180	y	1960	JAGGER DV
336	Systemic Morphine Produces Dose-dependent Nociceptor-mediated Biphasic Changes in Nociceptive Threshold and Neuroplasticity.	https://www.ncbi.nlm.nih.gov/pubmed/30529265	y	2019	Ferrari LF
339	Dynamic Analysis of Nucleosides and Carbohydrates during Developmental Stages of Cordyceps militaris in Silkworm (Bombyxmori).	https://www.ncbi.nlm.nih.gov/pubmed/30442223	y	2018	Wang LY
366	Cordycepin promotes apoptosis in renal carcinoma cells by activating the MKK7-JNK signaling pathway through inhibition of c-FLIPL expression.	https://www.ncbi.nlm.nih.gov/pubmed/29045468	y	2017	Hwang, I.H.
367	Apoptotic Effects of Cordycepin Through the Extrinsic Pathway and p38 MAPK Activation in Human Glioblastoma U87MG Cells.	https://www.ncbi.nlm.nih.gov/pubmed/26597532	y	2016	Baik, J.S.
368	Cordycepin increases sensitivity of Hep3B human hepatocellular carcinoma cells to TRAIL-mediated apoptosis by inactivating the JNK signaling pathway.	https://www.ncbi.nlm.nih.gov/pubmed/23828231	y	2013	Lee, H.H.
369	Transcription inhibitors stimulate translation of 5' TOP mRNAs through activation of S6 kinase and the mTOR/FRAP signalling pathway.	https://www.ncbi.nlm.nih.gov/pubmed/11054111	y	2000	Loreni, F.
370	Involvement of Ras/MAP kinase in the regulation of Ca2+ channels in adult bullfrog sympathetic neurons by nerve growth factor.	https://www.ncbi.nlm.nih.gov/pubmed/9744944	y	1998	Lei, S.
371	Formation of mRNA 3' termini: stability and dissociation of a complex involving the AAUAAA sequence.	https://pubmed.ncbi.nlm.nih.gov/2438129/	y	1987	Zarkower, D.
373	Effects of matrix metalloproteinase 13 on vascular smooth muscle cells migration via Akt-ERK dependent pathway.	https://www.ncbi.nlm.nih.gov/pubmed/25595313	y	2015	Yang, S.W.
374	SDF1-CXCR4 Signaling Contributes to the Transition from Acute to Chronic Pain State.	https://www.ncbi.nlm.nih.gov/pubmed/27011380	y	2017	Yang, F.

375	Cordycepin, a Natural Antineoplastic Agent, Induces Apoptosis of Breast Cancer Cells via Caspase-dependent Pathways.	https://www.ncbi.nlm.nih.gov/pubmed/26996021	y	2016	Wang, D.
376	A direct protein kinase B-targeted anti-inflammatory activity of cordycepin from artificially cultured fruit body of Cordyceps militaris.	https://www.ncbi.nlm.nih.gov/pubmed/26246722	y	2015	Yoon, J.Y.
377	Intervention in genotoxic stress-induced senescence by cordycepin through activation of eIF2α and suppression of Sp1.	https://www.ncbi.nlm.nih.gov/pubmed/23690541	y	2013	Gu, L.
379	In vitro maturation of bovine oocytes requires polyadenylation of mRNAs coding proteins for chromatin condensation, spindle assembly, MPF and MAP kinase activation	https://www.ncbi.nlm.nih.gov/pubmed/12363437	y	2002	Krischek, C.
382	Fabrication of 3D calcium-alginate scaffolds for human glioblastoma modeling and anticancer drug response evaluation.	https://www.ncbi.nlm.nih.gov/pubmed/30945284	y	2019	Chaicharoenaudomrung N
384	Ethylene carbodiimide-fixed donor splenocytes combined with cordycepin induce long-term protection to mice cardiac allografts.	https://www.ncbi.nlm.nih.gov/pubmed/30743003	y	2019	Xingqiang L
385	Breeding of a cordycepin-resistant and adenosine kinase-deficient sake yeast strain that accumulates high levels of S-adenosylmethionine.	https://www.ncbi.nlm.nih.gov/pubmed/30686113	y	2019	Kanai M
387	Mu-opioid Receptor (MOR) Biased Agonists Induce Biphasic Dose-dependent Hyperalgesia and Analgesia, and Hyperalgesic Priming in the Rat.	https://www.ncbi.nlm.nih.gov/pubmed/30342200	y	2018	Araldi D
390	Suppression of Cell Growth, Migration and Drug Resistance by Ethanolic Extract of Antrodia cinnamomea in Human Lung Cancer A549 Cells and C57BL/6J Allograft Tumor Model.	https://www.ncbi.nlm.nih.gov/pubmed/29522490	y	2018	Wu CH
391	Cordycepin, a Characteristic Bioactive Constituent in Cordyceps militaris, Ameliorates Hyperuricemia through URAT1 in Hyperuricemic Mice.	https://www.ncbi.nlm.nih.gov/pubmed/29422889	y	2018	Yong T
393	Accumulation of intracellular S-adenosylmethionine increases the fermentation rate of bottom-fermenting brewer's yeast during high-gravity brewing.	https://www.ncbi.nlm.nih.gov/pubmed/29921531	y	2018	Oomuro M
394	Discovery of Novel 7-Aryl 7-Deazapurine 3'-Deoxy-ribofuranosyl Nucleosides with Potent Activity against Trypanosoma cruzi.	https://www.ncbi.nlm.nih.gov/pubmed/30234983	y	2018	Hulpia F
398	The inhibition of cordycepin on cancer stemness in TGF-beta induced chemo-resistant ovarian cancer cell.	https://www.ncbi.nlm.nih.gov/pubmed/29340100	y	2017	Wang CW

407	Effects of N-Nitrosodiethylamine, a Potent Carcinogen, on Sexual Development, Gametogenesis, and Oocyte Maturation.	https://www.ncbi.nlm.nih.gov/pubmed/28662518	y	2017	NairU R
408	Higher Anti-Liver Fibrosis Effect of Cordyceps militaris-Fermented Product Cultured with Deep Ocean Water via Inhibiting Proinflammatory Factors and Fibrosis-Related Factors Expressions.	https://www.ncbi.nlm.nih.gov/pubmed/28594374	y	2017	Hung YP
409	Crystallographic and SAXS studies of S-adenosyl-l-homocysteine hydrolase from Bradyrhizobium elkanii.	https://www.ncbi.nlm.nih.gov/pubmed/28512574	y	2017	Manszewski T
416	Cordycepin Downregulates Cdk-2 to Interfere with Cell Cycle and Increases Apoptosis by Generating ROS in Cervical Cancer Cells: in vitro and in silico Study.	https://www.ncbi.nlm.nih.gov/pubmed/30182857	y	2019	Tania M
418	Cordycepin Modulates Body Weight by Reducing Prolactin Via an Adenosine A1 Receptor.	https://www.ncbi.nlm.nih.gov/pubmed/30124145	y	2018	Li Y
424	Cytotoxic compounds against cancer cells from Bombyx mori inoculated with Cordyceps militaris.	https://www.ncbi.nlm.nih.gov/pubmed/28485210	y	2017	Qiu W
430	Poly(ADP-ribose) polymerases covalently modify strand break termini in DNA fragments in vitro.	https://www.ncbi.nlm.nih.gov/pubmed/27471034	y	2016	Talhaoui I
432	The Effects of Polyadenylation Status on MPFs During In Vitro Porcine Oocyte Maturation.	https://www.ncbi.nlm.nih.gov/pubmed/27744448	y	2016	Liu H
434	Separation of cordycepin from Cordyceps militaris fermentation supernatant using preparative HPLC and evaluation of its antibacterial activity as an NAD(+)-dependent DNA ligase inhibitor.	https://www.ncbi.nlm.nih.gov/pubmed/27588098	y	2016	ZHOU X
436	Preparative isolation of cordycepin, N(6)-(2-hydroxyethyl)-adenosine and adenosine from Cordyceps militaris by macroporous resin and purification by recycling high-speed counter-current chromatography.	https://www.ncbi.nlm.nih.gov/pubmed/27567378	y	2016	Zhang Z
442	Cordycepin Affects Multiple Apoptotic Pathways to Mediate Hepatocellular Carcinoma Cell Death.	https://www.ncbi.nlm.nih.gov/pubmed/27225448	y	2017	Zhou Y
443	Gi-protein-coupled 5-HT1B/D receptor agonist sumatriptan induces type I hyperalgesic priming.	https://www.ncbi.nlm.nih.gov/pubmed/27075428	y	2016	Araldi D
444	Trypanosoma brucei Methylthioadenosine Phosphorylase Protects the Parasite from the Antitrypanosomal Effect of Deoxyadenosine: IMPLICATIONS FOR THE PHARMACOLOGY OF ADENOSINE ANTIMETABOLITES.	https://www.ncbi.nlm.nih.gov/pubmed/27036940	y	2016	Vodnala M

456	N(6)-(2-Hydroxyethyl)adenosine in the Medicinal Mushroom Cordyceps cicadae Attenuates Lipopolysaccharide-Stimulated Pro-inflammatory Responses by Suppressing TLR4-Mediated NF-îºB Signaling Pathways.	https://www.ncbi.nlm.nih.gov/pubmed/26394068	y	2015	Lu MY
458	Systematic analysis of the contribution of c-myc mRNA constituents upon cap and IRES mediated translation.	https://www.ncbi.nlm.nih.gov/pubmed/26351916	y	2015	Meristoudis C
464	Inhibition of adenosine deaminase (ADA)-mediated metabolism of cordycepin by natural substances.	https://www.ncbi.nlm.nih.gov/pubmed/26038697	y	2014	Li G
465	Dose finding of 3'deoxyadenosine and deoxycoformycin for the treatment of Trypanosoma evansi infection: An effective and nontoxic dose.	https://www.ncbi.nlm.nih.gov/pubmed/26025154	y	2015	Rosa LD
470	Toxicity evaluation of cordycepin and its delivery system for sustained in vitro anti-lung cancer activity.	https://www.ncbi.nlm.nih.gov/pubmed/25883541	y	2015	Aramwit P
471	Cordycepin and N6-(2-hydroxyethyl)-adenosine from Cordyceps pruinosa and their interaction with human serum albumin.	https://www.ncbi.nlm.nih.gov/pubmed/25811172	y	2015	Meng Z
475	Preparation, spectroscopy and molecular modelling studies of the inclusion complex of cordycepin with cyclodextrins.	https://www.ncbi.nlm.nih.gov/pubmed/25679304	y	2015	Zhang JQ
477	The Lingulodinium circadian system lacks rhythmic changes in transcript abundance.	https://www.ncbi.nlm.nih.gov/pubmed/25526979	y	2014	Roy S
482	Proteasome regulates the mediators of cytoplasmic polyadenylation signaling during late-phase long-term potentiation.	https://www.ncbi.nlm.nih.gov/pubmed/25263789	y	2014	Dong C
487	3'-End labeling of RNA with yeast Poly(A) polymerase and 3'-deoxyadenosine 5'-[Î±-32P]triphosphate.	https://www.ncbi.nlm.nih.gov/pubmed/24890209	y (PROTOCOL)	2014	Rio DC
489	Anti-HCV activity of the Chinese medicinal fungus Cordyceps militaris.	https://www.ncbi.nlm.nih.gov/pubmed/24726408	y	2014	Ueda Y
493	Structures of adenosine kinase from Trypanosoma brucei brucei.	https://www.ncbi.nlm.nih.gov/pubmed/24419613	y	2014	Timm J
496	The human nuclear poly(a)-binding protein promotes RNA hyperadenylation and decay.	https://www.ncbi.nlm.nih.gov/pubmed/24146636	y	2013	Bresson SM
499	Crystal structure of human poly(A) polymerase gamma reveals a conserved catalytic core for canonical poly(A) polymerases.	https://www.ncbi.nlm.nih.gov/pubmed/24076191	y	2014	Yang Q
502	Anti-cancer effect and apoptosis induction of cordycepin through DR3 pathway in the human colonic cancer cell HT-29.	https://www.ncbi.nlm.nih.gov/pubmed/23941773	y	2013	Lee SY

503	Influence of treatment with 3'-deoxyadenosine associated deoxycoformycin on hematological parameters and activity of adenosine deaminase in infected mice with Trypanosoma evansi.	https://www.ncbi.nlm.nih.gov/pubmed/23933282	y	2013	Rosa LD
504	Suppression of T-cell activation inÂ vitro and inÂ vivo byÂ cordycepin from Cordyceps militaris.	https://www.ncbi.nlm.nih.gov/pubmed/23927879	y	2013	Xiong Y
507	Cordycepin suppresses integrin/FAK signaling and epithelial-mesenchymal transition in hepatocellular carcinoma.	https://www.ncbi.nlm.nih.gov/pubmed/23855336	y	2014	Yao WL
512	Apoptotic effect of cisplatin and cordycepin on OC3 human oral cancer cells.	https://www.ncbi.nlm.nih.gov/pubmed/23543357	y	2013	Chen YH
515	Cordycepin suppresses excitatory synaptic transmission in rat hippocampal slices via a presynaptic mechanism.	https://www.ncbi.nlm.nih.gov/pubmed/23419191	y	2012	Yao LH
516	Cordycepin (3'-deoxyadenosine) pentostatin (deoxycoformycin) combination treatment of mice experimentally infected with Trypanosoma evansi.	https://www.ncbi.nlm.nih.gov/pubmed/23361035	y	2013	Rosa LD
517	Apoptosis induction of human prostate carcinoma cells by cordycepin through reactive oxygen speciesâ€'mediated mitochondrial death pathway.	https://www.ncbi.nlm.nih.gov/pubmed/23292300	y	2013	Lee HH
524	mRNAs containing the histone 3' stem-loop are degraded primarily by decapping mediated by oligouridylation of the 3' end.	https://www.ncbi.nlm.nih.gov/pubmed/23188809	y	2013	Su W
525	The nucleoside antagonist cordycepin causes DNA double strand breaks in breast cancer cells.	https://www.ncbi.nlm.nih.gov/pubmed/22821173	y	2012	Lee HJ
526	Cordycepin induces apoptosis in human neuroblastoma SK-N-BE(2)-C and melanoma SK-MEL-2 cells.	https://www.ncbi.nlm.nih.gov/pubmed/22650004	y	2012	Baik JS
532	Anti-adipogenic activity of Cordyceps militaris in 3T3-L1 cells.	https://www.ncbi.nlm.nih.gov/pubmed/22312720	y	2011	Liu Q
535	Protective effect of Cordyceps militaris against high glucose-induced oxidative stress in human umbilical vein endothelial cells.	https://www.ncbi.nlm.nih.gov/pubmed/25212312	y	2012	Chu HL
538	Lipid-lowering effect of cordycepin (3'-deoxyadenosine) from Cordyceps militaris on hyperlipidemic hamsters and rats.	https://www.ncbi.nlm.nih.gov/pubmed/21882527	y	2011	Gao J
540	Nucleophosmin deposition during mRNA 3' end processing influences poly(A) tail length.	https://www.ncbi.nlm.nih.gov/pubmed/21822216	y	2011	Sagawa F
543	Beneficial effects of cordycepin on metabolic profiles of liver and plasma from hyperlipidemic hamsters.	https://www.ncbi.nlm.nih.gov/pubmed/21623517	y	2011	Sun Y

545	Evidence for an RNA polymerization activity in axolotl and Xenopus egg extracts.	https://www.ncbi.nlm.nih.gov/pubmed/21203452	y	2011	Pelczar H
548	The role of cytoplasmic polyadenylation element sequence on mRNA abundance during porcine embryogenesis and parthenogenetic development.	https://www.ncbi.nlm.nih.gov/pubmed/20626047	y	2010	DOBBS KB
554	Involvement of polyadenylation status on maternal gene expression during in vitro maturation of porcine oocytes.	https://www.ncbi.nlm.nih.gov/pubmed/19479986	y	2009	Zhang DX
556	Cordycepin interferes with 3' end formation in yeast independently of its potential to terminate RNA chain elongation.	https://www.ncbi.nlm.nih.gov/pubmed/19324962	y	2009	HOLBEIN S
563	Proteomic detection of changes in protein expression induced by cordycepin in human hepatocellular carcinoma BEL-7402 cells.	https://www.ncbi.nlm.nih.gov/pubmed/18806893	y	2008	Shi P
564	Synthesis and properties of (alpha-P-borano)-nucleoside 5'-triphosphate analogues as potential antiviral agents.	https://www.ncbi.nlm.nih.gov/pubmed/18776263	y	2008	Cheek MA
568	Adenosine kinase mediates high affinity adenosine salvage in Trypanosoma brucei.	https://www.ncbi.nlm.nih.gov/pubmed/18167353	y	2008	Vodnala M
569	Cordycepin-hypersensitive growth links elevated polyphosphate levels to inhibition of poly(A) polymerase in Saccharomyces cerevisiae.	https://www.ncbi.nlm.nih.gov/pubmed/18033801	y	2008	Holbein S
571	Adenosine kinase of Trypanosoma brucei and its role in susceptibility to adenosine antimetabolites.	https://www.ncbi.nlm.nih.gov/pubmed/17698621	y	2007	Lu¨scher A
575	Reinforcement of antitumor effect of Cordyceps sinensis by 2'-deoxycoformycin, an adenosine deaminase inhibitor.	https://www.ncbi.nlm.nih.gov/pubmed/17436579	y	2007	Yoshikawa N
579	Cytoplasmic polyadenylation controls cdc25B mRNA translation in rat oocytes resuming meiosis.	https://www.ncbi.nlm.nih.gov/pubmed/16816330	y	2006	Gershon E
585	Cyclin A2-CDK2 regulates embryonic gene activation in 1-cell mouse embryos.	https://www.ncbi.nlm.nih.gov/pubmed/16137671	y	2005	Hara KT
587	Molecular pharmacology of adenosine transport in Trypanosoma brucei: P1/P2 revisited.	https://www.ncbi.nlm.nih.gov/pubmed/15933219	y	2005	Geiser F
588	Analysis of a 17-amino acid residue, virus-neutralizing microantibody	https://www.ncbi.nlm.nih.gov/pubmed/15914858	y	2005	Heap CJ
590	Effects of polyadenylation inhibition on meiosis progression in relation to the polyadenylation status of cyclins A2 and B1 during in vitro maturation of bovine oocytes.	https://www.ncbi.nlm.nih.gov/pubmed/15736128	y	2005	TRAVERSO JM

592	Anti-inflammatory and related pharmacological activities of cultured mycelia and fruiting bodies of Cordyceps militaris.	https://www.ncbi.nlm.nih.gov/pubmed/15619578	y	2005	Won S
595	Selection and evolution of NTP-specific aptamers	https://www.ncbi.nlm.nih.gov/pubmed/15452272	y	2004	Weill L
596	The comet assay differentiates efficiently and rapidly between genotoxins and cytotoxins in quiescent cells.	https://www.ncbi.nlm.nih.gov/pubmed/15261156	y	2004	Daza P
597	Coupled amplification and degradation of exogenous RNA injected in amphibian oocytes.	https://www.ncbi.nlm.nih.gov/pubmed/15161959	y	2004	Montreau N
598	Z-DNA, a new in situ marker for transcription.	https://www.ncbi.nlm.nih.gov/pubmed/15145775	y	2004	Cerná A
599	Poly(A)+ RNAs roam the cell nucleus and pass through speckle domains in transcriptionally active and inactive cells.	https://www.ncbi.nlm.nih.gov/pubmed/15117966	y	2004	Molenaar C
600	K562 cell sensitization to 5-fluorouracil- or interferon-alpha-induced apoptosis via cordycepin (3'-deoxyadenosine): fine control of cell apoptosis via poly(A) polymerase upregulation.	https://www.ncbi.nlm.nih.gov/pubmed/15077928	y	2004	Lallas CC
601	Insulin and prolactin synergistically stimulate beta-casein messenger ribonucleic acid translation by cytoplasmic polyadenylation.	https://www.ncbi.nlm.nih.gov/pubmed/15071091	y	2004	CHOI KM
603	The metastasis suppressor NM23-H1 possesses 3'-5' exonuclease activity.	https://www.ncbi.nlm.nih.gov/pubmed/14960567	y	2004	Ma D
604	Injection of exogenous RNA in amphibian oocytes leads to RNA level fluctuations which are sensitive to cordycepin, an RNA chain elongation terminator.	https://www.ncbi.nlm.nih.gov/pubmed/14746268	y	2003	Montreau N
608	Expression in Escherichia coli of a recombinant adenosine kinase from Saccharomyces cerevisiae: purification, kinetics and substrate analyses.	https://www.ncbi.nlm.nih.gov/pubmed/14558146	y	2003	Barrado P
609	Cordycepin in Schizosaccharomyces pombe: effects on the wild type and phenotypes of mutants resistant to the drug.	https://www.ncbi.nlm.nih.gov/pubmed/12827445	y	2003	Naula N
610	Functional characterization of a H+/nucleoside co-transporter (CaCNT) from Candida albicans, a fungal member of the concentrative nucleoside transporter (CNT) family of membrane proteins.	https://www.ncbi.nlm.nih.gov/pubmed/12794928	y	2003	Loewen SK
611	Inhibition of the replication of a hepatitis C virus-like RNA template by interferon and 3'-deoxycytidine.	https://www.ncbi.nlm.nih.gov/pubmed/12718408	y	2002	King RW
613	RNAi: mammalian oocytes do it without RNA-dependent RNA polymerase.	https://www.ncbi.nlm.nih.gov/pubmed/12554861	y	2003	Stein P
614	Acquisition of transcriptional competence in the 1-cell mouse embryo: requirement for recruitment of maternal mRNAs.	https://www.ncbi.nlm.nih.gov/pubmed/12548659	y	2003	Aoki F

617	DNA strand-breaks induced by the topoisomerase I inhibitor camptothecin in unstimulated human white blood cells.	https://www.ncbi.nlm.nih.gov/pubmed/12175674	y	2002	Daza P
618	Three-dimensional organization of active rRNA genes within the nucleolus.	https://www.ncbi.nlm.nih.gov/pubmed/12140261	y	2002	Cheutin T
620	Release of snRNP and RNA from transcription sites in adenovirus-infected cells.	https://www.ncbi.nlm.nih.gov/pubmed/12027457	y	2002	Aspegren A
621	Resumption of meiosis induced by meiosis-activating sterol has a different signal transduction pathway than spontaneous resumption of meiosis in denuded mouse oocytes cultured in vitro.	https://www.ncbi.nlm.nih.gov/pubmed/11717137	y	2001	Faerge I
624	Posttranscriptional regulation of cyclin A1 and cyclin A2 during mouse oocyte meiotic maturation and preimplantation development.	https://www.ncbi.nlm.nih.gov/pubmed/11566717	y	2001	Fuchimoto D
626	Nerve growth factor regulates sodium but not potassium channel currents in sympathetic B neurons of adult bullfrogs.	https://www.ncbi.nlm.nih.gov/pubmed/11495939	y	2001	Lei S
627	Identification and characterization of trypanocides by functional expression of an adenosine transporter from Trypanosoma brucei in yeast.	https://www.ncbi.nlm.nih.gov/pubmed/11357935	y	2001	Maser P

629	RNA polymerase III transcription complexes on chromosomal 5S rRNA genes in vivo: TFIIIB occupancy and promoter opening.	https://www.ncbi.nlm.nih.gov/pubmed/11287621	y	2001	Costanzo G
630	Role of adenosine kinase in Saccharomyces cerevisiae: identification of the ADO1 gene and study of the mutant phenotypes.	https://www.ncbi.nlm.nih.gov/pubmed/11223943	y	2001	Lecoq K
631	Differential induction of rat hepatic cytochromes P450 3A1, 3A2, 2B1, 2B2, and 2E1 in response to pyridine treatment.	https://www.ncbi.nlm.nih.gov/pubmed/11181506	y	2001	Kim H
632	Combination therapy with purine nucleoside analogs.	https://www.ncbi.nlm.nih.gov/pubmed/10887642	y	2000	Foss FM
633	Coupling ribose selection to fidelity of DNA synthesis. The role of Tyr-115 of human immunodeficiency virus type 1 reverse transcriptase.	https://www.ncbi.nlm.nih.gov/pubmed/10748215	y	2000	Cases-Gonza´lez CE
634	Translational regulation of cyclin B mRNA by 17alpha,20beta-dihydroxy-4-pregnen-3-one (maturation-inducing hormone) during oocyte maturation in a teleost fish, the goldfish (Carassius auratus).	https://www.ncbi.nlm.nih.gov/pubmed/10630408	y	1999	Katsu Y
635	De novo initiation of RNA synthesis by the RNA-dependent RNA polymerase (NS5B) of hepatitis C virus.	https://www.ncbi.nlm.nih.gov/pubmed/10623748	y	2000	Luo G

636	Regulatory features of transcription in isolated mitochondria from Artemia franciscana embryos.	https://www.ncbi.nlm.nih.gov/pubmed/10600903	y	1999	Eads BD
637	Association of pS2 (TFF1) release with breast tumour proliferative rate: in vitro and in vivo studies.	https://www.ncbi.nlm.nih.gov/pubmed/10535357	y	1999	Reshkin SJ
638	Virtual combinatorial syntheses and computational screening of new potential anti-herpes compounds.	https://www.ncbi.nlm.nih.gov/pubmed/10464017	y	1999	Julia´n-Ortiz JV
639	Expression of the 72-kD heat shock protein is induced by ultraviolet A radiation in a human fibrosarcoma cell line.	https://www.ncbi.nlm.nih.gov/pubmed/10389635	y	1999	Trautinger F
640	Determinants of nucleotide sugar recognition in an archaeon DNA polymerase.	https://www.ncbi.nlm.nih.gov/pubmed/10352184	y	1999	Gardner AF
641	Inhibitory effect of Cordyceps sinensis on spontaneous liver metastasis of Lewis lung carcinoma and B16 melanoma cells in syngeneic mice.	https://www.ncbi.nlm.nih.gov/pubmed/10230862	y	1999	Nakamura K
644	Structurally altered substrates for DNA topoisomerase I. Effects of inclusion of a single 3'-deoxynucleotide within the scissile strand.	https://www.ncbi.nlm.nih.gov/pubmed/9708360	y	1998	Arslan T
645	Meiotic maturation in Xenopus requires polyadenylation of multiple mRNAs.	https://www.ncbi.nlm.nih.gov/pubmed/9606198	y	1998	Barkoff A
648	17 alpha,20 beta-dihydroxy-4-pregnen-3-one, a maturation-inducing hormone in fish oocytes: mechanisms of synthesis and action.	https://www.ncbi.nlm.nih.gov/pubmed/9029736	y	1997	Nagahama Y
649	Evidence for a central role of transcription in the timing mechanism of a circadian clock.	https://www.ncbi.nlm.nih.gov/pubmed/8944648	y	1996	Khalsa SB
650	Inhibition of the human immunodeficiency virus type 1 integrase by guanosine quartet structures.	https://www.ncbi.nlm.nih.gov/pubmed/8901518	y	1996	Mazumder A
651	Oxygen-induced apoptosis in PC12 cells with special reference to the role of Bcl-2.	https://www.ncbi.nlm.nih.gov/pubmed/8891300	y	1996	Kubo T

652	3'-Deoxyribonucleotides inhibit eukaryotic DNA primase.	https://www.ncbi.nlm.nih.gov/pubmed/8827435	y	1996	Izuta S
653	Induction of apoptosis by cordycepin in ADA-inhibited TdT-positive leukemia cells.	https://www.ncbi.nlm.nih.gov/pubmed/8667637	y	1996	Koc Y
655	Analyses of the first chemical step in Flp site-specific recombination: Synapsis may not be a pre-requisite for strand cleavage.	https://www.ncbi.nlm.nih.gov/pubmed/8642593	y	1996	Voziyanov Y
657	Cloning of the mink plasminogen activator inhibitor type-1 messenger RNA: an mRNA with a short half life.	https://www.ncbi.nlm.nih.gov/pubmed/7557448	y	1995	Chuang TH
658	Ecdysone Regulated RNA synthesis in Drosophila larval salivary glands.	https://www.ncbi.nlm.nih.gov/pubmed/10993979	y	1995	Farkas R
661	Adenosine kinase-deficient mutant of Saccharomyces cerevisiae.	https://www.ncbi.nlm.nih.gov/pubmed/7737480	y	1995	Iwashima A
662	In vitro RNA editing-like activity in a mitochondrial extract from Leishmania tarentolae.	https://www.ncbi.nlm.nih.gov/pubmed/7828590	y	1995	Frech GC
665	2',3'-Dideoxyadenosine killing of TdT-positive cells is due to a trace contaminant.	https://www.ncbi.nlm.nih.gov/pubmed/7845029	y	1995	Koc Y
666	Synergistic and additive combinations of several antitumor drugs and other agents with the potent alkylating agent adozelesin.	https://www.ncbi.nlm.nih.gov/pubmed/7533669	y	1995	Smith KS
667	Formation of sea urchin primary mesenchyme: cell shape changes are independent of epithelial detachment.	https://www.ncbi.nlm.nih.gov/pubmed/28305938	y	1994	Anstrom JA
668	Regulation of tyrosine aminotransferase activity by glucagon and cAMP analogues in chick embryos in ovo.	https://www.ncbi.nlm.nih.gov/pubmed/7894891	y	1994	Onoagbe IO
669	Methylphosphonodiester substitution near the conserved CA dinucleotide in the HIV LTR alters both extent of 3'-processing and choice of nucleophile by HIV-1 integrase.	https://www.ncbi.nlm.nih.gov/pubmed/7971274	y	1994	Mazumder A

671	Heat shock modulates UVB-induced cell death in human epidermal keratinocytes: evidence for a hyperthermia-inducible protective response.	https://www.ncbi.nlm.nih.gov/pubmed/7930680	y	1994	Maytin EV
673	Stearoyl-CoA desaturase activity in primary culture of chicken hepatocytes. Influence of insulin, glucocorticoid, fatty acids and cordycepin.	https://www.ncbi.nlm.nih.gov/pubmed/7914877	y	1994	LEGRAND P
674	Effects of glucocorticosteroids and insulin on tyrosine aminotransferase activity in isolated chick embryo hepatocytes and in intact embryos in ovo.	https://www.ncbi.nlm.nih.gov/pubmed/7906936	y	1994	Onoagbe IO
675	Quantitative determination of Trypanosoma cruzi growth inside host cells in vitro and effect of allopurinol.	https://www.ncbi.nlm.nih.gov/pubmed/7660956	y	1994	Aoki T
676	The effect of diamphenethide on protein synthesis by the liver fluke, Fasciola hepatica.	https://www.ncbi.nlm.nih.gov/pubmed/7507903	y	1993	ANDERSON HR
677	The glucagon-insulin antagonism in the regulation of cytosolic protein binding to the 3' end of phosphoenolpyruvate carboxykinase mRNA in cultured rat hepatocytes. Possible involvement in the stabilization of the mRNA [illegible]	https://www.ncbi.nlm.nih.gov/pubmed/8354260	y	1993	Christ B
678	3'-end labeling of RNA with recombinant yeast poly(A) polymerase.	https://www.ncbi.nlm.nih.gov/pubmed/7687347	y	1993	Lingner J
682	Differential effects of heat shock and UVB light upon stress protein expression in epidermal keratinocytes.	https://www.ncbi.nlm.nih.gov/pubmed/1429666	y	1992	Maytin EV
683	The effect of topoisomerase inhibitors on the expression of differentiation markers and cell cycle progression in human K-562 leukemia cells.	https://www.ncbi.nlm.nih.gov/pubmed/1330653	y	1992	Constantinou A
684	Effect of thiamin on cordycepin sensitivity in Saccharomyces cerevisiae.	https://www.ncbi.nlm.nih.gov/pubmed/1397293	y	1992	Iwashima A
685	Analysis of the inhibition of commitment of murine erythroleukemia (MEL) cells to terminal maturation by N6-methyladenosine.	https://www.ncbi.nlm.nih.gov/pubmed/1530661	y	1992	Vizirianakis IS
686	Maturation of Xenopus laevis oocyte by progesterone requires poly(A) tail elongation of mRNA.	https://www.ncbi.nlm.nih.gov/pubmed/1355048	y	1992	Kuge H

687	Dual action of phosphonoformic acid on Na(+)-phosphate cotransport in opossum kidney cells.	https://www.ncbi.nlm.nih.gov/pubmed/1380774	y	1992	Adham ML
688	An inhibitor of potentially lethal damage (PLD) repair reduces the frequency of gamma-ray-induced mutations in cultured Chinese hamster V79 cells.	https://www.ncbi.nlm.nih.gov/pubmed/1379331	y	1992	Yokoiyama A
689	Effects of glucagon and an analogue of cAMP on tyrosine aminotransferase in isolated chick embryo hepatocytes.	https://www.ncbi.nlm.nih.gov/pubmed/1353457	y	1992	Onoagbe IO
690	Inhibition of human immunodeficiency virus type 1 reverse transcriptase by 3'-blocked oligonucleotide primers.	https://www.ncbi.nlm.nih.gov/pubmed/1378738	y	1992	Austermann S
692	Molecular and cellular mechanism of neuronal degeneration caused by nerve growth factor deprivation approached through PC12 cell culture.	https://www.ncbi.nlm.nih.gov/pubmed/1557511	y	1992	KOIKE T
694	Ara-ATP impairs 3'-end processing of pre-mRNAs by inhibiting both cleavage and polyadenylation.	https://www.ncbi.nlm.nih.gov/pubmed/1719481	y	1991	Ghoshal K
696	Increase of CYP1A1 mRNA and AHH activity by inhibitors of either protein or RNA synthesis in mouse hepatocytes in primary culture.	https://www.ncbi.nlm.nih.gov/pubmed/1934297	y	1991	Nemoto N
697	In vitro deadenylation of mammalian mRNA by a HeLa cell 3' exonuclease.	https://www.ncbi.nlm.nih.gov/pubmed/1717259	y	1991	Astrom J
700	Regulation of GM-CSF and IL-3 production from the murine keratinocyte cell line PAM 212 following exposure to ultraviolet radiation.	https://www.ncbi.nlm.nih.gov/pubmed/1649225	y	1991	Gallo RL
702	Expression of alkaline phosphatase in murine lymphoma cells.	https://www.ncbi.nlm.nih.gov/pubmed/1645955	y	1991	Harb J
704	The anti-progestin RU486 stabilizes the progestin-induced fatty acid synthetase mRNA but does not stimulate its transcription.	https://www.ncbi.nlm.nih.gov/pubmed/2022639	y	1991	Chalbos D
705	Antagonism between camptothecin and topoisomerase II-directed chemotherapeutic agents in a human leukemia cell line.	https://www.ncbi.nlm.nih.gov/pubmed/1705167	y	1991	Kaufmann SH

708	Hypoxic depression of mitochondrial mRNA levels in HeLa cell.	https://www.ncbi.nlm.nih.gov/pubmed/1701727	y	1991	Kadowaki T
709	Rapid induction of polyadenylate binding protein and stimulation of translational initiation in pituitary tumor cells exposed to phorbol ester.	https://www.ncbi.nlm.nih.gov/pubmed/1686594	y	1991	CADE C
710	Thermotolerance and the heat shock response in normal human keratinocytes in culture.	https://www.ncbi.nlm.nih.gov/pubmed/2250106	y	1990	Maytin EV
711	Involvement of nucleic acid synthesis in cell killing mechanisms of topoisomerase poisons.	https://www.ncbi.nlm.nih.gov/pubmed/1698546	y	1990	D'Arpa P
712	Reduction of the potent DNA polymerase III holoenzyme 3'----5' exonuclease activity by template-primer analogues.	https://www.ncbi.nlm.nih.gov/pubmed/2176842	y	1990	Griep MA
713	RNA processing in vitro produces mature 3' ends of a variety of Saccharomyces cerevisiae mRNAs.	https://www.ncbi.nlm.nih.gov/pubmed/2160581	y	1990	Butller JS
714	Mechanism of the inhibition by insulin of the glucagon-dependent activation of the phosphoenolpyruvate carboxykinase gene in rat hepatocyte cultures. Action on gene transcription, mRNA level and -stability as well as hysteresis effect.	https://www.ncbi.nlm.nih.gov/pubmed/2198886	y	1990	Christ B
717	Mobility of nucleoside transporter of human erythrocytes differs greatly when loaded with different nucleosides.	https://www.ncbi.nlm.nih.gov/pubmed/2302397	y	1990	Plagemann PG
719	Human cytomegalovirus. Stimulation of [3H] release from [3H]-arachidonic acid prelabelled cells.	https://www.ncbi.nlm.nih.gov/pubmed/2171460	y	1990	AbuBaka S
720	High extracellular calcium increases the production of a parathyroid hormone-like activity by cultured Leydig tumor cells associated with humoral hypercalcemia.	https://www.ncbi.nlm.nih.gov/pubmed/2610020	y	1990	Rizzoli R
724	Insulin-like growth factor I stimulates Na-dependent Pi transport in cultured kidney cells.	https://www.ncbi.nlm.nih.gov/pubmed/2556033	y	1989	Caverzasio J
725	Modulation of alkaline phosphatases in LoVo, a human colon carcinoma cell line.	https://www.ncbi.nlm.nih.gov/pubmed/2804087	y	1989	Herz F

727	Relationship between DNA replicon size and SCE induction in BALB/c and BALB/Mo mouse lymphocytes.	https://www.ncbi.nlm.nih.gov/pubmed/2505071	y	1989	Majone F
729	Poly(A) elongation during Xenopus oocyte maturation is required for translational recruitment and is mediated by a short sequence element.	https://www.ncbi.nlm.nih.gov/pubmed/2568313	y	1989	McGrew LL
730	A protein factor that enhances amsacrine-mediated formation of topoisomerase II-DNA complexes in murine mastocytoma cell nuclei.	https://www.ncbi.nlm.nih.gov/pubmed/2539190	y	1989	Darkin SJ
731	Progestin increases gene transcription and messenger ribonucleic acid stability of fatty acid synthetase in breast cancer cells.	https://www.ncbi.nlm.nih.gov/pubmed/2471072	y	1989	Joyeux C
732	Inhibition of protein synthesis reduces the cytotoxicity of 4'-(9-acridinylamino)methanesulfon-m-anisidide without affecting DNA breakage and DNA topoisomerase II in a murine mastocytoma cell line	https://www.ncbi.nlm.nih.gov/pubmed/2464346	y	1989	Schneider E
733	Selective toxicity of purine nucleosides to human leukaemic cells.	https://www.ncbi.nlm.nih.gov/pubmed/2514591	y	1989	Piga A
734	The effect of modulators of radiation-induced G2 arrest on the repair of radiation-induced DNA damage detectable by neutral filter elution.	https://www.ncbi.nlm.nih.gov/pubmed/2902169	y	1988	ROWLEY R
735	Vaccinia virus poly(A) polymerase. Specificity for nucleotides and nucleotide analogs.	https://www.ncbi.nlm.nih.gov/pubmed/2836422	y	1988	Shuman S
738	Regulation of translation and stability of an mRNA coding for a 40-kDa polypeptide in rat L6 muscle cells.	https://www.ncbi.nlm.nih.gov/pubmed/3350002	y	1988	Pramanik SK
741	Regulation of aortic CuZn-superoxide dismutase with copper. Caeruloplasmin and albumin re-activate and transfer copper to the enzyme in culture.	https://www.ncbi.nlm.nih.gov/pubmed/3435477	y	1987	Dameron CT
742	Erythroid differentiation of K562 cells: mixed colonies as an index of delayed expression of commitment.	https://www.ncbi.nlm.nih.gov/pubmed/3305052	y	1987	Ohlsson-Wilhelm BM
743	The role of cAMP and calcium in the stimulation of proliferation of immature erythroblasts by erythropoietin.	https://www.ncbi.nlm.nih.gov/pubmed/3036549	y	1987	BONANOU-TZEDAKI SA

744	Products of in vitro cleavage and polyadenylation of simian virus 40 late pre-mRNAs.	https://www.ncbi.nlm.nih.gov/pubmed/3037325	y	1987	Sheets MD
745	Polyadenylic acid metabolizing enzyme levels during induction of differentiation in a human leukemia T-cell line with phorbol ester.	https://www.ncbi.nlm.nih.gov/pubmed/3029496	y	1987	Perez S
746	Polyadenylated and nonadenylated messenger RNA and androgen control of sexual behavior and scent marking in male gerbils.	https://www.ncbi.nlm.nih.gov/pubmed/2881874	y	1987	Yahr P
747	The effect of abscisic acid on the differential expression of Î±-amylase isozymes in barley aleurone layers.	https://www.ncbi.nlm.nih.gov/pubmed/24302520	y	1987	Nolan RC
748	RNA synthesis inhibitors on young rat adrenal in primary culture. An ultrastructural study.	https://www.ncbi.nlm.nih.gov/pubmed/3495905	y	1987	MAGALHAES MC
750	Poly-A+ mRNA and defeminization of sexual behavior and gonadotropin secretion in rats.	https://www.ncbi.nlm.nih.gov/pubmed/2885870	y	1987	Ulibarri C
752	Sindbis virus infection increases hexose transport in quiescent cells.	https://www.ncbi.nlm.nih.gov/pubmed/3024395	y	1986	Garry RF
754	Estrogen induction of sexual behavior in female rats and synthesis of polyadenylated messenger RNA in the ventromedial nucleus of the hypothalamus.	https://www.ncbi.nlm.nih.gov/pubmed/2878707	y	1986	Yahr P
755	Treatment of Chinese hamster ovary cells with the transcriptional inhibitor actinomycin D inhibits binding of messenger RNA to ribosomes.	https://www.ncbi.nlm.nih.gov/pubmed/3790527	y	1986	Kostura M
757	Analysis of RNA cleavage at the adenovirus-2 L3 polyadenylation site.	https://www.ncbi.nlm.nih.gov/pubmed/3019671	y	1986	Moore CL
761	Amino acid-dependent inactivation of glucagon-induced System A transport activity in cultured rat hepatocytes.	https://www.ncbi.nlm.nih.gov/pubmed/4065425	y	1985	Handlogten ME
764	The metabolism of 5'-methylthioadenosine and 5-methylthioribose 1-phosphate in Saccharomyces cerevisiae.	https://www.ncbi.nlm.nih.gov/pubmed/3906034	y	1985	Marchitto KS

765	Combinations of interferon-gamma and retinoic acid or 1 alpha, 25-dihydroxycholecalciferol induce differentiation of the human monoblast leukemia cell line U-937.	https://www.ncbi.nlm.nih.gov/pubmed/2411582	y	1985	Gullberg U
767	Dissociation of cells from sea urchin embryos alters the synthesis of actins and other proteins.	https://www.ncbi.nlm.nih.gov/pubmed/3875415	y	1985	Maglott DR
768	Glucocorticoid modulation of collagenase expression in human skin fibroblast cultures. Evidence for pre-translational inhibition.	https://www.ncbi.nlm.nih.gov/pubmed/2988628	y	1985	Bauer EA
769	Induction of myelin components: cyclic AMP increases the synthesis rate of 2',3'-cyclic nucleotide 3'-phosphohydrolase in C6 glioma cells.	https://www.ncbi.nlm.nih.gov/pubmed/2983029	y	1985	McMorris FA
770	Regulation of the 1,25-dihydroxyvitamin D3 receptor by 1,25-dihydroxyvitamin D3 in intact human cancer cells.	https://www.ncbi.nlm.nih.gov/pubmed/2982581	y	1985	Sher E
771	Radiolabeling of DNA with 3' terminal transferase.	https://www.ncbi.nlm.nih.gov/pubmed/21374204	y	1985	Gaastra W
774	Purification and properties of adenosine kinase from rat liver: separation from deoxyadenosine kinase activity.	https://www.ncbi.nlm.nih.gov/pubmed/2986372	y	1985	Drabikowska AK
776	Activation of portal-hepatic osmoreceptors in rats: role of calcium, acetylcholine and cyclic AMP.	https://www.ncbi.nlm.nih.gov/pubmed/6150955	y	1984	Stoppini L
778	On the effect of glucagon on mitochondrial calcium retention in isolated hepatocytes.	https://www.ncbi.nlm.nih.gov/pubmed/6500486	y	1984	Hughes BP
779	3'-Deoxyinosine as an anti-leishmanial agent: the metabolism and cytotoxic effects of 3'-deoxyinosine in Leishmania tropica promastigotes.	https://www.ncbi.nlm.nih.gov/pubmed/6487305	y	1984	Wataya Y
780	Nuclear RNA metabolism in rat hippocampal slices incubated in vitro.	https://www.ncbi.nlm.nih.gov/pubmed/6205720	y	1984	Dokas LA
782	Glutamine synthetase in cultured whole retinas from the embryonic chick. Role of protein and RNA syntheses in 4 degrees C storage enhancement.	https://www.ncbi.nlm.nih.gov/pubmed/6207921	y	1984	PIPERBERG JB

783	Evidence for post-transcriptional regulation by insulin of 3-hydroxy-3-methylglutaryl coenzyme A reductase and sterol synthesis in human mononuclear leucocytes.	https://www.ncbi.nlm.nih.gov/pubmed/6376245	y	1984	Krone W
785	Androgen regulation of poly(A) polymerase in the ventral prostate of rat.	https://www.ncbi.nlm.nih.gov/pubmed/6098056	y	1984	Raju VS
786	Spectrophotometric studies of the interaction of S-adenosylhomocysteinase with adenosine, adenine and cordycepin.	https://www.ncbi.nlm.nih.gov/pubmed/6608376	y	1984	Gomi T
789	Physical change in cytoplasmic messenger ribonucleoproteins in cells treated with inhibitors of mRNA transcription.	https://www.ncbi.nlm.nih.gov/pubmed/6717428	y	1984	DREYFUSS G
790	Decreased isoproterenol-induced "down"-regulation of beta-adrenergic receptors in the myocardium of SHR.	https://www.ncbi.nlm.nih.gov/pubmed/6327523	y	1984	Limas CJ
791	Anti-parasite activity of nucleoside analogues in Leishmania tropica promastigotes.	https://www.ncbi.nlm.nih.gov/pubmed/6522295	y	1984	Wataya Y
792	Short- and long-term studies on chemical carcinogenesis in BALB/Mo mice.	https://www.ncbi.nlm.nih.gov/pubmed/6335923	y	1984	BIANCHI LB
794	Modulation of production of hepatitis B surface antigen by a human hepatoma cell line.	https://www.ncbi.nlm.nih.gov/pubmed/6198456	y	1984	Clementi M
795	Inhibition of protein synthesis in CHO cells by actinomycin D: lesion occurs after 40S initiation complex formation.	https://www.ncbi.nlm.nih.gov/pubmed/6197992	y	1983	Craig N
796	Fast ephemeral DNA damage upon BaP injection.	https://www.ncbi.nlm.nih.gov/pubmed/6320364	y	1983	HOHN-BENTZ J
798	Effects of cordycepin on the synthesis of nuclear and cytoplasmic RNAs in embryonic cells of Xenopus laevis.	https://www.ncbi.nlm.nih.gov/pubmed/6201296	y	1983	Shiokawa K
800	Contribution of maternal mRNA for maintenance of Ca2+-dependent reaggregating activity in dissociated cells of Xenopus laevis embryos.	https://www.ncbi.nlm.nih.gov/pubmed/6199118	y	1983	Shiokawa K

801	Resistance to 9-beta-D-arabinofuranosyladenine in cultured leukemia L 1210 cells.	https://www.ncbi.nlm.nih.gov/pubmed/6603904	y	1983	Cass CE
804	Insulin regulation of fat cell ribosomes, protein synthesis, and lipoprotein lipase.	https://www.ncbi.nlm.nih.gov/pubmed/6410926	y	1983	VYDELINGUM N
805	Effect of cordycepin(3'-deoxyadenosine) on excision repair of 5,6-dihydroxy-dihydrothymine-type products from the DNA of Micrococcus radiodurans.	https://www.ncbi.nlm.nih.gov/pubmed/6603631	y	1983	Patil MS
806	Modulation of GABA receptor binding by Ca2+.	https://www.ncbi.nlm.nih.gov/pubmed/6306170	y	1983	Corda MG
807	The effect of poly(A) segment size on the inhibition of hnRNA biosynthesis by cordycepin in rat brain cells.	https://www.ncbi.nlm.nih.gov/pubmed/6190510	y	1983	KUZNETSOV DA
808	Studies of the mechanism of glucocorticoid-induced pyknosis in isolated rat thymocytes.	https://www.ncbi.nlm.nih.gov/pubmed/6855228	y	1983	Thomas N
810	Endocrine regulation of spermatogenesis in Nereis diversicolor (annelida polychaeta): experimental study of the control of meiotic differentiation.	https://www.ncbi.nlm.nih.gov/pubmed/6189962	y	1983	BERTOUT M
812	Poly A (+) mRNA metabolism in wing imaginal discs during normal development and diapause in Pieris brassicae.	https://www.ncbi.nlm.nih.gov/pubmed/6133562	y	1983	TARROUX P
813	A three-stage chromatographic procedure for cordycepin. Quantitative estimation in rat brain tissue.	https://www.ncbi.nlm.nih.gov/pubmed/6603839	y	1983	Musaev NI
814	Enzymic differentiation in cultured foetal hepatocytes of the rat. Induction of serine dehydratase activity by dexamethasone and dibutyryl cyclic AMP.	https://www.ncbi.nlm.nih.gov/pubmed/6313460	y	1983	Oliver IT
815	Regulation of tyrosine aminotransferase messenger ribonucleic acid in rat liver. effect of cycloheximide on messenger ribonucleic acid turnover.	https://www.ncbi.nlm.nih.gov/pubmed/6130784	y	1982	Ernest MJ
818	Inhibition of protein synthesis blocks the response to 25-hydroxycholesterol by inhibiting degradation of hydroxymethylglutaryl-CoA reductase.	https://www.ncbi.nlm.nih.gov/pubmed/7126620	y	1982	CHEN HW

819	Quantitative estimate of unlabelled cordycepin in acid-soluble pool isolated from rat brain tissue after intraperitoneal injection of the inhibitor.	https://www.ncbi.nlm.nih.gov/pubmed/6982912	y	1982	KUZNETSOV DA
820	Rapid changes in the concentration of phosphoenolpyruvate carboxykinase mRNA in rat liver and kidney. Effects of insulin and cyclic AMP.	https://www.ncbi.nlm.nih.gov/pubmed/6282847	y	1982	Cimbala MA
821	Storage of embryonically transcribed poly(A) RNA and its utilization during metamorphosis of the hydroid Hydractinia echinata.	https://www.ncbi.nlm.nih.gov/pubmed/28305058	y	1982	Eiben R
822	Association of spermine and 4S RNA during axonal transport in regenerating optic nerves of goldfish.	https://www.ncbi.nlm.nih.gov/pubmed/6178462	y	1982	INGOGLIA NA
823	Regulation of ornithine decarboxylase activity by cyclic AMP in guinea pig lymphocytes: transcriptional and post-transcriptional control.	https://www.ncbi.nlm.nih.gov/pubmed/6277383	y	1982	OTANI S
825	Different levels of gene realization in the diurnal control of rat liver enzymes.	https://www.ncbi.nlm.nih.gov/pubmed/6174090	y	1981	HOFFMANN J
826	Nuclear RNA is spliced in the absence of poly(A) addition.	https://www.ncbi.nlm.nih.gov/pubmed/6976837	y	1981	Zeevi M
827	Regulation of rat liver phosphoenolpyruvate carboxykinase (GTP) messenger ribonucleic acid activity by N6, O2'-dibutyryladenosine 3',5'-phosphate.	https://www.ncbi.nlm.nih.gov/pubmed/6271173	y	1981	Beale EG
830	Polyribosome formation and poly(A)-containing RNA in embryos of the sand dollar,Dendraster excentricus.	https://www.ncbi.nlm.nih.gov/pubmed/28305360	y	1981	Spieth J
831	Electron microscope visualization of giant polysomes in sea urchin embryos.	https://www.ncbi.nlm.nih.gov/pubmed/28305354	y	1981	Whitetey AH
832	The control of growth of mouse mastocytoma cells by N6,O2'-dibutyryladenosine cyclic 3',5'-monophosphate.	https://www.ncbi.nlm.nih.gov/pubmed/6163957	y	1981	Knightbridge A
833	Chirally selective synthesis of sugar moiety of nucleosides by chemicoenzymatic approach: L- and D-riboses, showdomycin, and cordycepin.	https://www.ncbi.nlm.nih.gov/pubmed/6796946	y	1981	Ito Y

835	The regulation of cholesterol synthesis in human cells.	https://www.ncbi.nlm.nih.gov/pubmed/6172394		1981	
836	Studies on the control of plasminogen activator production by cultured human embryonic lung cells: requirements for inhibition by corticosteroids.	https://www.ncbi.nlm.nih.gov/pubmed/7193216	y	1980	RIFKIN DB
837	Effects of cordycepin and cell dissociation on the synthesis of H1 histone by sea urchin embryos.	https://www.ncbi.nlm.nih.gov/pubmed/6969119	y	1980	BROOKBANK JW
838	Nucleocytoplasmic transport of RNA. The effect of 3'-deoxyadenosine triphosphate on RNA release from isolated nuclei.	https://www.ncbi.nlm.nih.gov/pubmed/6165357	y	1980	KLETZIEN RF
839	Inhibition of X-ray-induced potentially lethal damage (PLD) repair by cordycepin (3'-deoxyadenosine) and enhancement of its action by 2'-deoxycoformycin in Chinese hamster hai cells in the stationary phase in vitro	https://www.ncbi.nlm.nih.gov/pubmed/6969408	y	1980	NAKATSUGAWA S
840	Evidence that the inhibitory effect of adenosine, but not cordycepin, on the methylation of nuclear RNA is mediated by S-adenosylhomocysteine hydrolase.	https://www.ncbi.nlm.nih.gov/pubmed/6162090	y	1980	GLAZER RI
842	Inhibition of 40S--Met--tRNAfMet ribosomal initiation complex formation by vaccinia virus	https://www.ncbi.nlm.nih.gov/pubmed/6968407	y	1980	PERSON A
843	Effect of 3'-deoxyadenosine (cordycepin) on the early development of the sand dollar, Dendraster excentricus.	https://www.ncbi.nlm.nih.gov/pubmed/6157592	y	1980	SPIETH J
844	Early effect of estrogen on chromatin ultrastructure in endometrial nuclei.	https://www.ncbi.nlm.nih.gov/pubmed/7390003	y	1980	Vic P
845	3'-end labeling of DNA with [alpha-32P]cordycepin-5'-triphosphate.	https://www.ncbi.nlm.nih.gov/pubmed/6248422	y	1980	Tu CD
846	Inhibitors of RNA synthesis and passage of chick embryo fibroblasts through the G1 period.	https://www.ncbi.nlm.nih.gov/pubmed/6160163	y	1980	CHADWICK D
847	Inhibitory effect of cyclic adenosine 2',3'-monophosphate on leucine incorporation by L5178Y cells.	https://www.ncbi.nlm.nih.gov/pubmed/6159364	y	1980	FUHR JE

848	Cultured animal cells exposed to amino acid analogues or puromycin rapidly synthesize several polypeptides.	https://www.ncbi.nlm.nih.gov/pubmed/6901532	y	1980	HIGHTOWER LE
849	Requirement of RNA and protein synthesis and inhibition by ethylenediaminetetraacetic acid of retinoic acid-induced proteoglycan release in a transplantable rat chondrosarcoma.	https://www.ncbi.nlm.nih.gov/pubmed/6766513	y	1980	Kistler A
851	Enhanced killing of Chinese hamster cells by alpha-irradiation from plutonium-238 in the presence of cordycepin.	https://www.ncbi.nlm.nih.gov/pubmed/6966268	y	1980	ROBERTSON JB
852	Germ tube induction in Candida albicans.	https://www.ncbi.nlm.nih.gov/pubmed/6996798	y	1980	SHEPHERD MG
853	Shut-off of host protein synthesis in vaccinia-virus-infected cells exposed to cordycepin. A study in vitro.	https://www.ncbi.nlm.nih.gov/pubmed/6965641	y	1980	PERSON A
854	A comparative analysis of the effect of actinomycin-D, alpha-amanitin an cordycepin on RNA synthesis and N-acetyltransferase induction by isoproterenol in cultured rat pineals.	https://www.ncbi.nlm.nih.gov/pubmed/6161857	y	1980	MILLER LP
855	Synchronization of MEL cell commitment with cordycepin.	https://www.ncbi.nlm.nih.gov/pubmed/316363	y	1979	Levenson R
856	The effects of actinomycin D and cordycepin on neurite formation and acetylcholinesterase activity in mouse neuroblastoma cells.	https://www.ncbi.nlm.nih.gov/pubmed/315325	y	1979	BEAR MP
857	Regulation of sterol synthesis in human lymphocytes: evidence for post-transcriptional control by low density lipoprotein.	https://www.ncbi.nlm.nih.gov/pubmed/226155	y	1979	KRONE W
860	Stimulation of chick gut alkaline phosphatase activity by actinomycin D and 1,25-dihydroxyvitamin D3: evidence for independent mechanisms.	https://www.ncbi.nlm.nih.gov/pubmed/313969	y	1979	Bikle DD
861	Cellular localization of Saint Louis encephalitis virus replication.	https://www.ncbi.nlm.nih.gov/pubmed/40415	y	1979	Brawner IA
862	Differential effects of cordycepin triphosphate and 9 beta-D-arabinofuranosyladenine triphosphate on tRNA and 5 S RNA synthesis in isolated nuclei.	https://www.ncbi.nlm.nih.gov/pubmed/497205	y	1979	LEONARD TB

863	Studies of two temperature-sensitive mutants of Mengo virus.	https://www.ncbi.nlm.nih.gov/pubmed/224989	y	1979	LEE PK
864	Evidence for the involvement of lutropin-independent RNA synthesis in Leydig cell steroidogenesis.	https://www.ncbi.nlm.nih.gov/pubmed/223901	y	1979	COOKE BA
865	Inhibition of ribonucleic acid efflux from isolated SV40-3T3 cell nuclei by 3'-deoxyadenosine (cordycepin).	https://www.ncbi.nlm.nih.gov/pubmed/226073	y	1979	AGUTTER PS
866	Evidence that xylosyladenine affects methylation by inhibition of S-adenosyl-L-methionine synthesis.	https://www.ncbi.nlm.nih.gov/pubmed/312136	y	1979	GLAZER RI
867	Metabolism of polyadenylated mRNA in growing human lymphocytes.	https://www.ncbi.nlm.nih.gov/pubmed/312113	y	1979	BERGER SL
869	Effects of thermic shock on HEp-2 cells. II. Inhibition of induction of perichromatin granules by cordycepin and actinomycin D.	https://www.ncbi.nlm.nih.gov/pubmed/311856	y	1979	CERVERA J
872	Effects of cordycepin on microtubules of cultured mammalian cells.	https://www.ncbi.nlm.nih.gov/pubmed/310391	y	1979	DEITCH AD
873	Influence of L-tryptophan on the activity of tryptophan oxygenase in rat liver slices.	https://www.ncbi.nlm.nih.gov/pubmed/40582	y	1979	Kröger H
874	Effect of dibutyryl cyclic AMP on leucine incorporation in L5178Y cells. II. Effect of actinomycin and heat shock.	https://www.ncbi.nlm.nih.gov/pubmed/83323	y	1978	FUHR JE
875	Inhibition of the phosphorylation of nonhistone chromosomal proteins by cordycepin and xylosyladenine in L1210 cells in vitro.	https://www.ncbi.nlm.nih.gov/pubmed/310509	y	1978	Legraverend M
876	Enhancement of radiation killing of cultured mammalian cells by cordycepin.	https://www.ncbi.nlm.nih.gov/pubmed/224001	y	1978	ROBERTSON JB
877	Yeast temperature-sensitive mutants specifically impaired in processing of poly(A)-containing RNAs.	https://www.ncbi.nlm.nih.gov/pubmed/366372	y	1978	Bloch JC

878	The effects of 2'-deoxycoformycin on the action of cordycepin on nuclear RNA synthesis in regenerating liver.	https://www.ncbi.nlm.nih.gov/pubmed/310180	y	1978	Glazer RI
879	Potentiation by 2'-deoxycoformycin of the inhibitory effect by 3'-deoxyadenosine (cordycepin) on nuclear RNA synthesis in L1210 cells in vitro.	https://www.ncbi.nlm.nih.gov/pubmed/307428	y	1978	Glazer RI
880	Differential behavior of two dexamethasone induced mRNA activities in HTC cells in response to cordycepin and to withdrawal of hormone.	https://www.ncbi.nlm.nih.gov/pubmed/29627	y	1978	Land H
881	Cellular and molecular toxicology of lead. II. Effect of lead on delta-aminolevulinic acid synthetase of cultured cells.	https://www.ncbi.nlm.nih.gov/pubmed/308103	y	1978	Kusell M
882	Acceleration of CHO cells into mitosis and reduction of x-ray-induced G2 delay by cordycepin.	https://www.ncbi.nlm.nih.gov/pubmed/308009	y	1978	TOMASOVIC SP
883	Studies on the mechanisms of vaccina virus cytopathic effects. I. Inhibition of protein synthesis in infected cells is associated with virus-induced RNA synthesis.	https://www.ncbi.nlm.nih.gov/pubmed/307049	y	1987	BABLANIAN R
884	3' deoxycytidine, like hydroxyurea, inhibits DNA synthesis without preventing the initiation of the cell cycle.	https://www.ncbi.nlm.nih.gov/pubmed/667965	y	1978	Brooks RF
885	The effect of intraocular injection of cordycepin on retinal RNA synthesis and on RNA axonally transported during regeneration of the optic nerves of goldfish.	https://www.ncbi.nlm.nih.gov/pubmed/77895	y	1978	INGOGLIA NA
886	The effect of inhibitors of cellular RNA synthesis on stimulation of mouse encephalomyocarditis virus reproduction by poliovirus in HeLa and MIO cells.	https://www.ncbi.nlm.nih.gov/pubmed/27964	y	1978	Shirman GA
887	Poly(A) polymerase activity during cell cycle and erythropoietic differentiation in erythroleukemic mouse spleen cells.	https://www.ncbi.nlm.nih.gov/pubmed/207327	y	1978	Adolf GR
888	Cordycepin. An inhibitor of newly synthesized globin messenger RNA.	https://www.ncbi.nlm.nih.gov/pubmed/305436	y	1978	Beach LR
889	Superinduction of ornithine decarboxylase by actinomycin D and cordycepin.	https://www.ncbi.nlm.nih.gov/pubmed/208537	y	1978	Costa M

890	Inhibition of the phosphorylation of non-histone chromosomal proteins of rat liver by cordycepin and cordycepin triphosphate.	https://www.ncbi.nlm.nih.gov/pubmed/305821	y	1978	Legraverend M
891	Cordycepin and xylosyladenine: inhibitors of methylation of nuclear RNA.	https://www.ncbi.nlm.nih.gov/pubmed/307384	y	1978	GLAZER RI
892	Dibutyryl cyclic AMP increases the amount of functional messenger RNA coding for tyrosine aminotransferase in rat liver.	https://www.ncbi.nlm.nih.gov/pubmed/24052	y	1978	Noguchi T
893	Evidence for highly stable nuclear poly(A) in cultured mammalian cells.	https://www.ncbi.nlm.nih.gov/pubmed/304742	y	1978	HENDRICKSON S
894	Synthesis of RNA in the pineal gland during N-acetyltransferase induction. The effects of actinomycin D, alpha-amanitin and cordycepin.	https://www.ncbi.nlm.nih.gov/pubmed/305246	y	1978	MORRISSEY JJ
895	Role of RNA in the action of aldosterone on Na+ transport.	https://www.ncbi.nlm.nih.gov/pubmed/366153	y	1978	Rossier BC
897	Glucocorticoid regulation of rat thymus RNA polymerase activity: the role of RNA and protein synthesis.	https://www.ncbi.nlm.nih.gov/pubmed/304818	y	1978	BORTHWICK NM
898	Inhibition of host protein synthesis in vaccinia virus-infected cells in the presence of cordycepin (3'-deoxyadenosine).	https://www.ncbi.nlm.nih.gov/pubmed/304489	y	1978	PERSON A
899	Effect of cordycepin on CRF stimulation and steroid inhibition of ACTH secretion by rat pituitary cells.	https://www.ncbi.nlm.nih.gov/pubmed/203501	y	1978	BRATTIN WJ
900	Effect of cordycepin triphosphate on in vitro RNA synthesis by picornavirus polymerase complexes.	https://www.ncbi.nlm.nih.gov/pubmed/203501	y	1978	PANICALI DL
901	Inhibition of RNA and protein syntheses makes non-differentiating mouse myeloid leukemia cells sensitive to a factor(s) stimulating differentiation.	https://www.ncbi.nlm.nih.gov/pubmed/271144	y	1977	OKABE J
903	Segregation during cleavage of a factor determining endodermal alkaline phosphatase development in ascidian embryos.	https://www.ncbi.nlm.nih.gov/pubmed/411883	y	1977	WHITTAKER JR

904	Effects of cordycepin on macromolecular synthesis and development in the preimplantation mouse embryo.	https://www.ncbi.nlm.nih.gov/pubmed/303178	y	1977	LEVEY IL
905	Effect of cordycepin on nucleic acid metabolism in L5178Y cells and on nucleic acid-synthesizing enzyme systems.	https://www.ncbi.nlm.nih.gov/pubmed/332340	y	1977	Muller WE
907	Relative responses of an X-ray-resistant hybrid cell-line and its parent line to X-irradiation, ultraviolet light, actinomycin D and cordycepin.	https://www.ncbi.nlm.nih.gov/pubmed/301863	y	1977	ROBERTSON JB
908	Specific inhibition of chromatin-associated poly(A) synthesis in vitro by cordycepin 5'-triphosphate.	https://www.ncbi.nlm.nih.gov/pubmed/16073440	y	1977	Rose KM
909	Transport enhancement and reversal: glucose and 3-O-methyl glucose.	https://www.ncbi.nlm.nih.gov/pubmed/301142	y	1977	MUSLINER TA
910	Selective inhibition of initial polyadenylation in isolated nuclei by low levels of cordycepin 5"-triphosphate.	https://www.ncbi.nlm.nih.gov/pubmed/300631	y	1977	Rose KM
911	Increase in level of functional messenger RNA coding for phosphoenolpyruvate carboxykinase (GTP) during induction by cyclic adenosine 3':5'-monophosphate.	https://www.ncbi.nlm.nih.gov/pubmed/188822	y	1977	IYNEDJIAN PB
912	The effect of tryptophan on nucleocytoplasmic translocation of RNA in rat liver.	https://www.ncbi.nlm.nih.gov/pubmed/299816	y	1977	MURTY CN
914	The effect of cordycepin on the multiplication of Semliki Forest virus and on polyadenylation of viral RNA.	https://www.ncbi.nlm.nih.gov/pubmed/302113	y	1977	WITTEK R
916	Survival of synchronized V79 cells treated with X-rays and cordycepin.	https://www.ncbi.nlm.nih.gov/pubmed/300368	y	1977	ROBERTSON JB
917	Reversible inhibition of interferon-induced antiviral state by deoxyadenosine.	https://www.ncbi.nlm.nih.gov/pubmed/13641	y	1976	Matsuno T
918	Interaction between glucocorticoids and cyclic AMP in the regulation of phosphoenolpyruvate carboxykinase (GTP) in the isolated perfused rat liver. Effects of cordycepin and cycloheximide.	https://www.ncbi.nlm.nih.gov/pubmed/188461	y	1976	KRONE W

919	Poly(adenylic acid) synthesis in isolated rat liver mitochondria.	https://www.ncbi.nlm.nih.gov/pubmed/990263	y	1976	Rose KM
920	Nucleosides. 1. 9-(3'-Alkyl-3'-deoxy-beta-D-ribofuranosyl)adenines as lipophilic analogues of cordycepin. Synthesis and preliminary biological studies.	https://www.ncbi.nlm.nih.gov/pubmed/1087343	y	1976	Rosowsky A
921	Regulation of glucose-6-phosphate dehydrogenase activity in uterine tissue in organ culture.	https://www.ncbi.nlm.nih.gov/pubmed/1086769	y	1976	KERAN EE
922	Effect of cold exposure on phosphoenolpyruvate carboxykinase (GTP) activity and cyclic amp concentration in livers of starved rats. Role of glucorticoids.	https://www.ncbi.nlm.nih.gov/pubmed/186103	y	1976	KRONE W
923	Influenza viral mRNA contains internal N6-methyladenosine and 5'-terminal 7-methylguanosine in cap structures.	https://www.ncbi.nlm.nih.gov/pubmed/1086370	y	1976	KRUG RM
924	Polyadenylate sequences of human rhinovirus and poliovirus RNA and cordycepin sensitivity of virus replication.	https://www.ncbi.nlm.nih.gov/pubmed/185411	y	1976	NAIR CN
926	Nuclear poly(A) polymerase from rat liver and a hepatoma. Comparison of properties, molecular weights and amino acid compositions.	https://www.ncbi.nlm.nih.gov/pubmed/183950	y	1976	Rose KM
927	Cyproterone-mediated stimulation of delta-aminolevulinic acid synthetase in chick embryo liver cells.	https://www.ncbi.nlm.nih.gov/pubmed/1084831	y	1976	GIDARI AS
928	The effect of cordycepin on the appearance of [3H]RNA in the goldfish optic tectum following intraocular injection of [3H]uridine.	https://www.ncbi.nlm.nih.gov/pubmed/60467	y	1976	INGOGLIA NA
929	The synthesis of 3'-dATP and its use as an inhibitor of ATP-dependent DNA synthesis in toluene-treated Escherichia coli.	https://www.ncbi.nlm.nih.gov/pubmed/779831	y	1976	Gumport RI
930	Enhancement of the biological activity of cordycepin (3'-deoxyadenosine) by the adenosine deaminase inhibitor 2'-deoxycoformycin.	https://www.ncbi.nlm.nih.gov/pubmed/1084747	y	1976	Johns DG
931	Studies on the mode of action of ecdysterone in adult female Aedes aegypti.	https://www.ncbi.nlm.nih.gov/pubmed/1084837	y	1976	FONG WF

932	Differentiation of thymocytes: evidence that induction of the surface phenotype requires transcription and translation.	https://www.ncbi.nlm.nih.gov/pubmed/1083871	y	1976	Storrie B
934	Stimulatory actions of thyrotropin and dibutyryl cyclic AMP on transcription and translation in the regulation of thyroidal protein synthesis.	https://www.ncbi.nlm.nih.gov/pubmed/177054	y	1976	SHERWIN JR
935	Utilization of uridine for RNA synthesis in the insect cell line CP-1268 derived from the codling moth, Laspeyresia pomonella.	https://www.ncbi.nlm.nih.gov/pubmed/1083370	y	1976	GALLAGHER BM
936	Stimulation of melanotic expression in a melanoma cell line by theophylline.	https://www.ncbi.nlm.nih.gov/pubmed/815264	y	1976	STEINBERG ML
937	Effects of inhibitors of protein and RNA synthesis on aldosterone-stimulated changes in phospholipid fatty acid metabolism in the toad urinary bladder.	https://www.ncbi.nlm.nih.gov/pubmed/1082772	y	1976	LIEN EL
938	The sensitivity of RNA polymerases I and II from Novikoff hepatoma (N1S1) cells to 3'-deoxyadenosine 5'-triphosphate.	https://www.ncbi.nlm.nih.gov/pubmed/176630	y	1976	Desrosiers RC
939	The action of cordycepin on nascent nuclear RNA and poly(A) synthesis in regenerating liver.	https://www.ncbi.nlm.nih.gov/pubmed/1082347	y	1976	GLAZER RI
940	Cordycepin and early effects of estradiol on the immature rat uterus.	https://www.ncbi.nlm.nih.gov/pubmed/1088412	y	1976	BORGNA J
941	Effect of cordycepin on the replication of western equine encephalitis virus.	https://www.ncbi.nlm.nih.gov/pubmed/1087872	y	1976	HASJIIMOTO K
942	Drug effects on the fine structure of Trypanosoma rhodesiense: puromycin and its aminonucleoside, Cordycepin and Nucleocidin.	https://www.ncbi.nlm.nih.gov/pubmed/1085509	y	1976	WILLIAMSON J
943	Cordycepin and early effects of estradiol on the immature rat uterus.	https://www.ncbi.nlm.nih.gov/pubmed/1084764	y	1976	BORGNA JL
945	Effect of cordycepin triphosphate on the nuclear DNA-dependent RNA polymerases and poly(A) polymerase from the yeast, Saccharomyces cerevisiae.	https://www.ncbi.nlm.nih.gov/pubmed/766704	y	1976	HOROWITZ B

946	Dependence of gonadotropin-induced steroidogenesis upon RNA and protein synthesis in the interstitial cells of the rat testis.	https://www.ncbi.nlm.nih.gov/pubmed/172138	y	1975	MENDELSON C
947	A kinetic and structural characterization of adenosine-5'-triphosphate: ribonucleic acid adenylyltransferase from Pseudomonas putida.	https://www.ncbi.nlm.nih.gov/pubmed/1191706	y	1975	BLAKESLEY RW
948	Effect of cordycepin (3'-deoxyadenosine) on virus-specific RNA species synthesized in Newcastle disease virus-infected cells.	https://www.ncbi.nlm.nih.gov/pubmed/1081601	y	1975	WEISS SR
949	Cordycepin inhibition of 3-methylcholanthrene-induced transformation in vitro.	https://www.ncbi.nlm.nih.gov/pubmed/54927	y	1975	PRICE PJ
950	Three distinct forms of nuclear poly(A) polymerase.	https://www.ncbi.nlm.nih.gov/pubmed/1081945	y	1975	NIESSING J
951	Recovery of a DNA-protein complex in cultured mammalian cells from damage caused by 4-nitroquinoline 1-oxide.	https://www.ncbi.nlm.nih.gov/pubmed/810243	y	1975	Ide T
952	The effects of metabolic inhibitors on the synthesis of inducible tyrosine aminotransferase in cultured hepatoma cells.	https://www.ncbi.nlm.nih.gov/pubmed/240862	y	1975	DETHLEFSEN LA
954	Effects of cyclic adenosine monophosphate, dexamethasone and insulin on phosphoenolpyruvate carboxykinase synthesis in Reuber H-35 hepatoma cells.	https://www.ncbi.nlm.nih.gov/pubmed/166654	y	1975	Gunn JM
955	Two approaches that increase the activity of analogs of adenine nucleosides in animal cells.	https://www.ncbi.nlm.nih.gov/pubmed/1079475	y	1975	Plunkett W
956	The relation of endogenous adenosine cyclic 3':5'-monophosphate to the antagonistic effects of adenosine and colchicine on cell shape.	https://www.ncbi.nlm.nih.gov/pubmed/167038	y	1975	YIN HH
957	Induction of specific changes in the surface membrane of myeloid leukemic cells by steroid hormones.	https://www.ncbi.nlm.nih.gov/pubmed/1079793	y	1975	LOTEM J
958	Differential inhibition of the in vivo synthesis of adenovirus type 5-specific ribonucleic acids by cordycepin.	https://www.ncbi.nlm.nih.gov/pubmed/1079985	y	1975	VAN OORTMERSSEN EA

959	Effects of cordycepin on RNA sythesis in Physarum polycephalum.	https://www.ncbi.nlm.nih.gov/pubmed/1079713	y	1975	FOUQUET H
960	Mouse embryo development in vitro: effects of inhibitors of RNA and protein synthesis on blastocyst and post-blastocyst embryos.	https://www.ncbi.nlm.nih.gov/pubmed/1079529	y	1975	ROWINSKI J
961	Effects of cordycepin and cordycepintriphosphate on polyadenylic and ribonucleic acid-synthesising enzymes from eukaryotes.	https://www.ncbi.nlm.nih.gov/pubmed/1079328	y	1975	Maale G
962	Superinduction" of tyrosine aminotransferase by actinomycin D: a reevaluation.	https://www.ncbi.nlm.nih.gov/pubmed/236835	y	1975	Steinberg RA
964	Effect of cordycepin on the replication of type-c RNA tumor viruses.	https://www.ncbi.nlm.nih.gov/pubmed/166934	y	1975	RICHARDSON LS
966	Poly (A)-containing polyribosomal RNA in sea urchin embryos: changes in proportion during development.	https://www.ncbi.nlm.nih.gov/pubmed/1115788	y	1975	FROMSON D
968	Changes in RNA in relation to growth of the fibroblast. III. Posttranscriptional regulation of mRNA formation in resting and growing cells.	https://www.ncbi.nlm.nih.gov/pubmed/1078787	y	1975	Johnson LF
969	Early stimulation of human chorionic gonadotropin secretion by dibutyryl cyclic AMP and theophylline in human malignant trophoblast cells in vitro: inhibition by actinomycin D, alpha-amanitin, and cordycepin	https://www.ncbi.nlm.nih.gov/pubmed/174977	y	1975	Pattilo RA
970	Comparison of drug effects on RNA tumor viruses and on transformed cells.	https://www.ncbi.nlm.nih.gov/pubmed/51639	y	1975	Ting RC
971	Early events in lymphocyte transformation of phytohemagglutinin. III. Inhibition of RNA synthesis and transformation by cordycepin.	https://www.ncbi.nlm.nih.gov/pubmed/4549554	y	1974	POGO BG
972	Elongation of the polyadenylate segment of messenger RNA in the cytoplasm of mammalian cells.	https://www.ncbi.nlm.nih.gov/pubmed/4547843	y	1974	DIEZ J
974	Polyadenylation of vesicular stomatitis virus mRNA.	https://www.ncbi.nlm.nih.gov/pubmed/4363251	y	1974	EHRENFELD E

975	Metabolic differences between normal and neoplastic cells: effects of aminonucleoside on cytoplasmic messenger RNA.	https://pubmed.ncbi.nlm.nih.gov/4361100/	y	1974	CHOLON JJ
976	Modified messenger ribonucleic acid release from isolated hepatic nuclei after inhibition of polyadenylate formation.	https://www.ncbi.nlm.nih.gov/pubmed/4549426	y	1974	SCHUMM DE
977	Post-transcriptional addition of polyadenylic acid to mitochondrial RNA by a cordycepin-insensitive process.	https://www.ncbi.nlm.nih.gov/pubmed/4132416	y	1974	HIRSCH M
978	Effects of cordycepin, hydroxyurea and cycloheximide on histone mRNA synthesis in synchronized HeLa cells.	https://www.ncbi.nlm.nih.gov/pubmed/4547198	y	1974	BREINDL M
980	Preliminary observations pertaining to polyadenylation of rhinovirus RNA.	https://www.ncbi.nlm.nih.gov/pubmed/4359303	y	1974	NAIR CN
981	An early cordycepin-sensitive event in the action of glucocorticoid hormones on rat thymus cells in vitro: evidence that synthesis of new mRNA initiates the earliest metabolic effects of steroid hormones	https://www.ncbi.nlm.nih.gov/pubmed/4549303	y	1974	Young DA
982	Inhibition of Herpes simplex virus replication by cordycepin.	https://www.ncbi.nlm.nih.gov/pubmed/4360291	y	1973	Becker Y
983	The effect of cordycepin on cell transformation by RNA tumor viruses.	https://www.ncbi.nlm.nih.gov/pubmed/4355116	y	1973	LOVINGER GG
985	Effect of cordycepin (3'-desoxyadenosine) on polytene chromosomes of Chironomus pallidivittatus salivary glands.	https://www.ncbi.nlm.nih.gov/pubmed/4125901	y	1973	Diez JL
986	Cordycepin inhibits induction of puffs by ions in Chironomus salivary gland chromosomes.	https://www.ncbi.nlm.nih.gov/pubmed/4541611	y	1973	GOPALAN HN
987	Estimation of the half-life of a secretory protein message.	https://www.ncbi.nlm.nih.gov/pubmed/4540973	y	1973	GRAYSON S
988	Multiplication of influenza virus in the presence of cordycepin, an inhibitor of cellular RNA synthesis.	https://www.ncbi.nlm.nih.gov/pubmed/4541329	y	1973	MAHY BW

989	The effect of cordycepin on the synthesis of nuclear and cytoplasmic D-RNA in mouse liver cells and Ehrlich's carcinoma cells.	https://www.ncbi.nlm.nih.gov/pubmed/4543941	y	1973	Podobed OV
990	The effect of cordycepin on tsetse-borne Trypanosoma vivax infections.	https://www.ncbi.nlm.nih.gov/pubmed/4147878	y	1973	Ayedun BA
991	Cordycepin and alpha-amanitin: inhibitors of transcription as probes of aldosterone action.	https://www.ncbi.nlm.nih.gov/pubmed/4201425	y	1972	Chu LL
993	Cordycepin inhibits induction of murine leukovirus production by 5-iodo-2'-deoxyuridine.	https://www.ncbi.nlm.nih.gov/pubmed/4118874	y	1972	WU AM
994	Effects of cordycepin on morphology and RNA synthesis of amphibian lampbrush chromosomes.	https://www.ncbi.nlm.nih.gov/pubmed/4539058	y	1972	FIUME L
995	Effect of cordycepin on induction of tyrosine aminotransferase employing hepatoma cells in tissue culture.	https://www.ncbi.nlm.nih.gov/pubmed/4403717	y	1972	BUTCHER FR
996	Effect of cordycepin on ribosome formation and enzyme induction in rat liver.	https://www.ncbi.nlm.nih.gov/pubmed/4403711	y	1972	RIZZO AJ
997	Addition of polyadenylate sequences to virus-specific RNA during adenovirus replication.	https://www.ncbi.nlm.nih.gov/pubmed/5315962	y	1971	PHILIPSON L
998	Mitochondrial RNA turnover in the presence of cordycepin.	https://www.ncbi.nlm.nih.gov/pubmed/5315931	y	1971	ZYLBER EA
999	The effect of cordycepin on nuclear RNA synthesis in nerve and glial cells.	https://www.ncbi.nlm.nih.gov/pubmed/4997804	y	1971	GRAHAN B
1000	Effects of 3'deoxyadenosine (cordycepin) and 2'deoxyadenosine on nucleoside transport, macromolecular synthesis, and replication of cultured Novikoff hepatoma cells.	https://www.ncbi.nlm.nih.gov/pubmed/4330131	y	1971	PLAGERIANN PG
1001	Messenger and heterogeneous nuclear RNA in HeLa cells: differential inhibition by cordycepin.	https://www.ncbi.nlm.nih.gov/pubmed/5275385	y	1970	Penman S

1004	The selective interruption of nucleolar RNA synthesis in HeLa cells by cordycepin.	https://www.ncbi.nlm.nih.gov/pubmed/5783871	y	1969	SIEV M
1006	9-(2- and 3-deoxy-beta-d-threo-pentofuranosyl)adenine. The epimers of 2'-deoxyadenosine and 3'-deoxyadenosine(cordycepin).	https://www.ncbi.nlm.nih.gov/pubmed/5917473	y	1966	MARTINEZ AP
1008	THE EFFECTS OF 3'-DEOXYADENOSINE ON THE SYNTHESIS OF RIBONUCLEIC ACID.	https://www.ncbi.nlm.nih.gov/pubmed/14275139	y	1965	SHIGEURA HT
1010	THE INHIBITION OF PHOSPHORIBOSYL-PYROPHOSPHATE AMIDOTRANSFERASE ACTIVITY BY CORDYCEPIN MONOPHOSPHATE.	https://www.ncbi.nlm.nih.gov/pubmed/14209329	y	1964	ROTTMAN F
1011	EFFECT OF 3'-DEOXYATP (CORDYCEPIN TRIPHOSPHATE) AND 2'-DEOXYATP ON THE DNA-DEPENDENT RNA NEUCLEOTIDYLTRANSFERASE FROM EHRLICH ASCITES TUMOR CELLS	https://www.ncbi.nlm.nih.gov/pubmed/14211644	y	1964	KLENOW H
1012	THE INHIBITION OF PURINE BIOSYNTHESIS DE NOVO IN BACILLUS SUBTILIS BY CORDYCEPIN.	https://www.ncbi.nlm.nih.gov/pubmed/14156735	y	1964	ROTTMAN F
1013	STUDIES ON THE INHIBITION OF BACILLUS SUBTILIS GROWTH BY CORDYCEPIN.	https://www.ncbi.nlm.nih.gov/pubmed/14156734	y	1964	ROTTMAN F
1014	THE INHIBITION OF 5-PHOSPHORIBOSYL-1-PYROPHOSPHATE FORMATION BY CORDYCEPIN TRIPHOSPHATE IN EXTRACTS OF EHRLICH ASCITES TUMOR CELLS.	https://www.ncbi.nlm.nih.gov/pubmed/14153854	y	1964	HANSEN KO
1015	EFFECT OF CORDYCEPIN TRIPHOSPHATE ON THE INCORPORATION OF (8-14C)ADENINE AND (32P)	https://www.ncbi.nlm.nih.gov/pubmed/14153853	y	1964	KLENOW H
1016	Identification of cordycepin, a metabolite of Cordyceps militaris, as 3'-deoxyadenosine.	https://www.ncbi.nlm.nih.gov/pubmed/5836541	y	1964	Kaczka EA
1017	INHIBITION OF RIBONUCLEIC ACID AND DEOXYRIBONUCLEIC ACID SYNTHESIS IN EHRLICH ASCITES CELLS BY CORDYCEPIN-N1-OXIDE.	https://www.ncbi.nlm.nih.gov/pubmed/14097399	y	1963	Sune Frederiksen
1018	INHIBITION BY CORDYCEPIN AND 2-DEOXYGLUCOSE OF THE INCORPORATION OF (32P)ORTHOPHOSPHATE INTO THE NUCLEIC ACIDS OF EHRLICH ASCITES-TUMOR CELLS IN VITRO.	https://www.ncbi.nlm.nih.gov/pubmed/14097398	y	1963	KLENOW H

1019	FORMATION OF THE MONO-, DI- AND TRIPHOSPHATE OF CORDYCEPIN IN EHRLICH ASCITES-TUMOR CELLS IN VITRO.	https://www.ncbi.nlm.nih.gov/pubmed/14097397	y	1963	KLENOW H
1020	STUDIES ON THE SYNTHESIS AND STRUCTURE OF CORDYCEPIN MONOPHOSPHATE.	https://www.ncbi.nlm.nih.gov/pubmed/14097374	y	1963	ROTTMAN F
1022	Effect of cordycepin on the incorporation of P32-orthophosphate into the nucleic acids of ascites tumor cells in vitro.	https://www.ncbi.nlm.nih.gov/pubmed/13756769	y	1961	KLENOW H
1023	Improvement on lipid metobolic disorder by 3'-deoxyadenosine in high-fat-diet-induced fatty mice	https://www.ncbi.nlm.nih.gov/pubmed/21061461	y	2010	Niu J
1024	Ribonucleoside triphosphates as substrate of human immunodeficiency virus type 1 transcriptase in human macrophages	https://www.ncbi.nlm.nih.gov/pubmed/20924117	y	2010	Kennedy EM
1025	Cordyceps fungi: natural products, pharmacological functions and developmenta products	https://www.ncbi.nlm.nih.gov/pubmed/19222900	y	2009	Zhou X
1026	A sensitive, single-tube assay to measure the enzymatic activities of influenza RNA polymerase and other poly(A) polymerases: application to kinetic and inhibitor analysis.	https://www.ncbi.nlm.nih.gov/pubmed/11433013	y	2001	Hooker L
1027	Structure of yeast poly(A) polymerase alone and in complex with 3'-dATP	https://www.ncbi.nlm.nih.gov/pubmed/10958780	y	2000	Bard J
1028	Role of histidine residues in the adenosine A2a receptor ligand binding site	https://www.ncbi.nlm.nih.gov/pubmed/7931300	y	1994	Askalan R
1029	Different relationships between cellular adenosine or 3'-deoxyadenosine phosphorylation and cellular adenine ribonucleotide catabolism may be obtained	https://www.ncbi.nlm.nih.gov/pubmed/8419409	y	1993	Overgaard-Hansen K
1031	Poly(A) tail shortening is the translation-dependent step in c-myc mRNA degradation	https://www.ncbi.nlm.nih.gov/pubmed/1701014	y	1990	Laird-Offringa IA
1033	Intracellular inhibition of chromatin binding and transformation of androgen receptor by 3'-deoxyadenosine	https://www.ncbi.nlm.nih.gov/pubmed/3263372	y	1988	Hiipakka RA

1035	Mitochondrial DNA replication proceeds via a 'bootlace' mechanism involving the incorporation of processed transcripts	https://www.ncbi.nlm.nih.gov/pubmed/23595151	y	2013	Reyes A
1036	Photo-crosslinked HAMA hydrogel with cordycepin encapsulated chitosan microspheres for osteoarthritis treatment.	https://pubmed.ncbi.nlm.nih.gov/27926509/	y	2017	Chen Xia
1038	Apoptotic effect of cordycepin combined with cisplatin and/or paclitaxel on MA-10 mouse Leydig tumor cells.	https://www.ncbi.nlm.nih.gov/pubmed/26366090	Y	2015	Fu Chi Kang
1039	Distinct terminal and cell body mechanisms in the nociceptor mediate hyperalgesic priming.	https://www.ncbi.nlm.nih.gov/pubmed/25878283	Y	2015	Luiz F Ferrari
1044	Effects of cordycepin on Y-maze learning task in mice.	https://www.ncbi.nlm.nih.gov/pubmed/23819912	Y	2013	Zhao Lin Cai
1045	Peripheral administration of translation inhibitors reverses increased hyperalgesia in a model of chronic pain in the rat.	https://www.ncbi.nlm.nih.gov/pubmed/23664545	Y	2013	Luiz F Ferrari
1046	Protective roles of Cordyceps on lung fibrosis in cellular and rat models.	https://www.ncbi.nlm.nih.gov/pubmed/22796203	Y	2012	Mengli Chen
1047	Antitumour activity of cordycepin in mice.	https://www.ncbi.nlm.nih.gov/pubmed/15649290	Y	2004	Noriko Yoshikawa
1048	Inhibition of the nerve growth factor-induced outgrowth of neurites by trichostatin A requires protein synthesis de novo in PC12D cells.	https://www.ncbi.nlm.nih.gov/pubmed/9117395	Y	1996	Mamoru Sano
1049	Skeletal muscle-derived trophic factors prevent motoneurons from entering an active cell death program in vitro.	https://www.ncbi.nlm.nih.gov/pubmed/8182435	Y	1994	Joan X Comella
1050	NIH3T3 cells expressing the deleted in colorectal cancer tumor suppressor gene product stimulate neurite outgrowth in rat PC12 pheochromocytoma cells.	https://pubmed.ncbi.nlm.nih.gov/8132705/	Y	1994	William E Pierceall
1051	A molecular circuit composed of CPEB-1 and c-Jun controls growth hormone-mediated synaptic plasticity in the mouse hippocampus.	https://pubmed.ncbi.nlm.nih.gov/18716208/	y	2008	Ruth N Zearfoss

1052	PAP inhibitor with in vivo efficacy identified by Candida albicans genetic profiling of natural products.	https://pubmed.ncbi.nlm.nih.gov/18420143/	y	2008	Bo Jiang
1053	RNA-directed agent, cordycepin, induces cell death in multiple myeloma cells.	https://pubmed.ncbi.nlm.nih.gov/18205859/	y	2008	Lisa S Chen
1054	The role of cordycepin in cancer treatment via induction or inhibition of apoptosis: implication of polyadenylation in a cell type specific manner.	https://www.ncbi.nlm.nih.gov/pubmed/17487491	y	2008	Hellinida Thomadaki
1055	Comparison of protective effects between cultured Cordyceps militaris and natural Cordyceps sinensis against oxidative damage.	https://www.ncbi.nlm.nih.gov/pubmed/16608242	Y	2006	Hui Mei Yu
1056	Inhibitory effects of ethyl acetate extract of Cordyceps sinensis mycelium on various cancer cells in culture and B16 melanoma in C57BL/6 mice.	https://www.ncbi.nlm.nih.gov/pubmed/16608242	Y	2007	Jian Yong Wu
1057	Adrenal corticosteroids enhance production of type-C virus induced by 5-iodo-2'-deoxyuridine from cultured mouse fibroblasts.	https://pubmed.ncbi.nlm.nih.gov/4134726/	Y	1973	M Paran
1059	Incorporation of cordycepin (3'-deoxyadenosine) into ribonucleic acid and deoxyribonucleic acid of human tumor cells.	https://www.ncbi.nlm.nih.gov/pubmed/5861260	Y	1965	Joseph G Cory
1060	INHIBITION OF HUMAN TUMOR CELLS BY CORDYCEPIN.	https://www.ncbi.nlm.nih.gov/pubmed/14293694	Y	1965	Marvin A Rich
1061	In vitro RNA replication directed by replicase complexes isolated from the subgenomic replicon cells of hepatitis C virus	https://www.ncbi.nlm.nih.gov/pubmed/12525668	y	2003	Lai VC
1062	G-protein mediating the slow depolarization induced by FMRFamide in the ganglion cells of Aplysia	https://pubmed.ncbi.nlm.nih.gov/29264419/	y	1992	Chiba O
1063	Ribosomal binding and dipeptide formation by misacylated tRNA(Phe)	https://www.ncbi.nlm.nih.gov/pubmed/3061451	y	1988	Heckler TG
1064	Loss of positional specificity in the aminoacylation of Escherichia coli tRNAGly	https://www.ncbi.nlm.nih.gov/pubmed/6352702	y	1983	Ehrenfeld GM

1065	Adenosine and tubercidin binding and transport in Chinese hamster ovary and Novikoff rat hepatoma cells	https://www.ncbi.nlm.nih.gov/pubmed/6863404	y	1981	Plagemann PGW
1066	Specificity and sodium dependence of the active nucleoside transport system in choroid plexus	https://www.ncbi.nlm.nih.gov/pubmed/6699636	y	1984	Spector R
1067	Transfer RNA pyrophosphorolysis with CTP(ATP):tRNA nucleotidyltransferase. A direct route to tRNAs modified at the 3' terminus	https://www.ncbi.nlm.nih.gov/pubmed/6339494	y	1983	Francis TA
1069	DNA duplexes containing 3'-deoxynucleotides as substrates for DNA topoisomerase I cleavage and ligation	https://www.ncbi.nlm.nih.gov/pubmed/9575192	y	1998	Arslan T
1070	Adenosine inhibition of calmodulin-sensitive adenylate cyclase from bovine cerebral cortex	https://www.ncbi.nlm.nih.gov/pubmed/3486942	y	1986	Yeager RE
1071	The effects of purine nucleoside analogs on the response of the RIF-1 tumor to melphalan in vivo	https://www.ncbi.nlm.nih.gov/pubmed/3486861	y	1985	Horsman MR
1073	Chemotaxis and the synthesis of specific proteins are inhibited by 3-deazaadenosine and other adenosine analogs in a mouse macrophage cell line	https://www.ncbi.nlm.nih.gov/pubmed/6848495	y	1983	Aksamit RR
1075	Newly formed mRNA lacking polyadenylic acid enters the cytoplasm and the polyribosomes but has a shorter half-life in the absence of polyadenylic acid	https://www.ncbi.nlm.nih.gov/pubmed/6981059	y	1982	Zeevi M
1076	Comparison of the effects on cultured L1210 leukemia cells of the ribosyl, 2'-deoxyribosyl, and xylosyl homologs of tubercidin and adenosine alone or in combination with 2'-deoxycoformycin	https://www.ncbi.nlm.nih.gov/pubmed/6976834	y	1982	Cass CE
1078	Derivatives of 3'- and 5'-deoxyadenosine: their inhibitory activity against DNA viruses	https://www.ncbi.nlm.nih.gov/pubmed/6967001		1980	Grytzmann B
1079	Phenylalanyl-tRNA, lysyl-tRNA, isoleucyl-tRNA and arginyl-tRNA synthetases. Substrate specificity in the ATP/PPi exchange with regard to ATP analogs	https://www.ncbi.nlm.nih.gov/pubmed/6995115	y	1980	Freist W
1080	Both positional isomers of aminoacyl-tRNA's are bound by elongation factor Tu	https://www.ncbi.nlm.nih.gov/pubmed/378996	y	1979	Alford BL

1081	Transfer RNA control of the activation of isomeric tRNATrp's	https://www.ncbi.nlm.nih.gov/pubmed/378993	y	1979	Alford BL
1082	3'-deoxyadenosine and implantation of delayed blastocysts in mice	https://www.ncbi.nlm.nih.gov/pubmed/313973	y	1979	Fernandez-Noval A
1083	Selective Growth Inhibition of Sphaerotilus natans and Beggiatoa sp. by Nucleosides	https://www.ncbi.nlm.nih.gov/pubmed/16345327	y	1978	Takiguchi Y
1085	2'-Versus 3'-OH specificity in tRNA aminoacylation. Further support for the "secondary cognition" proposal	https://www.ncbi.nlm.nih.gov/pubmed/353043	y	1978	Alford BL
1086	DNA-dependent single-step addition reactions catalyzed by Escherichia coli RNA polymerase	https://www.ncbi.nlm.nih.gov/pubmed/347447	y	1978	Oen H
1087	Initial position of aminoacylation of individual Escherichia coli, yeast, and calf liver transfer RNAs	https://www.ncbi.nlm.nih.gov/pubmed/319826	y	1977	Chinault AC
1088	Preparation of Escherichia coli tRNAs terminating of modified nucleosides by the use of CTP(ATP):tRNA nucleotidyltransferase and polynucleotide phosphorylase	https://www.ncbi.nlm.nih.gov/pubmed/319825	y	1977	Chinault AC
1089	Isomeric aminoacyl-tRNAs are both bound by elongation factor Tu	https://www.ncbi.nlm.nih.gov/pubmed/322124	y	1977	Hecht SM
1090	Inhibition of protein kinase activity from Trypanosoma cruzi and Trypanosoma gambiense by 3'-deoxyadenosine	https://www.ncbi.nlm.nih.gov/pubmed/320116	y	1977	Walter RD
1091	Thyroid hormone stimulates de novo growth hormone synthesis in cultured GH1 cells: evidence for the accumulation of a rate limiting RNA species in the induction process	https://www.ncbi.nlm.nih.gov/pubmed/185609	y	1976	Samuels HH
1092	Hydrolytic action of aminoacyl-tRNA synthetases from baker's yeast. "Chemical proofreading" of Thr-tRNA Val by valyl-tRNA synthetase studied with modified tRNA Val and amino acid analogues	https://www.ncbi.nlm.nih.gov/pubmed/322705	y	1977	Igloi GF
1093	Hydrolytic action of aminoacyl-tRNA synthetases from baker's yeast: "chemical proofreading" preventing acylation of tRNA(I1e) with misactivated valine	https://www.ncbi.nlm.nih.gov/pubmed/786367	y	1976	von der Haar F

1094	Cordycepin induces apoptosis in human bladder cancer T24 cells through ROS-dependent inhibition of the PI3K/Akt signaling pathway.	https://www.ncbi.nlm.nih.gov/pubmed/31527329	y	2019	Kim SO
1095	The Inhibitory Effect of Cordycepin on the Proliferation of MCF-7 Breast Cancer Cells, and its Mechanism: An Investigation Using Network Pharmacology-Based Analysis.	https://www.ncbi.nlm.nih.gov/pubmed/31454995	y	2019	Lee D
1097	Molecular networking as a dereplication strategy for monitoring metabolites of natural product treated cancer cells.	https://www.ncbi.nlm.nih.gov/pubmed/31411772	y	2019	Gao Y
1100	Cordycepin Induces Apoptosis and G2/M Phase Arrest through the ERK Pathways in Esophageal Cancer Cells	https://www.ncbi.nlm.nih.gov/pubmed/31258746	y	2019	Xu J
1101	Cordycepin alleviates hepatic lipid accumulation by inducing protective autophagy via PKA/mTOR pathway	https://www.ncbi.nlm.nih.gov/pubmed/31242974	y	2019	Li T
1102	Cordycepin ameliorates cardiac hypertrophy via activating the AMPKα pathway.	https://www.ncbi.nlm.nih.gov/pubmed/31225721	y	2019	Wang H
1104	Cordycepin kills Mycobacterium tuberculosis through hijacking the bacterial adenosine kinase	https://www.ncbi.nlm.nih.gov/pubmed/31199855	y	2019	Huang F
1105	Cordycepin prevents radiation ulcer by inhibiting cell senescence via NRF2 and AMPK in rodents.	https://www.ncbi.nlm.nih.gov/pubmed/31182708	y	2019	Wang Z
1106	Cordycepin-induced unfolded protein response-dependent cell death, and AKT/MAPK-mediated drug resistance in mouse testicular tumor cells	https://www.ncbi.nlm.nih.gov/pubmed/31145545	y	2019	Chang M
1107	Metabolic profiling of natural and cultured Cordyceps by NMR spectroscopy.	https://www.ncbi.nlm.nih.gov/pubmed/31118439	y	2019	Lu Y
1108	A natural cordycepin/chitosan complex hydrogel with outstanding self-healable and wound healing properties.	https://www.ncbi.nlm.nih.gov/pubmed/31067486	y	2019	Song R
1109	Cordyceps militaris Improves Chronic Kidney Disease by Affecting TLR4/NF-κB Redox Signaling Pathway.	https://www.ncbi.nlm.nih.gov/pubmed/31049139	y	2019	Sun T

1110	Requirements for accurate and efficient mRNA 3' end cleavage and polyadenylation of a simian virus 40 early pre-RNA in vitro	https://www.ncbi.nlm.nih.gov/pubmed/3031477	y	1987	Ryner LC
1111	A new ribonucleotide from Cordyceps militaris	https://www.ncbi.nlm.nih.gov/pubmed/28503945	y	2017	Sun J
1112	Cordycepin induces apoptosis in human tongue cancer cells in vitro and has antitumor effects in vivo	https://pubmed.ncbi.nlm.nih.gov/32730909/	y	2020	Zheng Q
1113	Cordycepin attenuates Salivary Hypofunction through the Prevention of Oxidative Stress in Human Submandibular Gland Cells	https://pubmed.ncbi.nlm.nih.gov/32714076/	y	2020	Jaiboonma A
1118	Cordycepin Nanoencapsulated in Poly(Lactic-Co-Glycolic Acid) Exhibits Better Cytotoxicity and Lower Hemotoxicity Than Free Drug	https://pubmed.ncbi.nlm.nih.gov/32606622/	y	2020	Marslin G
1122	Chemical perturbations reveal that RUVBL2 regulates the circadian phase in mammals	https://pubmed.ncbi.nlm.nih.gov/32376767/	y	2020	Ju D
1126	Xylitol acts as an anticancer monosaccharide to induce selective cancer death via regulation of the glutathione level	https://pubmed.ncbi.nlm.nih.gov/32275922/	y	2020	Tomonobu N
1127	Cordycepin protects against acute pancreatitis by modulating NF-κB and NLRP3 inflammasome activation via AMPK	https://pubmed.ncbi.nlm.nih.gov/32268154/	y	2020	Yang J
1128	Cordycepin Attenuates IFN-γ-Induced Macrophage IP-10 and Mig Expressions by Inhibiting STAT1 Activity in CFA-Induced Inflammation Mice Model	https://pubmed.ncbi.nlm.nih.gov/31873836/	y	2020	Yang R
1129	Natural cordycepin induces apoptosis and suppresses metastasis in breast cancer cells by inhibiting the Hedgehog pathway	https://pubmed.ncbi.nlm.nih.gov/32163051/	y	2020	Liu C
1131	Yarsagumba is a Promising Therapeutic Option for Treatment of Pulmonary Hypertension due to the Potent Anti-Proliferative and Vasorelaxant Properties	https://pubmed.ncbi.nlm.nih.gov/32188043/	y	2020	Luitel H
1132	Cordycepin Resensitizes T24R2 Cisplatin-Resistant Human Bladder Cancer Cells to Cisplatin by Inactivating Ets-1 Dependent MDR1 Transcription	https://pubmed.ncbi.nlm.nih.gov/32131547/	y	2020	Oh S

1133	Cordycepin exhibits a suppressive effect on T cells through inhibiting TCR signaling cascade in CFA-induced inflammation mice model	https://pubmed.ncbi.nlm.nih.gov/32105161/	y	2020	Wang X
1134	Cordycepin Inhibits Cancer Cell Proliferation and Angiogenesis through a DEK Interaction via ERK Signaling in Cholangiocarcinoma	https://pubmed.ncbi.nlm.nih.gov/32102917/	y	2020	Liu T
1135	Cordycepin promotes osteogenesis of bone marrow-derived mesenchymal stem cells and accelerates fracture healing via hypoxia in a rat model of closed femur fracture	https://pubmed.ncbi.nlm.nih.gov/32084699/	y	2020	Li Z
1137	Probing Carbon Utilization of Cordyceps militaris by Sugar Transportome and Protein Structural Analysis	https://pubmed.ncbi.nlm.nih.gov/32050592/	y	2020	Sirithep K
1138	Cordycepin protects renal ischemia/reperfusion injury through regulating inflammation, apoptosis, and oxidative stress	https://pubmed.ncbi.nlm.nih.gov/31951250/	y	2020	Han F
1139	Sensitive and Selective Measurement of Hydroxyl Radicals at Subcellular Level with Tungsten Nanoelectrodes	https://pubmed.ncbi.nlm.nih.gov/31927939/	y	2020	Ding S
1140	Impact of fullerol C 60(OH) 24 nanoparticles on the production of emerging toxins by Aspergillus flavus	https://pubmed.ncbi.nlm.nih.gov/31959903/	y	2020	Kovač T
1141	Exploring the binding modes of cordycepin to human adenosine deaminase 1 (ADA1) compared to adenosine and 2'-deoxyadenosine	https://pubmed.ncbi.nlm.nih.gov/31953681/	y	2020	Niramitranon J
1142	A rapid assay to screen adenosine deaminase inhibitors from Ligustri Lucidi Fructus against metabolism of cordycepin utilizing ultra-high-performance liquid chromatography-tandem mass spectrometry	https://pubmed.ncbi.nlm.nih.gov/31845520/	y	2020	Guan H
1143	Effects of Substrates on the Production of Fruiting Bodies and the Bioactive Components by Different Cordyceps militaris Strains (Ascomycetes)	https://pubmed.ncbi.nlm.nih.gov/32463998/		2020	Tao S
1147	Combining tubercidin and cordycepin scaffolds results in highly active candidates to treat late-stage sleeping sickness	https://pubmed.ncbi.nlm.nih.gov/31804484/	y	2019	Hulpia F
1150	Cordycepin inhibits human ovarian cancer by inducing autophagy and apoptosis through Dickkopf-related protein 1/β-catenin signaling	https://pubmed.ncbi.nlm.nih.gov/31814895/	y	2019	Jang H

1152	Cordycepin suppresses the migration and invasion of human liver cancer cells by downregulating the expression of CXCR4	https://pubmed.ncbi.nlm.nih.gov/31746344/	y	2019	Guo Z
1153	A novel nucleoside rescue metabolic pathway may be responsible for therapeutic effect of orally administered cordycepin	https://pubmed.ncbi.nlm.nih.gov/31673018/	y	2019	Lee JB
1155	Cordycepin Enhances Radiosensitivity in Oral Squamous Carcinoma Cells by Inducing Autophagy and Apoptosis Through Cell Cycle Arrest	https://pubmed.ncbi.nlm.nih.gov/31661901/	y	2019	Ho S
1156	Cordycepin protects against β-amyloid and ibotenic acid-induced hippocampal CA1 pyramidal neuronal hyperactivity	https://pubmed.ncbi.nlm.nih.gov/31680770/	y	2019	Yao L
1162	Cordycepin improves behavioral-LTP and dendritic structure in hippocampal CA1 area of rats	https://pubmed.ncbi.nlm.nih.gov/31314908/	y	2019	Han Y
1163	The protective effect of Cordycepin on diabetic nephropathy through autophagy induction in vivo and in vitro	https://pubmed.ncbi.nlm.nih.gov/31359358/	y	2019	Cao T
1164	Cordycepin (3'-deoxyadenosine) promotes remyelination via suppression of neuroinflammation in a cuprizone-induced mouse model of demyelination	https://pubmed.ncbi.nlm.nih.gov/31357085/	y	2019	Jia Y
1165	In Vitro Nociceptor Neuroplasticity Associated with In Vivo Opioid-Induced Hyperalgesia	https://pubmed.ncbi.nlm.nih.gov/31300521/	y	2019	Khomula E
1169	Cordycepin inhibits pancreatic cancer cell growth in vitro and in vivo via targeting FGFR2 and blocking ERK signaling	https://pubmed.ncbi.nlm.nih.gov/32451092/	y	2020	Li Y
1170	Cordycepin regulates body weight by inhibiting lipid droplet formation, promoting lipolysis and recruiting beige adipocytes	https://pubmed.ncbi.nlm.nih.gov/31259423/	y	2019	Xu H
1172	Mechanism of Activation of AMPK by Cordycepin	https://pubmed.ncbi.nlm.nih.gov/31991096/	y	2020	Hawley SA
1173	The novel application of cordycepin in maintaining stem cell pluripotency and increasing iPS cell generation efficiency	https://pubmed.ncbi.nlm.nih.gov/32042022/	y	2020	Wang CH

1175	Opioid-Induced Hyperalgesic Priming in Single Nociceptors	https://pubmed.ncbi.nlm.nih.gov/33203743/	y	2020	Khomula EV
1177	Anti-Cancer Effect of Cordycepin on FGF9-Induced Testicular Tumorigenesis	https://pubmed.ncbi.nlm.nih.gov/33172093/	y	2020	Chang M
1178	Distinct Poly(A) nucleases have differential impact on sut-2 dependent tauopathy phenotypes	https://pubmed.ncbi.nlm.nih.gov/33184027/	y	2020	Kow RL
1180	Cordycepin enhances the chemosensitivity of esophageal cancer cells to cisplatin by inducing the activation of AMPK and suppressing the AKT signaling pathway	https://pubmed.ncbi.nlm.nih.gov/33067427/	yes	2020	Gao Y
1182	Cordycepin, a metabolite of Cordyceps militaris, reduces immune-related gene expression in insects	https://pubmed.ncbi.nlm.nih.gov/33022282/	y	2020	Woolley VC
1183	Improvement of the multi-performance biocharacteristics of cordycepin using BiloNiosome-core/chitosan-shell hybrid [illegible]	https://pubmed.ncbi.nlm.nih.gov/33032178/	y	2020	Kengkittipat W
1184	Cordycepin-loaded Nanoparticles from Cassava Starch Promote the Proliferation of Submandibular Gland Cells and Inhibit the Growth of Oral Squamous Carcinoma Cells	https://pubmed.ncbi.nlm.nih.gov/32929998/	y	2020	Kaokaen P
1185	Combination of Cordycepin and Apatinib Synergistically Inhibits NSCLC Cells by Down-Regulating VEGF/PI3K/Akt Signaling Pathway	https://pubmed.ncbi.nlm.nih.gov/33014856/	y	2020	Liao X
1190	A Comparative Study on 5hmC Targeting Regulation of Neurons in AD Mice by Several Natural Compounds	https://pubmed.ncbi.nlm.nih.gov/32802849/	y	2020	Cao D
1191	Cordycepin Inhibits Human Gestational Choriocarcinoma Cell Growth by Disrupting Centrosome Homeostasis	https://pubmed.ncbi.nlm.nih.gov/32801639/	y	2020	Wang C
1195	Cordycepin: a bioactive metabolite of Cordyceps militaris and polyadenylation inhibitor with therapeutic potential against COVID-19	https://pubmed.ncbi.nlm.nih.gov/33225826/	y	2020	Verma AK
1198	Repurposing potential of FDA-approved and investigational drugs for COVID-19 targeting SARS-CoV-2 spike and main protease and validation by machine learning algorithm	https://pubmed.ncbi.nlm.nih.gov/33289334/	y	2020	Verma AK

1200	Protective effect of cordycepin on experimental renal ischemia/reperfusion injury in rats	https://pubmed.ncbi.nlm.nih.gov/33348963/	y	2020	Aydin HR
1201	Cordycepin attenuates high-fat diet-induced non-alcoholic fatty liver disease via down-regulation of lipid metabolism and inflammatory responses	https://pubmed.ncbi.nlm.nih.gov/33352441/	y	2021	Gong X
